# Deriving a Deployment Threshold for Benefit and Harm in Clinical AI: Multi-Institutional Decision-Analytic Modeling Study

**DOI:** 10.2196/109863

**Published:** 2026-10-23

**Authors:** Yngve Mikkelsen

**Affiliations:** 1 Saïd Business School University of Oxford Oxford, England United Kingdom

**Keywords:** clinical decision support, health technology assessment, AI, deployment, local validation, retrieval-augmented generation

## Abstract

**Background:**

Clinical AI operates in a health care environment heterogeneous in biology, clinical practice, and documentation, so its benefit-harm balance is deployment-specific: an intervention can help in one setting and harm in another when the determinant of response is not routinely observed.

**Objective:**

This study had 2 aims: to derive the deployment threshold at which an AI intervention whose effect changes sign becomes beneficial, using zero-phase component analysis whitening (a retraining-free correction to clinical retrieval embeddings) as a worked case, and to determine whether the evidence needed to apply that threshold exists in the published literature or must be measured locally.

**Methods:**

We specified a 3-arm decision-analytic model—no action, universal deployment, and screen-and-treat—for interventions with sign-changing effects and instantiated it on a retraining-free correction to clinical retrieval embeddings. Empirical effect groups were defined by measured response rather than nominal mechanistic tier. Screening accuracy was derived from between-condition variability and tested by local resampling. Transportability was evaluated on emergency-department notes and discharge summaries from 2 US academic centers. A structural sensitivity analysis allowed the downstream value of benefit and harm to differ.

**Results:**

Measured response defined 6 benefited and 7 harmed configurations at the base-case regularization. The universal-deployment threshold was p*=0.50; the exact and zero-cost forms differ by 2.8×10^–5^, so strategy cost had a negligible effect on it, and across 6 regularization settings, the threshold ranged from 0.32 to 0.70. Published descriptions did not identify the response-defined state: none of the 55 published systems had been evaluated under the intervention, so response prevalence remained unidentified without classification assumptions. Local screening reduced the minimum required prior probability from 0.210 with 1 scored condition to 0.071 with 6. The nominal mechanistic tier agreed with response in all 52 institutional section-level cells, but 1 benchmark-harmed configuration benefited in all 4 institutional conditions. In the joint fit-and-evaluation resampling protocol, specificity rose from 0.328 at 10 documents to 0.974 at 75 documents.

**Conclusions:**

The deployment threshold is analytically identifiable, but the state needed to apply; it cannot be recovered reliably from model identity or the published literature. Deployment validity is therefore local in the setting examined: local measurement is needed when the response-defining state cannot otherwise be established reliably before deployment, and an unsuitable measured state requires local action rather than plug-and-play transfer. The data do not identify any single adaptation method as universally necessary.

## Introduction

Health technology assessment asks whether expected benefit, harm, and resource use justify adoption in the population that will receive a technology.

Clinical AI adds deployment heterogeneity. In clinical retrieval, corpus and query context can explain as much performance variance as model choice [1], while other failures localize to the geometry of the extracted representation [2]. An intervention targeting one failure mode can therefore help one deployment and harm another despite similar system descriptions.

The analogous stratification variable may therefore be a latent property of the deployed representation rather than a recorded patient characteristic. The key empirical question is whether that property can be recovered from published descriptions or must be measured locally.

Low implementation cost can obscure this question: the correction studied here costs about €1468 over 5 years against €344 million of modeled value exposed (all monetary values are reported in 2024 euros and Norwegian kroner; for reference, €1=US $1.1181 and NOK 1=US $0.0962 at the European Central Bank reference rate of October 12, 2026).

Clinical retrieval-augmented generation (RAG) aims to surface relevant record information at the point of decision. We use a retraining-free correction to clinical retrieval embeddings that produced sign-changing effects across configurations [2]. The correction is the empirical instrument, not the general subject; its measurable technical outcome and low direct cost make the deployment problem tractable. Reviews show rapid growth of clinical RAG while retrieval design, reporting, and clinical validation remain unresolved [3,4].

Economic evaluations of health care AI have focused mainly on clinically visible applications, especially imaging, and a 2026 review identified none for generative AI [5-7]. No economic evaluation of a component-level correction was found for a clinical AI system.

For component-level interventions, downstream clinical and economic effects are deployment-specific. Developmental and Exploratory Clinical Investigations of Decision support systems driven by Artificial Intelligence places early live clinical evaluation before large trials [8]; here, a logically prior question is whether the technical intervention is beneficial in the setting at all.

A decision model is required for 3 reasons. The deployment choice—universal application, no action, or selective use—needs an explicit threshold; the clinical and economic translation parameters lack published estimates, so their decision relevance must be tested; and negligible acquisition cost otherwise removes the procurement friction that would force appraisal. No cost-effectiveness ratio is claimed, and absolute monetary values are scenario quantities.

Figure 1 summarizes the evidence sequence: derive the deployment condition for sign-changing effects, test whether the required response state can be identified externally or must be measured locally, and separate absolute downstream scale from relative benefit-harm translation.

**Figure 1:**
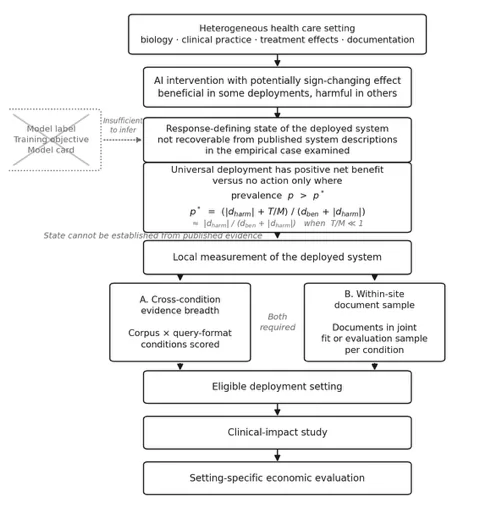
Evidence sequence for deployment of a clinical AI intervention with heterogeneous effects. The response-defining state of a deployed system determines whether the intervention has positive net benefit versus no action, but was not recoverable from published system descriptions in the case examined; declarative routes through the model label, training objective, or model card are therefore insufficient. Local measurement substitutes for declarative classification, subject to 2 distinct design axes: cross-condition evidence breadth and the local document sample used jointly for transform fitting and effect evaluation in the present resampling experiment. The latter does not establish separate universal requirements for transform-fitting and evaluation sample sizes. T denotes deployment cost and M the common absolute value scale in the base case.

## Methods

### Decision Problem

Three strategies were compared over 5 years from a Norwegian health-system perspective, discounted at 4% in line with current Norwegian health-economic guidance [9]: A no action, B universal deployment, and C screen-and-treat, in which each site measures the correction’s effect on its own corpus and applies it only where the measured effect is beneficial.

Let an intervention produce benefit *d*_ben>0 in a response-defined subgroup of prevalence *p* and harm *d*_harm<0 outside it. Net technical effect is *p*·*d*_ben + (1 – *p*)·*d*_harm for universal deployment and *p*·se·*d*_ben + (1 – *p*)·(1 – sp)·*d*_harm for screen-and-treat, where se and sp are local screen sensitivity and specificity, respectively.

### The General Threshold

Net monetary benefit for each strategy takes the form:

NB = net_effect × *M* – *K*
**(1)**

where *K* is strategy-specific cost, and *M* is the monetary value exposed to the intervention:

*V* = *C*_event + λ·*Q*_event (monetary value of one averted event) **(2)**

*M* = α·adoption·*p*_event·*N*·*V*·*D* (value exposed to the intervention) **(3)**

with α the mapping from technical metric to decision accuracy, *N* annual retrievals, λ the willingness-to-pay threshold, and *D* the discount factor. Note that *C*_event and *Q*_event are not independent multipliers: they combine into the single positive scalar *V*, and it is *V* that enters *M*.

Setting NB_B>NB_A and solving for *p* gives the exact threshold:

p* = (|*d*_harm| + *K*/*M*)/(*d*_ben + |*d*_harm|) **(4)**

which reduces to |*d*_harm|/(*d*_ben + |*d*_harm|) only in the limit *K*/*M*→0. That limit is not assumed here; it is established empirically in the Results section.

The common-multiplier specification is a base case, not a universal property. If benefit and harm translate to downstream value through different positive multipliers *M*_ben and *M*_harm, universal-deployment net benefit is *p*·*d*_ben·*M*_ben – (1 – *p*)·|*d*_harm|·*M*_harm – *K*. Defining *r* = *M*_ben/*M*_harm gives p* = (|*d*_harm| + *K*/*M*_harm)/(*r*·*d*_ben + |*d*_harm|). The common-*M* model is the special case *r*=1. Absolute common scaling cannot reorder strategies when *K*/*M* is negligible, but the relative translation *r* can.

*K* remains outside the multiplier, and its irrelevance is empirical rather than structural: uncertainty in *K* cannot change the decision here only because *K*/*M* is orders of magnitude below the decision-changing region. The distinction between absolute scale and relative benefit-harm translation is preserved throughout.

### Instantiation

The model was instantiated on corpus-only zero-phase component analysis (ZCA) whitening, a retraining-free geometric correction evaluated across 13 transformer configurations, 3 clinical corpora, and 2 query formats in the published companion study [2]. Associated benchmark, software, and analysis archives provide reproducibility materials [10-12].

The companion study reports full-corpus and 5-fold cross-validated protocols [2,12]. The deployment-matched full-corpus protocol is the base case. At ε=10^–5^, measured response defines 6 benefited configurations (mean ΔMRR [mean reciprocal rank] @10 +0.075745) and 7 harmed configurations (–0.076593); E5-Mistral-7B-ablation is reassigned to harmed because its benchmark mean is –0.015669. Nominal tiers are used only for mechanistic description and transport comparisons; cross-validated estimates are excluded for the bias described in the Strengths and Limitations section.

The correction acts at the retrieval layer and informs rather than determines clinical management: the clinician retains the care decision. The adoption parameter represents the probability that retrieved output is acted upon. The correction is locked, with fixed post hoc transformation parameters, and no performance change over time is assumed.

An independent study in semantic code search reported a similar sign-flip structure: standard ZCA whitening improved pretrained code language models substantially while degrading contrastively fine-tuned variants on most datasets [13]. That experiment was independently replicated here as a boundary test of the framework. As reported in the Results section, baseline retrieval reproduced closely, but the published mechanistic grouping did not, and the presence of sign-changing heterogeneity depended on regularization; the framework’s antecedent is therefore treated as an empirical condition rather than inferred from model class.

### Deployment Scale

Statistics Norway recorded 2,656,857 patients in somatic specialist health care in 2024 [14]. The modeled deployment of 133,000 annual retrievals is 1 retrieval per patient per year at an institution serving approximately one-twentieth of that activity—roughly one health trust.

### Subgroup Prevalence: What Can and Cannot Be Identified

A rule-based frame of 55 clinical RAG systems from 2 systematic reviews [3,4] was coded for implementation characteristics hypothesized to correspond to response. Systems were coded as affected only where both the retrieval representation and the absence of retrieval-specific calibration could be established from the published description; all other systems were coded as unknown.

The frame samples publications, not deployments. None of the 55 systems reported pooling as such, only 1 allowed extraction to be inferred, and 27 used commercial embedding interfaces with undisclosed geometry; unknown systems therefore cannot be treated as unaffected.

The theorem’s *p* is response-defined prevalence, but none of the 55 systems was evaluated under the intervention. All frame coding is mechanistic, which matters because one configuration classified mechanistically as benefited measured negative in the base-case protocol.

Without a validated link between observable implementation characteristics and response, the no-assumption bound on *p* is 0-1. Proxy partial-identification intervals were calculated under 4 prespecified assumptions: none, contrastive training implies nonmembership for self-hosted systems, vendor optimization claims are accepted for commercial interfaces, and the same rule after excluding 3 nondense retrievers. The 1/55 mechanistic count is descriptive only.

For population policy, *p* is prevalence of the benefited response state; for a site, the same algebra uses its prior probability of benefit. No transportable distribution for either quantity is inferred from the publication frame.

### Screening Thresholds

Screening cost is separated into a fixed component and a conditional one, because the correction is deployed only where the screen indicates it. Let *S* be the cost of scoring the screen, incurred whichever way it reads, and *T* the deployment cost, incurred only on the systems the screen selects. Writing *s*=*S*/*M*, *t*=*T*/*M*, and *h*=|*d*_harm|, screen-and-treat is preferred to no action, where *M*[*p*·se·*d*_ben – (1 – *p*)(1 – sp)*h*] – *S* – *T*[*p*·se + (1 – *p*)(1 – sp)]>0, giving the exact specificity threshold:

sp* = 1 – [*p*·se(*d*_ben – *t*) – *s*]/[(1 – *p*)(*h* + *t*)] **(5)**

Separating the 2 matters because the deployment cost is incurred at a rate that itself depends on *p*; folding it into a single strategy cost would require assuming a value of *p*, which the identification analysis declines to do. As with the deployment threshold, the cost terms are retained rather than assumed away and shown negligible in the Results section.

This expression is conditional on *p* for a population policy. For an individual site, it is evaluated with that site’s prior probability of benefit. The condition is therefore inverted, solving for the minimum prior probability at which a screen of given accuracy has positive net effect:

*p*_screen* = [(1 – sp)(*h* + *t*) + *s*]/[se(*d*_ben – *t*) + (1 – sp)(*h* + *t*)] **(6)**

which reduces to (1 – sp)*h*/[se*d_ben + (1 – sp)*h*] as *s* and *t* approach 0. The exact form is used. This inversion does not eliminate the need for prior information; it quantifies how strong the prior probability of benefit must be before a screen of given accuracy has positive expected value.

The screen is a local measurement rather than proxy classification: institutional documents are sampled, the transform is fitted and applied, retrieval is scored against metadata-derived known-item queries, and the correction is adopted if the mean change is positive. It operates on returned vectors without model weights or vendor disclosure.

Benchmark screening accuracy was defined as the probability that a finite sample recovers the correct sign. Each configuration was assigned by the sign of its 6-condition mean at ε=10^–5^; accuracy was derived from the configuration-specific mean and between-condition SD across 78 observations (13 configurations×6 corpus-by-query-format conditions), with SE=SD/√n.

Published SEs were not used because 5 folds partition the same 100 documents. Clustering on condition inflates the SE by a mean factor of 2.02 (range 1.69-2.23), making condition the unit of independent information.

Only 6 independent corpus-by-query-format conditions were observed, so cross-condition results are limited to 1 through 6 conditions. Condition count denotes evidence breadth; document count denotes the within-site sample used to fit and evaluate a local screen.

### Corpora for the Transport Evaluation

The benchmark derivation used 3 corpora—MTSamples, PMC-Patients, and a synthetic corpus—crossed with 2 query formats. Transport was tested on 2 institutional corpora differing in institution, documentation genre, and deidentification regime.

At a fixed seed, 100 unstratified documents of at least 400 characters were sampled from ER-Reason (version 1.0.0) emergency-department notes from University of California, San Francisco (UCSF) [15,16] and MIMIC (Medical Information Mart for Intensive Care)-IV-Note (version 2.2) discharge summaries from Beth Israel Deaconess Medical Center [16,17].

Because these corpora are much longer than the benchmark and model context windows vary systematically by nominal tier, native whole-note encoding would confound document exposure with the effect under test. The primary analysis therefore used presenting-narrative sections fully encoded by all configurations; a whole-note sensitivity analysis capped every configuration at 512 tokens.

Section extraction succeeded in all 200 documents using corpus-specific headers; exact extraction rules and the complete transport matrix are provided in Multimedia Appendix 1.

Uniform 512-token whole-note encoding was used only as a sensitivity analysis; native-limit encoding was not used because unequal context windows align with the nominal mechanistic tier.

Screen accuracy was also measured directly. For each configuration and corpus, the full-sample effect defined the reference sign; 500 subsamples of n∈{10, 20, 30, 50, 75} documents were drawn without replacement, whitening was refitted on each subsample, and sign agreement was recorded. Thin-singular value decomposition whitening was used because the high-dimensional subsample covariance is rank deficient; it reproduced full-covariance results to numerical precision. Implementation details are in Multimedia Appendix 2.

### Query Generation for the External Corpora

Data-use agreements precluded sending either institutional corpus to a commercial API, so metadata-derived queries following the benchmark protocol [1,10] were generated locally with qwen2.5:14b-instruct [18]. Metadata were extracted first, and queries were generated from metadata alone; the complete protocol is in Multimedia Appendix 3.

In addition to the required model substitution, 3 protocol modifications were explicit. JSON output prevented prose leakage. An instruction to use only supplied values, with missing age normalized to the protocol’s “adult” default, reduced invented ages to 2 of 100 and 1 of 100 documents. Outputs were also truncated at the first non–American Standard Code for Information Interchange (ASCII) character because the local model occasionally changed language midgeneration.

Metadata extraction parsed successfully for 100 of 100 documents in both corpora, and primary diagnosis was non-unknown in all 200 documents. As a grounding check available only for ER-Reason, extracted primary diagnoses were compared against the registry diagnosis field, which the extraction stage never sees; disagreements were inspected and reflected presentation-versus-final-diagnosis differences (“acute abdominal pain, concern for pyelonephritis” against “complicated urinary tract infection”) and vocabulary differences (“dermatomal vesicular rash, suspected shingles” against “ophthalmic herpes zoster”), not fabrication.

To bound this query-generator substitution, the panel was rerun on the benchmark MTSamples sample with both the published queries and locally generated queries, holding documents, configurations, and all other settings constant. Agreement was assessed by configuration ranking and subgroup assignment.

### Scope of the Framework

The framework applies only when an intervention’s effect changes sign across response-defined subgroups. Whether that antecedent holds must be established empirically in each domain. In the code-search replication reported in the Results section, it held only at low regularization values, and the subgroup structure reported in the published account did not reproduce. Uniformly beneficial or harmful effects do not create the stratification problem addressed here. Where sign-changing heterogeneity exists, as in the clinical case, the deployment condition is analytic; whether its required state is observable remains empirical.

### Parameters and Reporting

The economic scale used national inpatient resource-use estimates and literature-based adverse-event costs [19,20]. A Norwegian somatic bed-day cost of NOK 26,153 [21] multiplied by 5.11 excess days [22] gives NOK 133,642 per event. At the 2024 Norges Bank annual average of 11.6276 NOK/EUR [23], the adverse-event cost is therefore €11,494. The NOK 275,000 per quality-adjusted life year (QALY) willingness-to-pay threshold [24] converts to €23,651; NOK 825,000 is carried as a severity-weighted scenario. Utility loss is scenario-based [25].

Strategy costs are scenario inputs in 2024 euros. Deployment costs €800 once plus €150 annually, discounted at 4% over 5 years (*D*=4.4518), giving *T*=€1468. Screening costs €400 per scored condition, so *S*=€1600 at 4 conditions. *S* is always incurred; *T* only when the screen selects deployment. Both enter only through ratios to value exposed.

Three parameters entering *M*—mapping from technical metric to decision accuracy, adoption, and event probability conditional on an adopted erroneous retrieval—have no published source and are explicitly illustrative. Absolute event counts are therefore scenario-derived and are not primary evidence.

The event modeled is a downstream adverse clinical event rather than a medication event specifically. Reporting follows CHEERS (Consolidated Health Economic Evaluation Reporting Standards) 2022 and CHEERS-AI (Consolidated Health Economic Evaluation Reporting Standards for Interventions That Use Artificial Intelligence) [26,27], with completed checklists in Multimedia Appendices 4 and 5.

### Analysis

Primary analysis used exact algebraic thresholds with empirical response magnitudes. Sensitivity varied regularization over 6 observed values, value per averted event over the economic scenario range, and the benefit-harm translation ratio *r* over 0.25, 0.5, 1, 2, and 4. Screening accuracy was derived from between-condition variability and measured directly by local resampling. Because no empirical distribution for *p* is identified, population strategy probabilities and monetary value-of-information estimates are not primary results.

Further analyses varied the 3 illustrative parameters entering *M* one at a time over their 2.5th-97.5th percentiles and jointly in a 50,000-draw probabilistic analysis with cost and utility parameters. Horizon was varied from 1 to 10 years at 0%, 3%, and 4% discount rates; adverse-event cost across lower European estimates [19,20]; and exchange rate across completed Norges Bank annual averages.

The classification rule was also varied. A configuration is assigned to the harmed group if its mean effect across scored conditions is negative; because that boundary sits at exactly 0, the rule was swept over ±0.05 in mean ΔMRR@10 and the threshold recomputed at each step, so the consequence of the boundary choice is reported as a function rather than a single comparison.

No sampling interval is placed on the threshold. The 13 configurations are a purposive panel rather than a probability sample of deployments, and the response groups are defined from the observed sign of the same effects used to compute their means, so an interval conditional on that classification would have no frequentist interpretation for any deployment population. Structural sensitivity is reported instead.

### Ethical Considerations

This study is a secondary analysis of published system descriptions and existing deidentified clinical document corpora. No new data were collected, no participants were recruited or contacted, and no identifiable private information was accessed. Only aggregate results are reported. The institutional corpora are credentialed resources. MIMIC-IV-Note (version 2.2) is distributed under the PhysioNet Credentialed Health Data Use Agreement; its source collection and resource creation were reviewed by the Beth Israel Deaconess Medical Center Institutional Review Board, which granted a waiver of informed consent and approved data sharing. ER-Reason v1.0.0 was collected and deidentified by the UCSF Information Commons team, with sharing approved by the institutional compliance team. Access to both required credentialing and training. The 3 benchmark corpora used for the derivation carry no credentialing requirement: MTSamples and PMC-Patients are publicly distributed deidentified transcription and case-report collections, and the synthetic corpus contains no patient-derived text. All processing of the credentialed corpora was performed locally by the credentialed author. No note text was transmitted to any third-party service; queries for these corpora were generated locally for this reason, as described earlier. All corpora are deidentified at source, no reidentification was attempted, and no linkage to external data was performed. No participants were recruited, and no compensation was provided. The manuscript and its supplementary material contain no images of individuals and no material from which any individual could be identified. Because the work used only existing deidentified data and published materials, no additional ethics-review application was submitted by the author, and no application or case number therefore exists. The governance basis for the credentialed corpora is the access controls, data use agreements, deidentification, and original institutional approvals provided by the respective data custodians.

## Results

### The Threshold, Exactly and in the Limit

Under base-case effect estimates, the exact threshold is:

p*=(0.076593+4.3×10^–6^)/(0.075745+0.076593)=0.502810 **(7)**

against a zero-cost limit of 0.502782—a difference of 2.8×10^–5^. The 6-figure values show only that the cost term has a negligible effect at the reported precision; they are not an estimate of its precision, and both are evaluated on the unrounded group means rather than the rounded terms displayed. Sampling precision is not estimated: the 13 configurations are a purposive panel rather than a probability sample, and the response groups are defined by the observed sign of the same effects used to compute their means, so an interval conditional on that classification would have no frequentist interpretation for any deployment population. The threshold is reported as 0.50 throughout, with structural sensitivity from the regularization sweep: across the 6 observed settings, response-defined thresholds ranged from 0.32 to 0.70 (Multimedia Appendix 6). Effect sizes are the unrounded per-condition means after response-defined regrouping at ε=10^–5^. The value of one averted event is *V*=€11,529; the value exposed is *M*=€344.0 million, against a 5-year deployment cost of *T*=€1468, giving *T*/*M*=4.3×10^–6^. The absolute economic values are scenario quantities; the threshold is driven by the measured benefit-harm balance and the demonstrated negligible cost ratio.

Implementation cost is negligible here by demonstration, not assumption: *K*/*M*=4.3×10^–6^ (*M*/*K* approximately 234,000). This limiting regime is plausible for a low-direct-cost post hoc correction, although validation, integration, regulatory, and monitoring costs need not be negligible in other deployments. Low acquisition cost should therefore not be mistaken for low deployment consequence.

### Absolute Scale Cancels, but Relative Translation Can Change the Decision

Under *r*=1 and *K*/*M* approaching 0, uncertainty in a common strictly positive multiplier cannot alter strategy ordering because multiplication by a positive scalar is order-preserving. This is an algebraic property of the specified common-*M* model, not evidence that benefit and harm necessarily have equal downstream consequences.

The implemented base case is effectively in the negligible-cost limit: *K*/*M* is 4.3×10^–6^. Varying the absolute monetary value per averted event over the modeled range therefore changes the scale of net benefit but not the base-case deployment threshold to three decimals.

### The Threshold Is Insensitive to the Economic Parameters and Sensitive to the Classification Rule

Across one-way, probabilistic, horizon or discount, exchange-rate, and European cost sensitivities, the deployment threshold remained 0.50 at two decimals. One-way variation of the illustrative parameters moved it by 2.7-5.8×10^–5^, the 50,000-draw analysis by 2.8×10^–4^, horizon or discount by 6.4×10^–5^, and exchange rate by 2×10^–7^. Value exposed ranged from €38.3 million to €1131.8 million (base €344.0 million). These parameters set absolute scale and affect the threshold only through *K*/*M*=4.3×10^–6^ (Multimedia Appendix 7).

The monetary figures are scenario quantities of order-of-magnitude accuracy; the decision rule does not materially inherit that uncertainty.

The Norwegian adverse-event cost (€11,494) exceeds the European comparators: means of €5208 and €5974 with reported values €514-€27,380 [20], and approximately €6000-€10,000 per hospitalization [19]. It is a bed-day rate multiplied by excess stay rather than total admission cost. Substitution across these ranges leaves the threshold at 0.50; at €514, it is 0.5034.

Boundary choice mattered more. Sweeping ±0.05 in mean ΔMRR@10 produced thresholds 0.58, 0.50, 0.43, and 0.35; grouping was stable from –0.0157 to +0.0369. E5-Mistral-7B-ablation had an effect-to-dispersion ratio of 0.08 versus 0.30-1.39 for every other configuration, about 3.8-fold below the next lowest. The boundary range, 0.35-0.58, was comparable to but narrower than the regularization range, 0.32-0.70.

Reassigning that boundary configuration raised 6-condition specificity from 0.9362 to 0.9962 and lowered the screening requirement from 0.0712 to 0.0066, an approximately 11-fold change against a 17-fold change in the false-positive rate. The requirement therefore tracks one minus specificity and remains conditional on deployment-specific screening accuracy.

Relative translation is different. With the response-defined base-case effects and negligible cost, the threshold is 0.80 when *r*=0.25, 0.67 when *r*=0.5, 0.50 at *r*=1, 0.34 at *r*=2, and 0.20 at *r*=4. These are structural values holding the measured effects fixed; all inherit uncertainty in the underlying measured effect estimates, for which no deployment-population sampling interval is assigned. The informative quantity is therefore the direction and magnitude of movement rather than the apparent precision of any individual threshold. The assumption that technical benefit and harm map symmetrically to clinical and economic value is therefore decision-relevant and is not inferred from the retrieval data.

Within the common-*M* base case, the clinical and economic chain determines the size of the stakes rather than strategy ordering. Once benefit and harm are allowed different translation multipliers, their ratio becomes part of the deployment condition.

### The Literature Cannot Establish Whether the Condition Is Met

Because the publication frame does not identify an empirical distribution for response-defined prevalence, no population net-benefit estimate is presented as a primary result. The economic scenario is used to demonstrate that implementation cost is negligible, relative to the value exposed to the intervention, not to estimate population burden.

The analytic deployment threshold does not require a probability distribution over prevalence. Under the identification ladder, whose rungs are classification assumptions linking implementation characteristics to response rather than measurements of response, the frame’s proxy-based affected fraction is compared with a base-case common-*M* threshold of 0.50 (Table 1).

**Table 1:** Identification of the affected fraction within the published-system frame under 4 classification assumptions.

Classification assumption	Proxy-based interval	Crosses common-*M* threshold 0.50?
None	0-1	Yes—frame uninformative
Contrastive training implies nonmembership (self-hosted)	0.018-0.564	Yes—indeterminate
Vendor claims accepted for commercial interfaces	0.018-0.073	No
As above, excluding 3 nondense retrievers	0.019-0.077	No

Coding decisions for all 55 systems, the evidence behind each, the coding definitions, and their classification under every rung are given in Multimedia Appendix 8. With no classification assumption, the frame is uninformative about response-defined prevalence. Under the second rung, the proxy interval 0.018-0.564 crosses the 0.50 threshold and therefore remains decision-indeterminate. Only the stronger vendor-claim assumptions place the proxy interval wholly below the threshold; these remain classification assumptions rather than measurements of response and are not used to claim that universal deployment is harmful.

The publication frame therefore cannot establish whether universal deployment meets the threshold: this is an identification failure, not evidence that universal deployment is harmful. Enlarging the current literature would not resolve it unless representation-level deployment characteristics are reported.

No strategy-preference percentage is reported because it would be determined by an illustrative prevalence prior rather than observed deployment data.

Response-defined grouping also changes across regularization values. Recomputing the groups from the measured sign at each of the 6 full-corpus epsilon values gives zero-cost thresholds ranging from 0.316 to 0.699; the number of benefited configurations ranges from 4 to 7. Thus, regularization can change both effect magnitude and response-group membership. Cross-validated effects are not used for this sensitivity because the protocol is biased in favor of the intervention as described in the Limitations section.

### Screening Trades Evidence Breadth Against the Prior Requirement

Screen-and-treat is not unconditionally preferable. At 4 conditions, *S*=€1600 and *T*=€1468, giving *s*=4.7×10^–6^ and *t*=4.3×10^–6^; total strategy cost at the resulting threshold is about €1801. The exact expression is used in Table 2.

**Table 2:** Minimum prior probability at which local screening has positive expected value, by cross-condition evidence breadth.

Independent conditions scored	Sensitivity	Specificity	Minimum prior probability required (exact)	Versus universal requirement 0.50
1	0.687	0.819	0.2101	2.4× lower
2	0.742	0.885	0.1359	3.7× lower
3	0.777	0.912	0.1025	4.9× lower
4	0.804	0.925	0.0858	5.9× lower
5	0.825	0.932	0.0767	6.6× lower
6	0.843	0.936	0.0712	7.1× lower

For population policy, the threshold depends on unidentified prevalence *p*; for a site, it uses the prior probability of benefit. Table 2 therefore reports the minimum prior probability at which a screen of given accuracy has positive expected value.

Table 2 uses the exact expression including screening cost. The derived specificity is sensitive to E5-Mistral-7B-ablation, whose mean effect is near 0 (–0.016) and between-condition SD is largest (0.200). Assigning it to the harmed group lowers 6-condition specificity from 0.996 to 0.936 and raises the requirement from about 0.007 to 0.071. The response-defined grouping is retained, but this column is conditional on that boundary assignment.

Screening reduces the common-*M* prior requirement from 0.50 to 0.0858 at 4 conditions and 0.0712 at 6; the result is conditional on the site prior and benchmark-derived accuracy rather than a universal rule (Figure 2).

**Figure 2:**
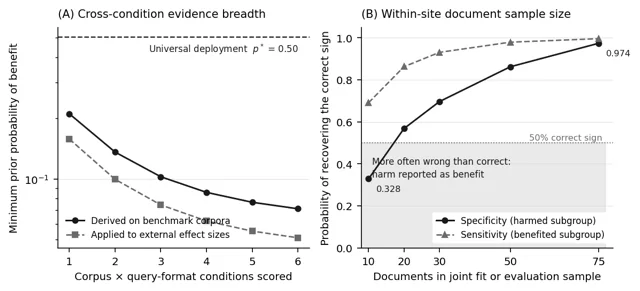
Two design axes for local screening. (A) Cross-condition evidence breadth: minimum prior probability at which screening has positive expected value, by the number of corpus-by-query-format conditions scored, derived on the benchmark corpora and applied to the external effect sizes. The horizontal line marks the universal-deployment threshold. (B) Joint fit-and-evaluation document sample: probability of recovering the correct sign when the same n documents are used both to fit the transform and to evaluate retrieval. Below 50%, the screen is more often wrong than correct, reporting harm as benefit. The panels vary different design axes; panel (B) does not identify separate universal minima for transform-fitting and evaluation sample sizes.

Higher site priors can support fewer scored conditions, whereas lower priors require greater specificity. The publication frame supplies neither a transportable prevalence nor a defensible site-specific prior.

Condition count and document count are separate design axes; the resampling analysis below evaluates the joint fit-and-evaluation document sample rather than a universal minimum number of conditions.

Derived sensitivity is 0.804 at 4 conditions after response-defined regrouping, so a screen can still miss systems that would benefit. False negatives forgo benefit; false positives expose harmed systems to the intervention, making specificity the more safety-critical operating characteristic in this worked case.

### Decision-Relevant Uncertainty Is Local and Asymmetric

The analysis does not assign a monetary value to perfect information about deployment prevalence because no empirical probability distribution for that prevalence is identified. Instead, uncertainty is separated by whether it can alter the decision (Table 3).

**Table 3:** Structural sensitivity of the universal-deployment threshold to relative benefit-versus-harm value translation.

*r*=*M*_ben/*M*_harm	Relative benefit translation	Zero-cost threshold	Interpretation
0.25	Benefit valued at 1/4 of harm	0.80	Much higher prevalence required
0.50	Benefit valued at 1/2 of harm	0.67	Higher prevalence required
1.00	Common-*M* base case	0.50	Reference
2.00	Benefit valued at 2× harm	0.34	Lower prevalence required
4.00	Benefit valued at 4× harm	0.20	Much lower prevalence required

Absolute common-scale parameters determine monetary magnitude but cannot change ordering in the demonstrated negligible-cost, *r*=1 limit. By contrast, response prevalence, benefit and harm magnitudes, and the relative translation ratio *r* are state variables that can move the threshold. Screening sensitivity, specificity, and measurement intensity are design variables that can be changed locally.

These classes call for different responses. Uncertainty in local response should trigger measurement; uncertainty in screening performance should trigger engineering or validation of the screening design; and uncertainty in *r* requires clinical evidence linking technical retrieval changes to downstream consequences. A large absolute economic multiplier alone is not a research priority for the adoption decision because it scales benefit and harm together in the common-*M* base case.

This distinction avoids presenting prior-driven population research priorities as if they were empirical findings. Standard value-of-information methods remain appropriate once a defensible distribution for deployment prevalence and downstream translation exists, but the present publication frame does not supply one.

Once a site has measured its own response with adequate accuracy, population prevalence no longer determines whether the correction is applied at that site. Population prevalence remains relevant to policies about whether broad screening programs are worthwhile; the site-level adoption decision depends on the local measurement and its error characteristics.

### The Benchmark-Derived Requirement Is Conservative for the Institutional Effect Sizes

The nominal mechanistic 2-tier pattern replicated without exception in the primary section analysis (Figure 3; complete cell-level matrix in Multimedia Appendix 1): across 52 cells—13 configurations, 2 institutions, and 2 query formats—every nominal harm-tier configuration was harmed, and every nominal benefit-tier configuration was helped. Response-defined transport from the benchmark is slightly different: E5-Mistral-7B-ablation is benchmark-harmed but benefits in all 4 institutional section conditions, so benchmark response-group assignment is retained in 48 of 52 cells. This discordance is evidence that response is not a fixed model label (Table 4).

**Figure 3:**
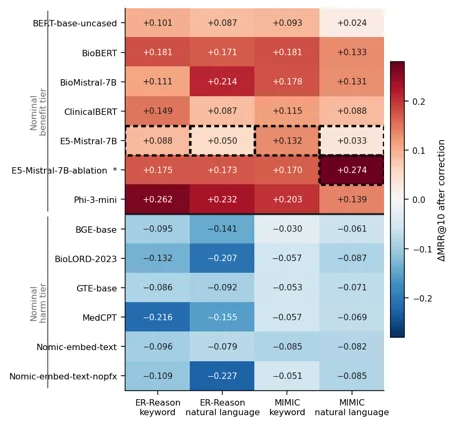
Measured response across deployment conditions. Cells give ΔMRR@10 after correction for each of 13 configurations in 4 corpus-by-query-format conditions, under the primary section-level analysis. All 52 cells agree with their nominal subgroup assignment. Dashed outlines mark the 5 cells whose assignment changes under uniform 512-token whole-note encoding, where 47 of 52 are retained, including all 24 nominal-harm-tier cells; 4 of the 5 changes involve E5-Mistral-7B. MIMIC: Medical Information Mart for Intensive Care; MRR: mean reciprocal rank. * Nominal benefit tier but harmed on the benchmark corpora (mean −0.016); benchmark-response agreement is 48 of 52.

**Table 4:** Measured effect by corpus and query format in the transport evaluation.

Corpus and format		Nominal harm-tier (n=6)			Nominal benefit-tier (n=7)			Local threshold
		Mean (SD)	Range	Mean (SD)		Range		
ER-Reason								
	Keyword	–0.122 (0.049)	–0.216 to –0.086	+0.152 (0.060)		+0.088 to +0.262	0.445	
	Natural language	–0.150 (0.060)	–0.227 to –0.079	+0.145 (0.070)		+0.050 to +0.232	0.509	
MIMIC-IV-Note^a^								
	Keyword	–0.056 (0.018)	–0.085 to –0.030	+0.153 (0.040)		+0.093 to +0.203	0.266	
	Natural language	–0.076 (0.010)	–0.087 to –0.061	+0.117 (0.084)		+0.024 to +0.274	0.392	

^a^MIMIC: Medical Information Mart for Intensive Care.

Baselines separate on the same axis: nominal harm-tier configurations retrieve at 0.494 to 0.859 before whitening, versus 0.050 to 0.450 in the nominal benefit tier. The harmed configurations are those already retrieving well, consistent with the geometric account in which whitening disrupts calibrated geometry and rescues collapsed geometry.

Applying the response-defined screening curve derived on the benchmark corpora, unchanged, to the observed institutional benefit and harm magnitudes gives lower prior-probability requirements than on the benchmark at every evidence breadth. For this calculation, institutional benefit and harm magnitudes were defined by the sign measured in the institutional conditions (+0.142 and –0.101), while sensitivity and specificity remained those derived from the benchmark response groups (Table 5).

**Table 5:** Minimum prior-probability requirement by cross-condition evidence breadth under benchmark-derived and external effect sizes.

Independent conditions scored	Requirement, response-defined benchmark	Requirement, institutional effect magnitudes
1	0.2101	0.1576
2	0.1359	0.0996
3	0.1025	0.0744
4	0.0858	0.0619
5	0.0767	0.0552
6	0.0712	0.0511

The curve therefore remains conservative for the 2 institutional corpora examined when applied to their measured positive and negative effect magnitudes. This is a bounded transport result, not proof that benchmark-derived sensitivity and specificity are universal across sites.

Pooling all 10 observed conditions changes E5-Mistral-7B-ablation back to the benefited response group and yields 7 benefited and 6 harmed configurations. The pooled universal common-*M* threshold is 0.50 after rounding, descriptively similar to the 6-condition benchmark estimate; no inferential comparison is made because neither panel represents a probability sample of deployments. Single-condition sensitivity is 0.765 and specificity 0.919, and the pooled 4-condition screening requirement is 0.0032. These pooled estimates are descriptive because the same external conditions also serve as transport evidence.

Between-condition variability was lower on both institutional corpora than on the benchmark set for all 13 configurations (median SD 0.047 vs 0.106). The institutional effects are also larger on the positive side (+0.142), while the negative magnitude is similar in the nominal-tier comparison, which contributes to the lower external screening requirements. Neither observation guarantees performance at an unmeasured site.

### Observed Screen Accuracy Diverges From the Derived Curve at Small Samples

The benchmark screening curve derives sensitivity and specificity from between-condition variability. A deploying site instead samples documents within one corpus, so its error rate is governed by a different quantity; the derived curve is a proxy rather than a direct measurement.

This was measured on both external corpora: for each configuration, 500 subsamples of *n* documents were drawn without replacement, whitening was refitted, retrieval rescored, and sign agreement with the full-sample effect recorded. Sensitivity and specificity were pooled across the benefited and harmed configurations; per-configuration results are in Multimedia Appendix 2.

Wilson intervals in Table 6 quantify Monte Carlo precision of the resampling agreement rate, not transport uncertainty. The overlapping draws come from 2 fixed 100-document corpora rather than independent deployment environments, so the point estimates carry the finding, and the intervals do not describe between-site spread.

**Table 6:** Empirical screen accuracy by joint fit-and-evaluation document sample size.

Documents in joint fit or evaluation sample	Sensitivity (95% CI)	Specificity (95% CI)	Interpretation
10	0.691 (0.683-0.698)	0.328 (0.320-0.337)	Specificity inverted
20	0.862 (0.856-0.868)	0.568 (0.559-0.576)	Low specificity
30	0.930 (0.926-0.934)	0.696 (0.688-0.704)	False positives remain common
50	0.979 (0.976-0.981)	0.862 (0.855-0.868)	Improving
75	0.995 (0.994-0.996)	0.974 (0.971-0.976)	High specificity in these corpora

Small samples can be directionally misleading: at 10 documents, specificity is 0.328, so harmed configurations are labeled beneficial in roughly two-thirds of resamples across both corpora.

A likely mechanism is covariance rank deficiency: with 10 documents in 768 dimensions, covariance rank is 9, and most directions are ridge-controlled. The experiment does not separate sample needs for transform estimation from those for estimating ΔMRR@10.

The 1 of 55 publication-frame count is not entered into the deployment model because it is neither an estimate nor a bound on response prevalence.

At 75 documents, specificity is 0.974; with institutional effect magnitudes, the corresponding negligible-cost prior requirement is about 0.018. Because document count does not determine condition count or fixed screening cost, and the same documents fit and evaluate the transform, this is not a universal 75-document floor; separate *n*_fit and *n*_eval experiments are required.

The 2 design questions are therefore distinct: how much cross-condition evidence a prior requires, and whether local document sampling estimates the sign reliably. Neither substitutes for the other.

### Deployment-Time Configuration Changes the Sign of the Effect

The panel contains 2 pairs that share a checkpoint and differ only in choices a deploying team makes at implementation, so the effect of those choices can be read directly.

E5-Mistral-7B and its ablation load the same weights and differ in pooling (final token vs mean) and in whether the documented instruction prefix is applied. Under whole-note encoding, the 2 have opposite signs in 3 of 4 conditions: the standard configuration is harmed (–0.140 to –0.098) while the ablation benefits (+0.045 to +0.259). The same checkpoint, deployed 2 ways, falls in different response groups.

Nomic-embed-text and its no-prefix variant isolate the prefix choice alone. Sign was identical in all 8 conditions, but magnitude differed by up to 2.9-fold (–0.079 vs –0.227 on ER-Reason natural-language queries). Prefix handling altered how much was lost, not whether anything was.

These are not model properties. Pooling, prefix handling, and document segmentation are configured during deployment, are not fixed by the checkpoint, and here determined whether the correction helped or harmed. The E5 pair cannot decompose pooling from prefix, since both differ; the Nomic pair shows prefix alone did not reverse sign, which is suggestive rather than decisive.

### Subgroup Membership Is Length-Dependent for One Configuration

Section extracts are primary because model context windows vary with nominal tier: all 6 nominal harm-tier configurations are BERT-scale at 512 tokens, whereas decoder configurations in the nominal benefit tier accept at least 2048. Native whole-note encoding would therefore expose tiers to systematically different text lengths.

That asymmetry can be removed by capping every configuration at 512 tokens so all encode identical text. Under that constraint, all 6 nominal harm-tier configurations remain harmed in all 4 conditions, but the nominal benefit tier is not invariant (Table 7).

**Table 7:** Effect sizes and thresholds under section-level and matched-length whole-note encoding.

Corpus and format			Nominal harm (section; n=6), mean (SD)		Nominal harm (whole; n=6), mean (SD)		Nominal benefit (section; n=7), mean (SD)		Nominal benefit (whole; n=7), mean (SD)		p* section		p* whole
ER-Reason													
	Keyword	–0.122 (0.049)		–0.104 (0.052)		+0.152 (0.060)		+0.101 (0.126)		0.445		0.509	
	Natural language	–0.150 (0.060)		–0.130 (0.060)		+0.145 (0.070)		+0.149 (0.148)		0.509		0.465	
MIMIC-IV-Note^a^													
	Keyword	–0.056 (0.018)		–0.118 (0.053)		+0.153 (0.040)		+0.058 (0.086)		0.266		0.673	
	Natural language	–0.076 (0.010)		–0.188 (0.056)		+0.117 (0.084)		+0.033 (0.088)		0.392		0.850	

^a^MIMIC: Medical Information Mart for Intensive Care.

E5-Mistral-7B drives most of the difference: it benefits on sections (+0.076 mean) and is harmed on whole notes (–0.122) in all 5 conditions. In total, 5 of the other 6 nominal benefit-tier configurations remain positive in all 4 whole-note conditions; the ablation is marginally negative in one cell. Excluding E5-Mistral, nominal benefit-tier means remain positive throughout.

E5-Mistral’s unwhitened retrieval rises about 5-fold with longer text (ER-Reason: 0.100->0.571; MIMIC-IV-Note: 0.072->0.516), while the ablation changes little. As the only final-token-pooling configuration, its baseline geometry becomes well calibrated with longer input, and whitening becomes harmful.

The same checkpoint therefore occupies different response groups at different document lengths. Subgroup membership depends on the deployed representation and document regime, not model identity alone.

### The Query-Generation Substitution Is Unlikely to Explain the External Result

Queries for the external corpora were generated locally rather than with the commercial model used in the derivation, for the data-governance reason given in the Methods section. Whether that substitution produced the external result can be tested on a public corpus for which both query sets exist. The identical panel was run twice on the derivation study’s own 100-document MTSamples sample, once with its published queries and once with queries generated by the local pipeline, with documents, models, and every other setting held constant.

Per-comparison results and the full query-generation protocol are given in Multimedia Appendix 3. Configuration rankings agreed closely: Kendall τ is 0.769 for keyword queries and 0.795 for natural language (*P*<.001; Spearman 0.912 and 0.923), inside the 0.59-0.90 range previously reported for variation across query derivations [1]. Subgroup assignment agreed in 25 of 26 configuration-by-format comparisons. The single disagreement was a boundary case: one configuration measured +0.0003 under the published queries and –0.0070 under the local ones, 3 ten-thousandths from 0 in the reference condition.

One configuration is assigned to the harmed subgroup on this corpus under both query sets despite belonging to the benefited subgroup in the derivation panel and on both external corpora—ClinicalBERT, at –0.032 with the published queries and –0.040 with the local ones, on keyword queries only. Since both query sets agree, this is not an artifact of the substitution. It is a fourth instance of assignment depending on something other than the model: here on the corpus-by-query-format condition rather than on document length or training procedure. The design observes one measurement per condition and cannot separate corpus from query format or their interaction.

### The Published Code-Search Subgroup Structure Does Not Reproduce

The framework applies only where an intervention’s effect changes sign across response-defined subgroups. Because that antecedent is empirical, it was tested against a published semantic code-search experiment reporting the same subgroup structure in an unrelated domain [13], replicated from released code and data (Multimedia Appendix 9). Baseline retrieval reproduced closely: across the 18 primary replication cells, the baseline metric matched published values to within 0.001. The published subgroup structure did not reproduce. At zero regularization, 3 of 4 configurations had positive mean effects, and CodeT5+ was negative (mean ΔMRR@10 –0.0314; Multimedia Appendix 9), so response-defined groups were present, but their membership differed from the published mechanistic account: the contrastively fine-tuned CodeBERT, which that account places in the harmed group, had a positive mean effect (+0.0285). Its fine-tuned checkpoint was not released, so it was fine-tuned here with the authors’ script at their published settings; the resulting model exceeded the published baseline in all 6 languages (Multimedia Appendix 9).

Regularization did not restore a negative effect for that configuration: its mean effect was positive at all 6 values. CodeT5+ was negative at regularization 0, 10^–4^ and 10^–3^ and positive at 10^–2^ and above, so sign-changing heterogeneity was present in code search only at low regularization. In the clinical panel, the harmed group was nonempty at every 1 of the 6 values, comprising 6 to 9 of the 13 configurations. Whether an intervention is sign-changing can therefore depend on its own hyperparameter, which neither model identity nor a published subgroup assignment conveys.

Two consequences follow for the clinical result. The antecedent of the framework is a condition to be checked in each setting rather than a property of the correction, and because the fine-tuned checkpoint was not released, it had to be reimplemented, so the failure to reproduce the published mechanistic grouping is evidence that subgroup membership need not survive independent reimplementation.

## Discussion

### Principal Findings

The intervention evaluated is the decision to apply a geometric correction in a particular deployment, not whitening as a universal prescription. Deployment choices—representation extraction, prefix, segmentation, and query formation—can change the measured effect without retraining, so the operative object is the locally produced representation.

Model identity and published descriptors did not reliably recover the response state. Evidence came from incomplete extraction reporting, an independent reimplementation, E5-Mistral’s length-dependent sign change, ClinicalBERT’s corpus-query dependence, and E5-Mistral-7B-ablation’s benchmark-to-institution sign change.

Universal deployment is justified only above a response-prevalence threshold; yet, the publication frame cannot identify that prevalence. Local measurement converts this population-identification problem into a site-specific decision. Under common *M*, absolute scale changes the stakes; asymmetric benefit-harm translation changes the threshold.

Direct implementation cost is tiny, relative to modeled value exposed, so low acquisition cost does not protect against transferred harm. The economic scenario illustrates scale rather than population burden.

Local screening also requires an adequate sampling design. In the tested joint fit-and-evaluation protocol, 10 documents produced specificity below 0.5; because *n*_fit and *n*_eval were not separated, the study warns against undersampled auditing but does not establish a universal 75-document minimum.

### Comparison With Prior Work

Value-of-information analysis is used in health economic evaluation [28], but it requires probability distributions that represent uncertainty rather than convenience. Here, the load-bearing population quantity—response-defined deployment prevalence—is not empirically identified. The analysis therefore reports structural decision relevance instead of a prior-dependent monetary expected value of perfect information: absolute common scale cancels in the negligible-cost base case, relative benefit-harm translation does not, and local screening accuracy is a design property rather than a population fact.

Across the systematic reviews identified [5-7], no economic evaluation was found addressing a component-level correction to a clinical AI system rather than a patient-facing AI intervention. The analytic form is not specific to this correction: any intervention whose effect changes sign across response-defined subgroups admits the same threshold.

Cross-site evidence is easily conflated with this question. Evidence that relative model rankings transfer between institutions concerns which model to select; the deployment condition concerns whether applying an intervention to an already-selected configuration has positive net benefit. The present results show that response-defined subgroup assignment can change with deployment-time configuration, document regime, and corpus-query context. Evidence supporting transport of model selection therefore does not by itself establish transport of intervention response. Local validation is not a single requirement; the evidence required depends on the decision being transferred.

### A Boundary Condition From an Unrelated Domain

The deployment threshold applies only when an intervention has sign-changing effects; that antecedent is empirical and need not hold universally.

Sign-changing heterogeneity was present in code search only below a regularization value at which it vanished, while the clinical harmed group persisted across the full sweep (see Results section), so neither a model label nor a published subgroup assignment can be assumed to substitute for local measurement.

Within clinical text, nominal mechanistic tier remained highly predictive on the institutional section corpora (52/52 cells), but benchmark response membership transported in 48 of 52 because E5-Mistral-7B-ablation changed sign. This combination supports local validation without implying that every deployment differs.

### The Published Record Does Not Describe the Deployed Configuration

Deployment-time choices that can alter responses are poorly represented in published descriptions. None of the 55 systems reported retrieval pooling as such, and 27 used commercial interfaces with undisclosed geometry. The frame assessed pooling and extraction only, so it does not establish reporting gaps for every configuration element.

No system in the frame had been evaluated under the intervention, and only 3 named a checkpoint matching one measured here; even for those, extraction was unreported. The measured effects therefore cannot be transferred simply from checkpoint identity.

### Strengths and Limitations

The algebraic threshold is general to sign-changing response-defined effects, but the common-*M* form assumes equal downstream translation of benefit and harm; the *r* sensitivity makes this explicit. The empirical demonstration is limited to 1 intervention class, English text, and 5 corpora. Whether these empirical findings generalize to other clinical-AI interventions or health systems remains currently untested.

No transportable deployment prevalence is estimated because the frame samples publications rather than deployments, and response membership is often unobservable. Proxy intervals depend on classification assumptions and are not treated as response measurements.

The companion study’s cross-validated effects are not the base case because its transform is fitted on 80 documents, but ranking uses the full 100-document candidate set, so held-out targets escape fitting, while most competitors do not. All 13 cross-validated effects exceed full-corpus effects, and the gap correlates strongly with baseline retrieval. Full-corpus effects are therefore used instead; the issue has been communicated separately to the companion journal.

Nominal mechanistic tier and measured response are not identical; the base-case decision model therefore uses response-defined grouping rather than nominal labels.

External transport is bounded: nominal tier matched all 52 institutional section cells, whereas benchmark response membership matched 48 of 52 because one configuration changed sign across corpora. Ten conditions were observed across 5 corpora, including 2 US institutions; prospective deployment, non-US systems, other languages, and unrestricted whole-document retrieval remain untested.

Three parameters in the absolute multiplier *M* are illustrative, so absolute event counts remain scenario-derived. Equality of benefit and harm translation is also unestablished and is addressed only through structural *r* sensitivity.

Screening accuracy is locally contingent; the benchmark-derived curve was conservative for the 2 institutional corpora examined but should not be assumed transportable to materially different documentation or workflows.

E5-Mistral-7B-ablation remains an anomalous boundary configuration because pooling and instruction prefix were changed together and cannot be decomposed; it is retained for comparability rather than excluded.

Section extraction is a scope condition as well as a confound control. Whole-note sensitivity showed that document length can alter response, so section-level findings should not be assumed to hold at other input lengths; native-limit behavior remains unmeasured.

The resampling experiment concerns the joint fit-and-evaluation design on 2 fixed 100-document corpora. Larger subsamples increasingly agree with the full-sample reference by construction, and one configuration remains an exception at 75 documents on MIMIC-IV-Note. Because *n*_fit and *n*_eval were coupled, the experiment cannot determine separate minimum sample sizes for covariance estimation and effect estimation; larger independent corpora would be needed for that factorial analysis.

External queries used a different generator for data-governance reasons. Paired testing on MTSamples showed strong rank agreement and identical subgroup assignment in 25 of 26 comparisons, but no paired comparison was possible on the external corpora, so generator-related uncertainty is bounded rather than eliminated.

The threshold carries no statistical uncertainty bounds. Because the panel is purposive and the response groups are defined from the same effects that determine their means, 0.50 is a point estimate conditional on that panel and classification rule and should not be generalized as though it had a sampling distribution. The 2 sweeps bound it structurally instead, at 0.32 to 0.70 across regularization and 0.35 to 0.58 across the classification boundary—both far wider than anything the economic parameters contribute.

The analysis addresses representational heterogeneity, not fairness. Differential performance or downstream effects across demographic or priority populations were not assessed.

The technical-to-clinical mapping is the model’s weakest link. MRR records the rank of the first relevant document, whereas decisions may require multiple details, so a linear multiplier is only a simplification. A severely degraded baseline such as ClinicalBERT (0.119-0.186) may remain clinically inadequate despite proportional gain, making model replacement more rational than correction. The framework compares correction with no action, not substitution. The modeled loss of 0.0015 QALY is about half a day of full health; a larger penalty would favor active strategies, so this value is conservative rather than an estimate of true retrieval-error severity.

No action is assigned zero strategy cost, although a degraded retrieval pipeline may consume clinician search time. Adding such opportunity cost to no action would reduce incremental active-strategy cost and therefore favor screening; its omission is conservative. It was not quantified because no empirical estimate was available, and any threshold shift would depend on the value assigned.

Universal deployment is an idealized comparator: system-wide application of an unvalidated correction without local testing. It represents action without local evidence rather than a realistic governance strategy; the issue is comparator realism, not a missing cost term.

Query generation introduces 2 limitations. Metadata came from the first 6000 characters of the full note and may therefore extend beyond the indexed section; queries were generated from metadata alone, limiting but not excluding lexical overlap, whose effect may differ between dense and lexical retrieval. Output was truncated at the first non-ASCII character to prevent language switching, a rule that could remove clinically meaningful Greek letters. Cleaning was applied inline during generation, and raw outputs were not retained, so the frequency of truncation events and the content removed cannot be reconstructed; generation used a nonzero temperature without a fixed seed, so rerunning would not reproduce the original outputs. These rules were constant within conditions but may affect models differently.

The unit of analysis is a retrieval system, not a patient: no cohort is enrolled, and no individual outcomes are modeled. The study is therefore a decision-analytic evaluation of a system-level deployment rule rather than a patient-level economic evaluation. Reporting follows CHEERS 2022 and CHEERS-AI; some AI-specific training-data items cannot be answered for third-party checkpoints with undisclosed corpora.

Inputs are not contemporaneous: corpora span 2008-2019, excess stay is from 2015, willingness to pay from 2015 to 2016, unit cost from 2024, comparator costs from 2018 to 2023 tariffs, and checkpoints from 2022 to 2024. A common price year makes monetary values comparable, not contemporaneous. The 5-year horizon was therefore varied from 1 to 10 years; the threshold remained essentially unchanged because monetary inputs enter through the negligible cost ratio.

No health-economic analysis plan was prespecified. Parameter selection and scenario design were therefore post hoc, so selective parameterization and selective scenario testing remain potential sources of bias; the breadth of the sensitivity analyses and the publicly archived code and parameters improve transparency but do not substitute for prespecification.

### Conclusions

Whitening improved retrieval for some configurations and degraded it for others, and measured response was not a fixed property of model identity. E5-Mistral-7B-ablation changed from a negative benchmark mean to positive effects across the institutional section conditions, while E5-Mistral-7B changed sign with document length. The published record does not report enough deployment detail to infer these states, and no operational system in the 55-system frame was evaluated under the correction.

The decision framework was evaluated across benchmark, institutional, document-length, query-generation, and resampling analyses. The common-*M* threshold is 0.50, but asymmetric downstream translation can move it from 0.20 to 0.80 over the structural *r* sensitivity. Local screening can lower the required prior probability of benefit, but its reliability depends on measurement design, and the present resampling does not establish a universal document-count floor.

The claim is therefore not that this correction fails, nor that every clinical AI system requires a particular form of retraining. It is that deployment validity cannot be inferred safely from a global model label when the response can change with local configuration and context. In the setting examined, because the response-defining state could not be established reliably from model identity or available external evidence, local validation was required before applying the sign-changing intervention; where the measured state is unsuitable, local action—modification, adaptation, withholding the intervention, or further evidence collection—is necessary rather than plug-and-play transfer.

## Data Availability

The datasets generated or analyzed during this study are available in the GitHub repository [29], archived as a frozen Zenodo release [30]. These comprise the decision model, the screening derivation, the local resampling procedure, the transport evaluation, the query-generation pipeline, the code-search replication, the system-extraction frame, and all aggregate analysis outputs. The underlying clinical corpora are PhysioNet credentialed-access resources governed by data use agreements; no patient-level data are redistributed.

## Appendix

Multimedia Appendix 1 Complete transport matrix: baseline, corrected, and delta mean reciprocal rank @10 by configuration, corpus, query format, document variant, and subgroup assignment.

Multimedia Appendix 2 Local-screen resampling by configuration, corpus, query format, sample size, sign agreement, and Wilson intervals.

Multimedia Appendix 3 Query-generation robustness under published and local queries, with rankings, subgroup assignment, and protocol.

Multimedia Appendix 4 CHEERS 2022 checklist.

Multimedia Appendix 5 CHEERS-AI checklist.

Multimedia Appendix 6 Decision-model parameters, sources, regularization sweep, and per-condition effects.

Multimedia Appendix 7 Economic, horizon, discount-rate, exchange-rate, adverse-event-cost, probabilistic, and classification-boundary sensitivity analyses.

Multimedia Appendix 8 System extraction frame, coding definitions, evidence, and 4 classification assumptions for 55 clinical retrieval-augmented generation systems.

Multimedia Appendix 9 Code-search replication: baseline, whitening effects, and regularization grid.

## Abbreviations

ASCII: American Standard Code for Information Interchange
CHEERS: Consolidated Health Economic Evaluation Reporting Standards
CHEERS-AI: Consolidated Health Economic Evaluation Reporting Standards for Interventions That Use Artificial Intelligence
MIMIC: Medical Information Mart for Intensive Care
MRR: mean reciprocal rank
QALY: quality-adjusted life year
RAG: retrieval-augmented generation
UCSF: University of California, San Francisco
ZCA: zero-phase component analysis
