# Supplementary material for: Deriving a Deployment Threshold for Benefit and Harm in Clinical AI: Multi-Institutional Decision-Analytic Modeling Study
Source: JMIR AI. 2026 Oct 23;5:e109863. doi: 10.2196/109863 (PMC13743961; doi:10.2196/109863)
Supplement: Supplementary file 1 [file ai_v5i1e109863_app1.docx]

**Corpus construction, section extraction, and the complete transport matrix**

*Baseline, corrected, and change in mean reciprocal rank at cutoff 10 for every configuration, corpus, query format, and document variant, with nominal and benchmark-response subgroup assignment.*

**Column definitions**

| **column** | **definition** |
| --- | --- |
| Corpus | er_reason (UCSF) or mimic_discharge (BIDMC) |
| Document variant | hpi = presenting-narrative section (primary analysis); full512 = whole note with all configurations capped at 512 tokens (sensitivity analysis) |
| Query format | nl = natural language; keyword = keyword |
| Configuration | embedding configuration as specified in the panel |
| Nominal tier | 1 = nominal harm tier; 2 = nominal benefit tier |
| Max tokens | sequence length used for that variant |
| Baseline MRR@10 | mean reciprocal rank at cutoff 10 before correction |
| Corrected MRR@10 | mean reciprocal rank at cutoff 10 after corpus-only whitening at epsilon = 1e-5 |
| dMRR@10 | corrected minus baseline |
| nominal subgroup | harmed or benefited, from the nominal tier |
| retains assignment | whether the measured sign matches the nominal tier |
| benchmark response group | harmed or benefited, from the sign of the configuration's mean effect on the derivation corpora; differs from nominal tier for E5-Mistral-7B-ablation |
| agrees with benchmark response group | whether the measured sign matches that group |

Nominal agreement is 52 of 52 on section-level text and 47 of 52 under whole-note encoding at matched context length. Benchmark-response agreement is 48 of 52 on section-level text; the difference is E5-Mistral-7B-ablation, which is harmed on the benchmark corpora and benefited in all four institutional conditions.

**Corpus construction**

Sources were ER-Reason v1.0.0, UCSF emergency-department provider notes, and MIMIC-IV-Note v2.2, BIDMC discharge summaries. Both are PhysioNet credentialed-access resources and note text is not redistributed here.

One hundred documents were sampled per corpus at random seed 42, without replacement, from documents of at least 400 characters. Sampling was unstratified because the six derivation conditions are unstratified, and any change in the screening curve could not otherwise be attributed to the corpora rather than to the sampling.

Median document length was 6,741 characters for ER-Reason and 9,774 characters for MIMIC-IV-Note, against approximately 400 tokens in the benchmark set.

**Section extraction**

The presenting narrative was identified by header. Extraction succeeded on 100 of 100 documents in both corpora, but the header vocabulary differs between them.

• MIMIC-IV-Note: "Brief Hospital Course" or "History of Present Illness".

• ER-Reason: "History of Present Illness" appears in only a small minority of the sampled notes, so matching required "HISTORY" with a whitespace boundary.

Median extract length was 694 characters for ER-Reason and 1,269 characters for MIMIC-IV-Note. Both are length-comparable to the benchmark corpora and are encoded whole by every configuration in the panel.

Seven of 100 ER-Reason extracts retain a leading form-field label. The label was retained as part of the source document text and was therefore present identically for all evaluated configurations. It affects document text only, never queries; its contribution to the document covariance on which the transform is fitted was not quantified.

The same clinical section, differently labelled in two corpora and requiring different extraction patterns, is a small instance of the paper's own argument that preprocessing does not transport without local inspection.

**Why a section extract is the primary analysis**

The panel's maximum sequence length is not uniform, and the non-uniformity aligns with nominal tier: all six nominal harm-tier configurations are BERT-scale at 512 tokens, while all four decoder configurations, all in the nominal benefit tier, accept 2,048 or 4,096. Encoding whole notes at native limits would give one group entire documents and the other a fifth of each, advantaging the benefit tier for reasons unrelated to whitening. The section extract is encoded whole by every configuration, so that asymmetry is absent. The whole-note sensitivity analysis caps all configurations at 512 tokens, which removes the asymmetry differently and is reported as full512.

Native-limit encoding was not run: it confounds context window with subgroup, and its result would not be interpretable.

**Table S1. Cell-level transport matrix**

| **Corpus** | **Document variant** | **Query format** | **Configuration** | **Nominal tier** | **Max tokens** | **Baseline MRR@10** | **Corrected MRR@10** | **dMRR@10** | **nominal subgroup** | **benchmark response group** | **agrees with benchmark response group** | **measured sign** | **retains assignment** |
| --- | --- | --- | --- | --- | --- | --- | --- | --- | --- | --- | --- | --- | --- |
| er_reason | full512 | keyword | BGE-base | 1 | 512 | 0.8004 | 0.7364 | -0.064 | harmed | harmed | yes | negative | yes |
| er_reason | full512 | keyword | BioLORD-2023 | 1 | 512 | 0.6796 | 0.5312 | -0.1484 | harmed | harmed | yes | negative | yes |
| er_reason | full512 | keyword | GTE-base | 1 | 512 | 0.8058 | 0.7397 | -0.0661 | harmed | harmed | yes | negative | yes |
| er_reason | full512 | keyword | MedCPT | 1 | 512 | 0.775 | 0.5892 | -0.1858 | harmed | harmed | yes | negative | yes |
| er_reason | full512 | keyword | Nomic-embed-text | 1 | 512 | 0.7837 | 0.7232 | -0.0605 | harmed | harmed | yes | negative | yes |
| er_reason | full512 | keyword | Nomic-embed-text-nopfx | 1 | 512 | 0.7853 | 0.686 | -0.0993 | harmed | harmed | yes | negative | yes |
| er_reason | full512 | keyword | BERT-base-uncased | 2 | 512 | 0.0765 | 0.1682 | 0.0916 | benefited | benefited | yes | positive | yes |
| er_reason | full512 | keyword | BioBERT | 2 | 512 | 0.1499 | 0.2763 | 0.1264 | benefited | benefited | yes | positive | yes |
| er_reason | full512 | keyword | BioMistral-7B | 2 | 512 | 0.1815 | 0.3329 | 0.1514 | benefited | benefited | yes | positive | yes |
| er_reason | full512 | keyword | ClinicalBERT | 2 | 512 | 0.1673 | 0.2476 | 0.0803 | benefited | benefited | yes | positive | yes |
| er_reason | full512 | keyword | E5-Mistral-7B | 2 | 512 | 0.5783 | 0.4387 | -0.1396 | benefited | benefited | no | negative | no |
| er_reason | full512 | keyword | E5-Mistral-7B-ablation | 2 | 512 | 0.5243 | 0.6355 | 0.1112 | benefited | harmed | no | positive | yes |
| er_reason | full512 | keyword | Phi-3-mini | 2 | 512 | 0.3068 | 0.5893 | 0.2824 | benefited | benefited | yes | positive | yes |
| er_reason | full512 | nl | BGE-base | 1 | 512 | 0.8497 | 0.7956 | -0.0541 | harmed | harmed | yes | negative | yes |
| er_reason | full512 | nl | BioLORD-2023 | 1 | 512 | 0.7901 | 0.6104 | -0.1796 | harmed | harmed | yes | negative | yes |
| er_reason | full512 | nl | GTE-base | 1 | 512 | 0.8595 | 0.7893 | -0.0702 | harmed | harmed | yes | negative | yes |
| er_reason | full512 | nl | MedCPT | 1 | 512 | 0.7652 | 0.5594 | -0.2058 | harmed | harmed | yes | negative | yes |
| er_reason | full512 | nl | Nomic-embed-text | 1 | 512 | 0.8808 | 0.7557 | -0.1252 | harmed | harmed | yes | negative | yes |
| er_reason | full512 | nl | Nomic-embed-text-nopfx | 1 | 512 | 0.8584 | 0.7162 | -0.1422 | harmed | harmed | yes | negative | yes |
| er_reason | full512 | nl | BERT-base-uncased | 2 | 512 | 0.0656 | 0.1509 | 0.0854 | benefited | benefited | yes | positive | yes |
| er_reason | full512 | nl | BioBERT | 2 | 512 | 0.0522 | 0.227 | 0.1748 | benefited | benefited | yes | positive | yes |
| er_reason | full512 | nl | BioMistral-7B | 2 | 512 | 0.1288 | 0.3329 | 0.2041 | benefited | benefited | yes | positive | yes |
| er_reason | full512 | nl | ClinicalBERT | 2 | 512 | 0.1862 | 0.2486 | 0.0624 | benefited | benefited | yes | positive | yes |
| er_reason | full512 | nl | E5-Mistral-7B | 2 | 512 | 0.5641 | 0.4659 | -0.0982 | benefited | benefited | no | negative | no |
| er_reason | full512 | nl | E5-Mistral-7B-ablation | 2 | 512 | 0.4545 | 0.7133 | 0.2589 | benefited | harmed | no | positive | yes |
| er_reason | full512 | nl | Phi-3-mini | 2 | 512 | 0.2549 | 0.6099 | 0.355 | benefited | benefited | yes | positive | yes |
| mimic_discharge | full512 | keyword | BGE-base | 1 | 512 | 0.7928 | 0.6622 | -0.1306 | harmed | harmed | yes | negative | yes |
| mimic_discharge | full512 | keyword | BioLORD-2023 | 1 | 512 | 0.6183 | 0.4431 | -0.1752 | harmed | harmed | yes | negative | yes |
| mimic_discharge | full512 | keyword | GTE-base | 1 | 512 | 0.826 | 0.7332 | -0.0929 | harmed | harmed | yes | negative | yes |
| mimic_discharge | full512 | keyword | MedCPT | 1 | 512 | 0.7758 | 0.6014 | -0.1744 | harmed | harmed | yes | negative | yes |
| mimic_discharge | full512 | keyword | Nomic-embed-text | 1 | 512 | 0.7446 | 0.7072 | -0.0375 | harmed | harmed | yes | negative | yes |
| mimic_discharge | full512 | keyword | Nomic-embed-text-nopfx | 1 | 512 | 0.7355 | 0.637 | -0.0985 | harmed | harmed | yes | negative | yes |
| mimic_discharge | full512 | keyword | BERT-base-uncased | 2 | 512 | 0.1182 | 0.2007 | 0.0826 | benefited | benefited | yes | positive | yes |
| mimic_discharge | full512 | keyword | BioBERT | 2 | 512 | 0.1732 | 0.2559 | 0.0827 | benefited | benefited | yes | positive | yes |
| mimic_discharge | full512 | keyword | BioMistral-7B | 2 | 512 | 0.2684 | 0.2985 | 0.0302 | benefited | benefited | yes | positive | yes |
| mimic_discharge | full512 | keyword | ClinicalBERT | 2 | 512 | 0.2714 | 0.3629 | 0.0915 | benefited | benefited | yes | positive | yes |
| mimic_discharge | full512 | keyword | E5-Mistral-7B | 2 | 512 | 0.5371 | 0.4305 | -0.1065 | benefited | benefited | no | negative | no |
| mimic_discharge | full512 | keyword | E5-Mistral-7B-ablation | 2 | 512 | 0.4671 | 0.512 | 0.0449 | benefited | harmed | no | positive | yes |
| mimic_discharge | full512 | keyword | Phi-3-mini | 2 | 512 | 0.3155 | 0.4927 | 0.1773 | benefited | benefited | yes | positive | yes |
| mimic_discharge | full512 | nl | BGE-base | 1 | 512 | 0.8715 | 0.6736 | -0.1979 | harmed | harmed | yes | negative | yes |
| mimic_discharge | full512 | nl | BioLORD-2023 | 1 | 512 | 0.7321 | 0.4644 | -0.2677 | harmed | harmed | yes | negative | yes |
| mimic_discharge | full512 | nl | GTE-base | 1 | 512 | 0.8737 | 0.7479 | -0.1258 | harmed | harmed | yes | negative | yes |
| mimic_discharge | full512 | nl | MedCPT | 1 | 512 | 0.6996 | 0.4858 | -0.2138 | harmed | harmed | yes | negative | yes |
| mimic_discharge | full512 | nl | Nomic-embed-text | 1 | 512 | 0.7972 | 0.6773 | -0.1199 | harmed | harmed | yes | negative | yes |
| mimic_discharge | full512 | nl | Nomic-embed-text-nopfx | 1 | 512 | 0.7952 | 0.5895 | -0.2057 | harmed | harmed | yes | negative | yes |
| mimic_discharge | full512 | nl | BERT-base-uncased | 2 | 512 | 0.092 | 0.1384 | 0.0464 | benefited | benefited | yes | positive | yes |
| mimic_discharge | full512 | nl | BioBERT | 2 | 512 | 0.0938 | 0.1714 | 0.0777 | benefited | benefited | yes | positive | yes |
| mimic_discharge | full512 | nl | BioMistral-7B | 2 | 512 | 0.1289 | 0.208 | 0.079 | benefited | benefited | yes | positive | yes |
| mimic_discharge | full512 | nl | ClinicalBERT | 2 | 512 | 0.1637 | 0.2561 | 0.0924 | benefited | benefited | yes | positive | yes |
| mimic_discharge | full512 | nl | E5-Mistral-7B | 2 | 512 | 0.4947 | 0.3516 | -0.1431 | benefited | benefited | no | negative | no |
| mimic_discharge | full512 | nl | E5-Mistral-7B-ablation | 2 | 512 | 0.4637 | 0.443 | -0.0207 | benefited | harmed | yes | negative | no |
| mimic_discharge | full512 | nl | Phi-3-mini | 2 | 512 | 0.2797 | 0.3816 | 0.102 | benefited | benefited | yes | positive | yes |
| er_reason | hpi | keyword | BGE-base | 1 | 512 | 0.7422 | 0.6469 | -0.0954 | harmed | harmed | yes | negative | yes |
| er_reason | hpi | keyword | BioLORD-2023 | 1 | 512 | 0.5553 | 0.4237 | -0.1315 | harmed | harmed | yes | negative | yes |
| er_reason | hpi | keyword | GTE-base | 1 | 512 | 0.7573 | 0.671 | -0.0863 | harmed | harmed | yes | negative | yes |
| er_reason | hpi | keyword | MedCPT | 1 | 512 | 0.6883 | 0.4721 | -0.2162 | harmed | harmed | yes | negative | yes |
| er_reason | hpi | keyword | Nomic-embed-text | 1 | 512 | 0.7361 | 0.6403 | -0.0958 | harmed | harmed | yes | negative | yes |
| er_reason | hpi | keyword | Nomic-embed-text-nopfx | 1 | 512 | 0.6726 | 0.5637 | -0.1089 | harmed | harmed | yes | negative | yes |
| er_reason | hpi | keyword | BERT-base-uncased | 2 | 512 | 0.119 | 0.2204 | 0.1014 | benefited | benefited | yes | positive | yes |
| er_reason | hpi | keyword | BioBERT | 2 | 512 | 0.1716 | 0.3522 | 0.1806 | benefited | benefited | yes | positive | yes |
| er_reason | hpi | keyword | BioMistral-7B | 2 | 2048 | 0.1477 | 0.259 | 0.1113 | benefited | benefited | yes | positive | yes |
| er_reason | hpi | keyword | ClinicalBERT | 2 | 512 | 0.1787 | 0.3282 | 0.1495 | benefited | benefited | yes | positive | yes |
| er_reason | hpi | keyword | E5-Mistral-7B | 2 | 4096 | 0.0996 | 0.1874 | 0.0877 | benefited | benefited | yes | positive | yes |
| er_reason | hpi | keyword | E5-Mistral-7B-ablation | 2 | 4096 | 0.3821 | 0.5567 | 0.1746 | benefited | harmed | no | positive | yes |
| er_reason | hpi | keyword | Phi-3-mini | 2 | 4096 | 0.2098 | 0.4714 | 0.2617 | benefited | benefited | yes | positive | yes |
| er_reason | hpi | nl | BGE-base | 1 | 512 | 0.8174 | 0.6765 | -0.141 | harmed | harmed | yes | negative | yes |
| er_reason | hpi | nl | BioLORD-2023 | 1 | 512 | 0.6635 | 0.4564 | -0.2071 | harmed | harmed | yes | negative | yes |
| er_reason | hpi | nl | GTE-base | 1 | 512 | 0.8109 | 0.7183 | -0.0925 | harmed | harmed | yes | negative | yes |
| er_reason | hpi | nl | MedCPT | 1 | 512 | 0.5796 | 0.4244 | -0.1552 | harmed | harmed | yes | negative | yes |
| er_reason | hpi | nl | Nomic-embed-text | 1 | 512 | 0.8062 | 0.7277 | -0.0785 | harmed | harmed | yes | negative | yes |
| er_reason | hpi | nl | Nomic-embed-text-nopfx | 1 | 512 | 0.7193 | 0.4922 | -0.227 | harmed | harmed | yes | negative | yes |
| er_reason | hpi | nl | BERT-base-uncased | 2 | 512 | 0.066 | 0.1526 | 0.0866 | benefited | benefited | yes | positive | yes |
| er_reason | hpi | nl | BioBERT | 2 | 512 | 0.0799 | 0.2507 | 0.1708 | benefited | benefited | yes | positive | yes |
| er_reason | hpi | nl | BioMistral-7B | 2 | 2048 | 0.1487 | 0.3628 | 0.2141 | benefited | benefited | yes | positive | yes |
| er_reason | hpi | nl | ClinicalBERT | 2 | 512 | 0.1752 | 0.2621 | 0.0869 | benefited | benefited | yes | positive | yes |
| er_reason | hpi | nl | E5-Mistral-7B | 2 | 4096 | 0.1009 | 0.1512 | 0.0504 | benefited | benefited | yes | positive | yes |
| er_reason | hpi | nl | E5-Mistral-7B-ablation | 2 | 4096 | 0.4497 | 0.6227 | 0.173 | benefited | harmed | no | positive | yes |
| er_reason | hpi | nl | Phi-3-mini | 2 | 4096 | 0.2976 | 0.5293 | 0.2317 | benefited | benefited | yes | positive | yes |
| mimic_discharge | hpi | keyword | BGE-base | 1 | 512 | 0.7973 | 0.7669 | -0.0303 | harmed | harmed | yes | negative | yes |
| mimic_discharge | hpi | keyword | BioLORD-2023 | 1 | 512 | 0.6002 | 0.5435 | -0.0568 | harmed | harmed | yes | negative | yes |
| mimic_discharge | hpi | keyword | GTE-base | 1 | 512 | 0.825 | 0.7716 | -0.0534 | harmed | harmed | yes | negative | yes |
| mimic_discharge | hpi | keyword | MedCPT | 1 | 512 | 0.6744 | 0.6179 | -0.0565 | harmed | harmed | yes | negative | yes |
| mimic_discharge | hpi | keyword | Nomic-embed-text | 1 | 512 | 0.764 | 0.6787 | -0.0854 | harmed | harmed | yes | negative | yes |
| mimic_discharge | hpi | keyword | Nomic-embed-text-nopfx | 1 | 512 | 0.742 | 0.6913 | -0.0507 | harmed | harmed | yes | negative | yes |
| mimic_discharge | hpi | keyword | BERT-base-uncased | 2 | 512 | 0.1066 | 0.2 | 0.0934 | benefited | benefited | yes | positive | yes |
| mimic_discharge | hpi | keyword | BioBERT | 2 | 512 | 0.1599 | 0.3405 | 0.1807 | benefited | benefited | yes | positive | yes |
| mimic_discharge | hpi | keyword | BioMistral-7B | 2 | 2048 | 0.1938 | 0.3723 | 0.1785 | benefited | benefited | yes | positive | yes |
| mimic_discharge | hpi | keyword | ClinicalBERT | 2 | 512 | 0.1853 | 0.2997 | 0.1145 | benefited | benefited | yes | positive | yes |
| mimic_discharge | hpi | keyword | E5-Mistral-7B | 2 | 4096 | 0.0503 | 0.1819 | 0.1316 | benefited | benefited | yes | positive | yes |
| mimic_discharge | hpi | keyword | E5-Mistral-7B-ablation | 2 | 4096 | 0.3764 | 0.5461 | 0.1697 | benefited | harmed | no | positive | yes |
| mimic_discharge | hpi | keyword | Phi-3-mini | 2 | 4096 | 0.2706 | 0.474 | 0.2034 | benefited | benefited | yes | positive | yes |
| mimic_discharge | hpi | nl | BGE-base | 1 | 512 | 0.8161 | 0.7555 | -0.0607 | harmed | harmed | yes | negative | yes |
| mimic_discharge | hpi | nl | BioLORD-2023 | 1 | 512 | 0.6458 | 0.5587 | -0.0871 | harmed | harmed | yes | negative | yes |
| mimic_discharge | hpi | nl | GTE-base | 1 | 512 | 0.859 | 0.7881 | -0.0709 | harmed | harmed | yes | negative | yes |
| mimic_discharge | hpi | nl | MedCPT | 1 | 512 | 0.4938 | 0.4245 | -0.0693 | harmed | harmed | yes | negative | yes |
| mimic_discharge | hpi | nl | Nomic-embed-text | 1 | 512 | 0.7764 | 0.6945 | -0.0819 | harmed | harmed | yes | negative | yes |
| mimic_discharge | hpi | nl | Nomic-embed-text-nopfx | 1 | 512 | 0.7281 | 0.6436 | -0.0845 | harmed | harmed | yes | negative | yes |
| mimic_discharge | hpi | nl | BERT-base-uncased | 2 | 512 | 0.12 | 0.144 | 0.024 | benefited | benefited | yes | positive | yes |
| mimic_discharge | hpi | nl | BioBERT | 2 | 512 | 0.1257 | 0.2585 | 0.1328 | benefited | benefited | yes | positive | yes |
| mimic_discharge | hpi | nl | BioMistral-7B | 2 | 2048 | 0.2497 | 0.3804 | 0.1307 | benefited | benefited | yes | positive | yes |
| mimic_discharge | hpi | nl | ClinicalBERT | 2 | 512 | 0.1609 | 0.2486 | 0.0877 | benefited | benefited | yes | positive | yes |
| mimic_discharge | hpi | nl | E5-Mistral-7B | 2 | 4096 | 0.0932 | 0.1264 | 0.0332 | benefited | benefited | yes | positive | yes |
| mimic_discharge | hpi | nl | E5-Mistral-7B-ablation | 2 | 4096 | 0.4095 | 0.6837 | 0.2741 | benefited | harmed | no | positive | yes |
| mimic_discharge | hpi | nl | Phi-3-mini | 2 | 4096 | 0.3071 | 0.446 | 0.1389 | benefited | benefited | yes | positive | yes |
