# Supplementary material for: Deriving a Deployment Threshold for Benefit and Harm in Clinical AI: Multi-Institutional Decision-Analytic Modeling Study
Source: JMIR AI. 2026 Oct 23;5:e109863. doi: 10.2196/109863 (PMC13743961; doi:10.2196/109863)
Supplement: Supplementary file 2 [file ai_v5i1e109863_app2.docx]

**Local-screen resampling results**

*Sign-agreement rates by configuration, corpus, query format, and document sample size. The reference sign is the configuration’s full-sample measured effect on that corpus. Intervals are Wilson intervals on the resampling proportion and quantify Monte Carlo precision for these corpora, not spread across sites: the 500 draws are overlapping subsamples of two fixed 100-document corpora, not independent deployment environments. Sample size refers to a joint fit-and-evaluation sample, so the transform-fitting and effect-estimation requirements are not separately identified.*

**Table S1. Resampling agreement by sample size**

| **Corpus** | **Configuration** | **Nominal tier (for reference only)** | **Query format** | **Documents sampled** | **Draws** | **Full-sample dMRR@10** | **Draws with correct sign** | **Agreement rate** | **Wilson lower** | **Wilson upper** | **reference sign** | **contributes to** | **reference basis** |
| --- | --- | --- | --- | --- | --- | --- | --- | --- | --- | --- | --- | --- | --- |
| er_reason | BERT-base-uncased | 2 | keyword | 10 | 500 | 0.1014 | 402 | 0.804 | 0.7669 | 0.8364 | positive | sensitivity | sign of full-sample measured effect on this corpus |
| er_reason | BERT-base-uncased | 2 | keyword | 20 | 500 | 0.1014 | 468 | 0.936 | 0.911 | 0.9543 | positive | sensitivity | sign of full-sample measured effect on this corpus |
| er_reason | BERT-base-uncased | 2 | keyword | 30 | 500 | 0.1014 | 488 | 0.976 | 0.9585 | 0.9862 | positive | sensitivity | sign of full-sample measured effect on this corpus |
| er_reason | BERT-base-uncased | 2 | keyword | 50 | 500 | 0.1014 | 499 | 0.998 | 0.9888 | 0.9996 | positive | sensitivity | sign of full-sample measured effect on this corpus |
| er_reason | BERT-base-uncased | 2 | keyword | 75 | 500 | 0.1014 | 500 | 1.0 | 0.9924 | 1.0 | positive | sensitivity | sign of full-sample measured effect on this corpus |
| er_reason | BERT-base-uncased | 2 | nl | 10 | 500 | 0.0866 | 380 | 0.76 | 0.7207 | 0.7954 | positive | sensitivity | sign of full-sample measured effect on this corpus |
| er_reason | BERT-base-uncased | 2 | nl | 20 | 500 | 0.0866 | 456 | 0.912 | 0.8839 | 0.9338 | positive | sensitivity | sign of full-sample measured effect on this corpus |
| er_reason | BERT-base-uncased | 2 | nl | 30 | 500 | 0.0866 | 475 | 0.95 | 0.9272 | 0.9659 | positive | sensitivity | sign of full-sample measured effect on this corpus |
| er_reason | BERT-base-uncased | 2 | nl | 50 | 500 | 0.0866 | 493 | 0.986 | 0.9714 | 0.9932 | positive | sensitivity | sign of full-sample measured effect on this corpus |
| er_reason | BERT-base-uncased | 2 | nl | 75 | 500 | 0.0866 | 500 | 1.0 | 0.9924 | 1.0 | positive | sensitivity | sign of full-sample measured effect on this corpus |
| er_reason | BioBERT | 2 | keyword | 10 | 500 | 0.1806 | 399 | 0.798 | 0.7606 | 0.8309 | positive | sensitivity | sign of full-sample measured effect on this corpus |
| er_reason | BioBERT | 2 | keyword | 20 | 500 | 0.1806 | 484 | 0.968 | 0.9487 | 0.9802 | positive | sensitivity | sign of full-sample measured effect on this corpus |
| er_reason | BioBERT | 2 | keyword | 30 | 500 | 0.1806 | 498 | 0.996 | 0.9855 | 0.9989 | positive | sensitivity | sign of full-sample measured effect on this corpus |
| er_reason | BioBERT | 2 | keyword | 50 | 500 | 0.1806 | 500 | 1.0 | 0.9924 | 1.0 | positive | sensitivity | sign of full-sample measured effect on this corpus |
| er_reason | BioBERT | 2 | keyword | 75 | 500 | 0.1806 | 500 | 1.0 | 0.9924 | 1.0 | positive | sensitivity | sign of full-sample measured effect on this corpus |
| er_reason | BioBERT | 2 | nl | 10 | 500 | 0.1708 | 400 | 0.8 | 0.7627 | 0.8327 | positive | sensitivity | sign of full-sample measured effect on this corpus |
| er_reason | BioBERT | 2 | nl | 20 | 500 | 0.1708 | 463 | 0.926 | 0.8997 | 0.9458 | positive | sensitivity | sign of full-sample measured effect on this corpus |
| er_reason | BioBERT | 2 | nl | 30 | 500 | 0.1708 | 496 | 0.992 | 0.9796 | 0.9969 | positive | sensitivity | sign of full-sample measured effect on this corpus |
| er_reason | BioBERT | 2 | nl | 50 | 500 | 0.1708 | 500 | 1.0 | 0.9924 | 1.0 | positive | sensitivity | sign of full-sample measured effect on this corpus |
| er_reason | BioBERT | 2 | nl | 75 | 500 | 0.1708 | 500 | 1.0 | 0.9924 | 1.0 | positive | sensitivity | sign of full-sample measured effect on this corpus |
| er_reason | BioMistral-7B | 2 | keyword | 10 | 500 | 0.1113 | 287 | 0.574 | 0.5303 | 0.6166 | positive | sensitivity | sign of full-sample measured effect on this corpus |
| er_reason | BioMistral-7B | 2 | keyword | 20 | 500 | 0.1113 | 382 | 0.764 | 0.7249 | 0.7991 | positive | sensitivity | sign of full-sample measured effect on this corpus |
| er_reason | BioMistral-7B | 2 | keyword | 30 | 500 | 0.1113 | 446 | 0.892 | 0.8617 | 0.9163 | positive | sensitivity | sign of full-sample measured effect on this corpus |
| er_reason | BioMistral-7B | 2 | keyword | 50 | 500 | 0.1113 | 489 | 0.978 | 0.961 | 0.9877 | positive | sensitivity | sign of full-sample measured effect on this corpus |
| er_reason | BioMistral-7B | 2 | keyword | 75 | 500 | 0.1113 | 500 | 1.0 | 0.9924 | 1.0 | positive | sensitivity | sign of full-sample measured effect on this corpus |
| er_reason | BioMistral-7B | 2 | nl | 10 | 500 | 0.2141 | 300 | 0.6 | 0.5565 | 0.642 | positive | sensitivity | sign of full-sample measured effect on this corpus |
| er_reason | BioMistral-7B | 2 | nl | 20 | 500 | 0.2141 | 402 | 0.804 | 0.7669 | 0.8364 | positive | sensitivity | sign of full-sample measured effect on this corpus |
| er_reason | BioMistral-7B | 2 | nl | 30 | 500 | 0.2141 | 474 | 0.948 | 0.9249 | 0.9643 | positive | sensitivity | sign of full-sample measured effect on this corpus |
| er_reason | BioMistral-7B | 2 | nl | 50 | 500 | 0.2141 | 499 | 0.998 | 0.9888 | 0.9996 | positive | sensitivity | sign of full-sample measured effect on this corpus |
| er_reason | BioMistral-7B | 2 | nl | 75 | 500 | 0.2141 | 500 | 1.0 | 0.9924 | 1.0 | positive | sensitivity | sign of full-sample measured effect on this corpus |
| er_reason | ClinicalBERT | 2 | keyword | 10 | 500 | 0.1495 | 347 | 0.694 | 0.6523 | 0.7328 | positive | sensitivity | sign of full-sample measured effect on this corpus |
| er_reason | ClinicalBERT | 2 | keyword | 20 | 500 | 0.1495 | 448 | 0.896 | 0.8662 | 0.9198 | positive | sensitivity | sign of full-sample measured effect on this corpus |
| er_reason | ClinicalBERT | 2 | keyword | 30 | 500 | 0.1495 | 493 | 0.986 | 0.9714 | 0.9932 | positive | sensitivity | sign of full-sample measured effect on this corpus |
| er_reason | ClinicalBERT | 2 | keyword | 50 | 500 | 0.1495 | 500 | 1.0 | 0.9924 | 1.0 | positive | sensitivity | sign of full-sample measured effect on this corpus |
| er_reason | ClinicalBERT | 2 | keyword | 75 | 500 | 0.1495 | 500 | 1.0 | 0.9924 | 1.0 | positive | sensitivity | sign of full-sample measured effect on this corpus |
| er_reason | ClinicalBERT | 2 | nl | 10 | 500 | 0.0869 | 368 | 0.736 | 0.6957 | 0.7727 | positive | sensitivity | sign of full-sample measured effect on this corpus |
| er_reason | ClinicalBERT | 2 | nl | 20 | 500 | 0.0869 | 452 | 0.904 | 0.875 | 0.9268 | positive | sensitivity | sign of full-sample measured effect on this corpus |
| er_reason | ClinicalBERT | 2 | nl | 30 | 500 | 0.0869 | 489 | 0.978 | 0.961 | 0.9877 | positive | sensitivity | sign of full-sample measured effect on this corpus |
| er_reason | ClinicalBERT | 2 | nl | 50 | 500 | 0.0869 | 496 | 0.992 | 0.9796 | 0.9969 | positive | sensitivity | sign of full-sample measured effect on this corpus |
| er_reason | ClinicalBERT | 2 | nl | 75 | 500 | 0.0869 | 500 | 1.0 | 0.9924 | 1.0 | positive | sensitivity | sign of full-sample measured effect on this corpus |
| er_reason | E5-Mistral-7B | 2 | keyword | 10 | 500 | 0.0877 | 338 | 0.676 | 0.6338 | 0.7155 | positive | sensitivity | sign of full-sample measured effect on this corpus |
| er_reason | E5-Mistral-7B | 2 | keyword | 20 | 500 | 0.0877 | 460 | 0.92 | 0.8929 | 0.9407 | positive | sensitivity | sign of full-sample measured effect on this corpus |
| er_reason | E5-Mistral-7B | 2 | keyword | 30 | 500 | 0.0877 | 487 | 0.974 | 0.956 | 0.9847 | positive | sensitivity | sign of full-sample measured effect on this corpus |
| er_reason | E5-Mistral-7B | 2 | keyword | 50 | 500 | 0.0877 | 500 | 1.0 | 0.9924 | 1.0 | positive | sensitivity | sign of full-sample measured effect on this corpus |
| er_reason | E5-Mistral-7B | 2 | keyword | 75 | 500 | 0.0877 | 500 | 1.0 | 0.9924 | 1.0 | positive | sensitivity | sign of full-sample measured effect on this corpus |
| er_reason | E5-Mistral-7B | 2 | nl | 10 | 500 | 0.0504 | 416 | 0.832 | 0.7967 | 0.8622 | positive | sensitivity | sign of full-sample measured effect on this corpus |
| er_reason | E5-Mistral-7B | 2 | nl | 20 | 500 | 0.0504 | 474 | 0.948 | 0.9249 | 0.9643 | positive | sensitivity | sign of full-sample measured effect on this corpus |
| er_reason | E5-Mistral-7B | 2 | nl | 30 | 500 | 0.0504 | 477 | 0.954 | 0.9319 | 0.9692 | positive | sensitivity | sign of full-sample measured effect on this corpus |
| er_reason | E5-Mistral-7B | 2 | nl | 50 | 500 | 0.0504 | 480 | 0.96 | 0.939 | 0.974 | positive | sensitivity | sign of full-sample measured effect on this corpus |
| er_reason | E5-Mistral-7B | 2 | nl | 75 | 500 | 0.0504 | 486 | 0.972 | 0.9536 | 0.9832 | positive | sensitivity | sign of full-sample measured effect on this corpus |
| er_reason | E5-Mistral-7B-ablation | 2 | keyword | 10 | 500 | 0.1746 | 309 | 0.618 | 0.5747 | 0.6595 | positive | sensitivity | sign of full-sample measured effect on this corpus |
| er_reason | E5-Mistral-7B-ablation | 2 | keyword | 20 | 500 | 0.1746 | 361 | 0.722 | 0.6812 | 0.7595 | positive | sensitivity | sign of full-sample measured effect on this corpus |
| er_reason | E5-Mistral-7B-ablation | 2 | keyword | 30 | 500 | 0.1746 | 382 | 0.764 | 0.7249 | 0.7991 | positive | sensitivity | sign of full-sample measured effect on this corpus |
| er_reason | E5-Mistral-7B-ablation | 2 | keyword | 50 | 500 | 0.1746 | 448 | 0.896 | 0.8662 | 0.9198 | positive | sensitivity | sign of full-sample measured effect on this corpus |
| er_reason | E5-Mistral-7B-ablation | 2 | keyword | 75 | 500 | 0.1746 | 499 | 0.998 | 0.9888 | 0.9996 | positive | sensitivity | sign of full-sample measured effect on this corpus |
| er_reason | E5-Mistral-7B-ablation | 2 | nl | 10 | 500 | 0.173 | 299 | 0.598 | 0.5544 | 0.6401 | positive | sensitivity | sign of full-sample measured effect on this corpus |
| er_reason | E5-Mistral-7B-ablation | 2 | nl | 20 | 500 | 0.173 | 381 | 0.762 | 0.7228 | 0.7972 | positive | sensitivity | sign of full-sample measured effect on this corpus |
| er_reason | E5-Mistral-7B-ablation | 2 | nl | 30 | 500 | 0.173 | 410 | 0.82 | 0.7839 | 0.8512 | positive | sensitivity | sign of full-sample measured effect on this corpus |
| er_reason | E5-Mistral-7B-ablation | 2 | nl | 50 | 500 | 0.173 | 443 | 0.886 | 0.8552 | 0.911 | positive | sensitivity | sign of full-sample measured effect on this corpus |
| er_reason | E5-Mistral-7B-ablation | 2 | nl | 75 | 500 | 0.173 | 480 | 0.96 | 0.939 | 0.974 | positive | sensitivity | sign of full-sample measured effect on this corpus |
| er_reason | Phi-3-mini | 2 | keyword | 10 | 500 | 0.2617 | 373 | 0.746 | 0.7061 | 0.7822 | positive | sensitivity | sign of full-sample measured effect on this corpus |
| er_reason | Phi-3-mini | 2 | keyword | 20 | 500 | 0.2617 | 474 | 0.948 | 0.9249 | 0.9643 | positive | sensitivity | sign of full-sample measured effect on this corpus |
| er_reason | Phi-3-mini | 2 | keyword | 30 | 500 | 0.2617 | 491 | 0.982 | 0.9661 | 0.9905 | positive | sensitivity | sign of full-sample measured effect on this corpus |
| er_reason | Phi-3-mini | 2 | keyword | 50 | 500 | 0.2617 | 500 | 1.0 | 0.9924 | 1.0 | positive | sensitivity | sign of full-sample measured effect on this corpus |
| er_reason | Phi-3-mini | 2 | keyword | 75 | 500 | 0.2617 | 500 | 1.0 | 0.9924 | 1.0 | positive | sensitivity | sign of full-sample measured effect on this corpus |
| er_reason | Phi-3-mini | 2 | nl | 10 | 500 | 0.2317 | 351 | 0.702 | 0.6605 | 0.7404 | positive | sensitivity | sign of full-sample measured effect on this corpus |
| er_reason | Phi-3-mini | 2 | nl | 20 | 500 | 0.2317 | 425 | 0.85 | 0.816 | 0.8786 | positive | sensitivity | sign of full-sample measured effect on this corpus |
| er_reason | Phi-3-mini | 2 | nl | 30 | 500 | 0.2317 | 474 | 0.948 | 0.9249 | 0.9643 | positive | sensitivity | sign of full-sample measured effect on this corpus |
| er_reason | Phi-3-mini | 2 | nl | 50 | 500 | 0.2317 | 498 | 0.996 | 0.9855 | 0.9989 | positive | sensitivity | sign of full-sample measured effect on this corpus |
| er_reason | Phi-3-mini | 2 | nl | 75 | 500 | 0.2317 | 500 | 1.0 | 0.9924 | 1.0 | positive | sensitivity | sign of full-sample measured effect on this corpus |
| er_reason | BGE-base | 1 | keyword | 10 | 500 | -0.0954 | 142 | 0.284 | 0.2462 | 0.3251 | negative | specificity | sign of full-sample measured effect on this corpus |
| er_reason | BGE-base | 1 | keyword | 20 | 500 | -0.0954 | 304 | 0.608 | 0.5645 | 0.6498 | negative | specificity | sign of full-sample measured effect on this corpus |
| er_reason | BGE-base | 1 | keyword | 30 | 500 | -0.0954 | 363 | 0.726 | 0.6853 | 0.7633 | negative | specificity | sign of full-sample measured effect on this corpus |
| er_reason | BGE-base | 1 | keyword | 50 | 500 | -0.0954 | 438 | 0.876 | 0.8442 | 0.9021 | negative | specificity | sign of full-sample measured effect on this corpus |
| er_reason | BGE-base | 1 | keyword | 75 | 500 | -0.0954 | 493 | 0.986 | 0.9714 | 0.9932 | negative | specificity | sign of full-sample measured effect on this corpus |
| er_reason | BGE-base | 1 | nl | 10 | 500 | -0.141 | 123 | 0.246 | 0.2103 | 0.2856 | negative | specificity | sign of full-sample measured effect on this corpus |
| er_reason | BGE-base | 1 | nl | 20 | 500 | -0.141 | 274 | 0.548 | 0.5042 | 0.5911 | negative | specificity | sign of full-sample measured effect on this corpus |
| er_reason | BGE-base | 1 | nl | 30 | 500 | -0.141 | 412 | 0.824 | 0.7882 | 0.8549 | negative | specificity | sign of full-sample measured effect on this corpus |
| er_reason | BGE-base | 1 | nl | 50 | 500 | -0.141 | 490 | 0.98 | 0.9636 | 0.9891 | negative | specificity | sign of full-sample measured effect on this corpus |
| er_reason | BGE-base | 1 | nl | 75 | 500 | -0.141 | 500 | 1.0 | 0.9924 | 1.0 | negative | specificity | sign of full-sample measured effect on this corpus |
| er_reason | BioLORD-2023 | 1 | keyword | 10 | 500 | -0.1315 | 311 | 0.622 | 0.5787 | 0.6634 | negative | specificity | sign of full-sample measured effect on this corpus |
| er_reason | BioLORD-2023 | 1 | keyword | 20 | 500 | -0.1315 | 392 | 0.784 | 0.7458 | 0.8178 | negative | specificity | sign of full-sample measured effect on this corpus |
| er_reason | BioLORD-2023 | 1 | keyword | 30 | 500 | -0.1315 | 441 | 0.882 | 0.8508 | 0.9074 | negative | specificity | sign of full-sample measured effect on this corpus |
| er_reason | BioLORD-2023 | 1 | keyword | 50 | 500 | -0.1315 | 481 | 0.962 | 0.9414 | 0.9755 | negative | specificity | sign of full-sample measured effect on this corpus |
| er_reason | BioLORD-2023 | 1 | keyword | 75 | 500 | -0.1315 | 500 | 1.0 | 0.9924 | 1.0 | negative | specificity | sign of full-sample measured effect on this corpus |
| er_reason | BioLORD-2023 | 1 | nl | 10 | 500 | -0.2071 | 247 | 0.494 | 0.4504 | 0.5377 | negative | specificity | sign of full-sample measured effect on this corpus |
| er_reason | BioLORD-2023 | 1 | nl | 20 | 500 | -0.2071 | 431 | 0.862 | 0.829 | 0.8895 | negative | specificity | sign of full-sample measured effect on this corpus |
| er_reason | BioLORD-2023 | 1 | nl | 30 | 500 | -0.2071 | 485 | 0.97 | 0.9511 | 0.9817 | negative | specificity | sign of full-sample measured effect on this corpus |
| er_reason | BioLORD-2023 | 1 | nl | 50 | 500 | -0.2071 | 500 | 1.0 | 0.9924 | 1.0 | negative | specificity | sign of full-sample measured effect on this corpus |
| er_reason | BioLORD-2023 | 1 | nl | 75 | 500 | -0.2071 | 500 | 1.0 | 0.9924 | 1.0 | negative | specificity | sign of full-sample measured effect on this corpus |
| er_reason | GTE-base | 1 | keyword | 10 | 500 | -0.0863 | 173 | 0.346 | 0.3056 | 0.3887 | negative | specificity | sign of full-sample measured effect on this corpus |
| er_reason | GTE-base | 1 | keyword | 20 | 500 | -0.0863 | 322 | 0.644 | 0.6011 | 0.6847 | negative | specificity | sign of full-sample measured effect on this corpus |
| er_reason | GTE-base | 1 | keyword | 30 | 500 | -0.0863 | 364 | 0.728 | 0.6874 | 0.7652 | negative | specificity | sign of full-sample measured effect on this corpus |
| er_reason | GTE-base | 1 | keyword | 50 | 500 | -0.0863 | 425 | 0.85 | 0.816 | 0.8786 | negative | specificity | sign of full-sample measured effect on this corpus |
| er_reason | GTE-base | 1 | keyword | 75 | 500 | -0.0863 | 494 | 0.988 | 0.9741 | 0.9945 | negative | specificity | sign of full-sample measured effect on this corpus |
| er_reason | GTE-base | 1 | nl | 10 | 500 | -0.0925 | 135 | 0.27 | 0.2329 | 0.3106 | negative | specificity | sign of full-sample measured effect on this corpus |
| er_reason | GTE-base | 1 | nl | 20 | 500 | -0.0925 | 301 | 0.602 | 0.5585 | 0.644 | negative | specificity | sign of full-sample measured effect on this corpus |
| er_reason | GTE-base | 1 | nl | 30 | 500 | -0.0925 | 403 | 0.806 | 0.7691 | 0.8383 | negative | specificity | sign of full-sample measured effect on this corpus |
| er_reason | GTE-base | 1 | nl | 50 | 500 | -0.0925 | 484 | 0.968 | 0.9487 | 0.9802 | negative | specificity | sign of full-sample measured effect on this corpus |
| er_reason | GTE-base | 1 | nl | 75 | 500 | -0.0925 | 500 | 1.0 | 0.9924 | 1.0 | negative | specificity | sign of full-sample measured effect on this corpus |
| er_reason | MedCPT | 1 | keyword | 10 | 500 | -0.2162 | 181 | 0.362 | 0.3211 | 0.405 | negative | specificity | sign of full-sample measured effect on this corpus |
| er_reason | MedCPT | 1 | keyword | 20 | 500 | -0.2162 | 342 | 0.684 | 0.642 | 0.7232 | negative | specificity | sign of full-sample measured effect on this corpus |
| er_reason | MedCPT | 1 | keyword | 30 | 500 | -0.2162 | 422 | 0.844 | 0.8096 | 0.8732 | negative | specificity | sign of full-sample measured effect on this corpus |
| er_reason | MedCPT | 1 | keyword | 50 | 500 | -0.2162 | 499 | 0.998 | 0.9888 | 0.9996 | negative | specificity | sign of full-sample measured effect on this corpus |
| er_reason | MedCPT | 1 | keyword | 75 | 500 | -0.2162 | 500 | 1.0 | 0.9924 | 1.0 | negative | specificity | sign of full-sample measured effect on this corpus |
| er_reason | MedCPT | 1 | nl | 10 | 500 | -0.1552 | 151 | 0.302 | 0.2634 | 0.3436 | negative | specificity | sign of full-sample measured effect on this corpus |
| er_reason | MedCPT | 1 | nl | 20 | 500 | -0.1552 | 208 | 0.416 | 0.3736 | 0.4597 | negative | specificity | sign of full-sample measured effect on this corpus |
| er_reason | MedCPT | 1 | nl | 30 | 500 | -0.1552 | 236 | 0.472 | 0.4286 | 0.5158 | negative | specificity | sign of full-sample measured effect on this corpus |
| er_reason | MedCPT | 1 | nl | 50 | 500 | -0.1552 | 312 | 0.624 | 0.5807 | 0.6654 | negative | specificity | sign of full-sample measured effect on this corpus |
| er_reason | MedCPT | 1 | nl | 75 | 500 | -0.1552 | 476 | 0.952 | 0.9296 | 0.9675 | negative | specificity | sign of full-sample measured effect on this corpus |
| er_reason | Nomic-embed-text | 1 | keyword | 10 | 500 | -0.0958 | 167 | 0.334 | 0.2941 | 0.3765 | negative | specificity | sign of full-sample measured effect on this corpus |
| er_reason | Nomic-embed-text | 1 | keyword | 20 | 500 | -0.0958 | 316 | 0.632 | 0.5889 | 0.6731 | negative | specificity | sign of full-sample measured effect on this corpus |
| er_reason | Nomic-embed-text | 1 | keyword | 30 | 500 | -0.0958 | 404 | 0.808 | 0.7712 | 0.8401 | negative | specificity | sign of full-sample measured effect on this corpus |
| er_reason | Nomic-embed-text | 1 | keyword | 50 | 500 | -0.0958 | 472 | 0.944 | 0.9203 | 0.961 | negative | specificity | sign of full-sample measured effect on this corpus |
| er_reason | Nomic-embed-text | 1 | keyword | 75 | 500 | -0.0958 | 500 | 1.0 | 0.9924 | 1.0 | negative | specificity | sign of full-sample measured effect on this corpus |
| er_reason | Nomic-embed-text | 1 | nl | 10 | 500 | -0.0785 | 121 | 0.242 | 0.2065 | 0.2814 | negative | specificity | sign of full-sample measured effect on this corpus |
| er_reason | Nomic-embed-text | 1 | nl | 20 | 500 | -0.0785 | 267 | 0.534 | 0.4902 | 0.5773 | negative | specificity | sign of full-sample measured effect on this corpus |
| er_reason | Nomic-embed-text | 1 | nl | 30 | 500 | -0.0785 | 355 | 0.71 | 0.6687 | 0.7481 | negative | specificity | sign of full-sample measured effect on this corpus |
| er_reason | Nomic-embed-text | 1 | nl | 50 | 500 | -0.0785 | 462 | 0.924 | 0.8974 | 0.9441 | negative | specificity | sign of full-sample measured effect on this corpus |
| er_reason | Nomic-embed-text | 1 | nl | 75 | 500 | -0.0785 | 500 | 1.0 | 0.9924 | 1.0 | negative | specificity | sign of full-sample measured effect on this corpus |
| er_reason | Nomic-embed-text-nopfx | 1 | keyword | 10 | 500 | -0.1089 | 166 | 0.332 | 0.2921 | 0.3744 | negative | specificity | sign of full-sample measured effect on this corpus |
| er_reason | Nomic-embed-text-nopfx | 1 | keyword | 20 | 500 | -0.1089 | 293 | 0.586 | 0.5423 | 0.6284 | negative | specificity | sign of full-sample measured effect on this corpus |
| er_reason | Nomic-embed-text-nopfx | 1 | keyword | 30 | 500 | -0.1089 | 388 | 0.776 | 0.7374 | 0.8104 | negative | specificity | sign of full-sample measured effect on this corpus |
| er_reason | Nomic-embed-text-nopfx | 1 | keyword | 50 | 500 | -0.1089 | 489 | 0.978 | 0.961 | 0.9877 | negative | specificity | sign of full-sample measured effect on this corpus |
| er_reason | Nomic-embed-text-nopfx | 1 | keyword | 75 | 500 | -0.1089 | 500 | 1.0 | 0.9924 | 1.0 | negative | specificity | sign of full-sample measured effect on this corpus |
| er_reason | Nomic-embed-text-nopfx | 1 | nl | 10 | 500 | -0.227 | 110 | 0.22 | 0.1859 | 0.2584 | negative | specificity | sign of full-sample measured effect on this corpus |
| er_reason | Nomic-embed-text-nopfx | 1 | nl | 20 | 500 | -0.227 | 179 | 0.358 | 0.3172 | 0.401 | negative | specificity | sign of full-sample measured effect on this corpus |
| er_reason | Nomic-embed-text-nopfx | 1 | nl | 30 | 500 | -0.227 | 313 | 0.626 | 0.5828 | 0.6673 | negative | specificity | sign of full-sample measured effect on this corpus |
| er_reason | Nomic-embed-text-nopfx | 1 | nl | 50 | 500 | -0.227 | 439 | 0.878 | 0.8464 | 0.9038 | negative | specificity | sign of full-sample measured effect on this corpus |
| er_reason | Nomic-embed-text-nopfx | 1 | nl | 75 | 500 | -0.227 | 499 | 0.998 | 0.9888 | 0.9996 | negative | specificity | sign of full-sample measured effect on this corpus |
| mimic_discharge | BERT-base-uncased | 2 | keyword | 10 | 500 | 0.0934 | 313 | 0.626 | 0.5828 | 0.6673 | positive | sensitivity | sign of full-sample measured effect on this corpus |
| mimic_discharge | BERT-base-uncased | 2 | keyword | 20 | 500 | 0.0934 | 408 | 0.816 | 0.7797 | 0.8475 | positive | sensitivity | sign of full-sample measured effect on this corpus |
| mimic_discharge | BERT-base-uncased | 2 | keyword | 30 | 500 | 0.0934 | 466 | 0.932 | 0.9065 | 0.9509 | positive | sensitivity | sign of full-sample measured effect on this corpus |
| mimic_discharge | BERT-base-uncased | 2 | keyword | 50 | 500 | 0.0934 | 492 | 0.984 | 0.9687 | 0.9919 | positive | sensitivity | sign of full-sample measured effect on this corpus |
| mimic_discharge | BERT-base-uncased | 2 | keyword | 75 | 500 | 0.0934 | 500 | 1.0 | 0.9924 | 1.0 | positive | sensitivity | sign of full-sample measured effect on this corpus |
| mimic_discharge | BERT-base-uncased | 2 | nl | 10 | 500 | 0.024 | 320 | 0.64 | 0.597 | 0.6809 | positive | sensitivity | sign of full-sample measured effect on this corpus |
| mimic_discharge | BERT-base-uncased | 2 | nl | 20 | 500 | 0.024 | 363 | 0.726 | 0.6853 | 0.7633 | positive | sensitivity | sign of full-sample measured effect on this corpus |
| mimic_discharge | BERT-base-uncased | 2 | nl | 30 | 500 | 0.024 | 408 | 0.816 | 0.7797 | 0.8475 | positive | sensitivity | sign of full-sample measured effect on this corpus |
| mimic_discharge | BERT-base-uncased | 2 | nl | 50 | 500 | 0.024 | 460 | 0.92 | 0.8929 | 0.9407 | positive | sensitivity | sign of full-sample measured effect on this corpus |
| mimic_discharge | BERT-base-uncased | 2 | nl | 75 | 500 | 0.024 | 479 | 0.958 | 0.9366 | 0.9724 | positive | sensitivity | sign of full-sample measured effect on this corpus |
| mimic_discharge | BioBERT | 2 | keyword | 10 | 500 | 0.1807 | 411 | 0.822 | 0.7861 | 0.853 | positive | sensitivity | sign of full-sample measured effect on this corpus |
| mimic_discharge | BioBERT | 2 | keyword | 20 | 500 | 0.1807 | 496 | 0.992 | 0.9796 | 0.9969 | positive | sensitivity | sign of full-sample measured effect on this corpus |
| mimic_discharge | BioBERT | 2 | keyword | 30 | 500 | 0.1807 | 500 | 1.0 | 0.9924 | 1.0 | positive | sensitivity | sign of full-sample measured effect on this corpus |
| mimic_discharge | BioBERT | 2 | keyword | 50 | 500 | 0.1807 | 500 | 1.0 | 0.9924 | 1.0 | positive | sensitivity | sign of full-sample measured effect on this corpus |
| mimic_discharge | BioBERT | 2 | keyword | 75 | 500 | 0.1807 | 500 | 1.0 | 0.9924 | 1.0 | positive | sensitivity | sign of full-sample measured effect on this corpus |
| mimic_discharge | BioBERT | 2 | nl | 10 | 500 | 0.1328 | 358 | 0.716 | 0.6749 | 0.7538 | positive | sensitivity | sign of full-sample measured effect on this corpus |
| mimic_discharge | BioBERT | 2 | nl | 20 | 500 | 0.1328 | 458 | 0.916 | 0.8884 | 0.9373 | positive | sensitivity | sign of full-sample measured effect on this corpus |
| mimic_discharge | BioBERT | 2 | nl | 30 | 500 | 0.1328 | 491 | 0.982 | 0.9661 | 0.9905 | positive | sensitivity | sign of full-sample measured effect on this corpus |
| mimic_discharge | BioBERT | 2 | nl | 50 | 500 | 0.1328 | 498 | 0.996 | 0.9855 | 0.9989 | positive | sensitivity | sign of full-sample measured effect on this corpus |
| mimic_discharge | BioBERT | 2 | nl | 75 | 500 | 0.1328 | 500 | 1.0 | 0.9924 | 1.0 | positive | sensitivity | sign of full-sample measured effect on this corpus |
| mimic_discharge | BioMistral-7B | 2 | keyword | 10 | 500 | 0.1785 | 266 | 0.532 | 0.4882 | 0.5753 | positive | sensitivity | sign of full-sample measured effect on this corpus |
| mimic_discharge | BioMistral-7B | 2 | keyword | 20 | 500 | 0.1785 | 379 | 0.758 | 0.7186 | 0.7935 | positive | sensitivity | sign of full-sample measured effect on this corpus |
| mimic_discharge | BioMistral-7B | 2 | keyword | 30 | 500 | 0.1785 | 451 | 0.902 | 0.8728 | 0.9251 | positive | sensitivity | sign of full-sample measured effect on this corpus |
| mimic_discharge | BioMistral-7B | 2 | keyword | 50 | 500 | 0.1785 | 497 | 0.994 | 0.9825 | 0.998 | positive | sensitivity | sign of full-sample measured effect on this corpus |
| mimic_discharge | BioMistral-7B | 2 | keyword | 75 | 500 | 0.1785 | 500 | 1.0 | 0.9924 | 1.0 | positive | sensitivity | sign of full-sample measured effect on this corpus |
| mimic_discharge | BioMistral-7B | 2 | nl | 10 | 500 | 0.1307 | 318 | 0.636 | 0.5929 | 0.677 | positive | sensitivity | sign of full-sample measured effect on this corpus |
| mimic_discharge | BioMistral-7B | 2 | nl | 20 | 500 | 0.1307 | 331 | 0.662 | 0.6194 | 0.7021 | positive | sensitivity | sign of full-sample measured effect on this corpus |
| mimic_discharge | BioMistral-7B | 2 | nl | 30 | 500 | 0.1307 | 401 | 0.802 | 0.7648 | 0.8346 | positive | sensitivity | sign of full-sample measured effect on this corpus |
| mimic_discharge | BioMistral-7B | 2 | nl | 50 | 500 | 0.1307 | 464 | 0.928 | 0.9019 | 0.9475 | positive | sensitivity | sign of full-sample measured effect on this corpus |
| mimic_discharge | BioMistral-7B | 2 | nl | 75 | 500 | 0.1307 | 500 | 1.0 | 0.9924 | 1.0 | positive | sensitivity | sign of full-sample measured effect on this corpus |
| mimic_discharge | ClinicalBERT | 2 | keyword | 10 | 500 | 0.1145 | 379 | 0.758 | 0.7186 | 0.7935 | positive | sensitivity | sign of full-sample measured effect on this corpus |
| mimic_discharge | ClinicalBERT | 2 | keyword | 20 | 500 | 0.1145 | 463 | 0.926 | 0.8997 | 0.9458 | positive | sensitivity | sign of full-sample measured effect on this corpus |
| mimic_discharge | ClinicalBERT | 2 | keyword | 30 | 500 | 0.1145 | 490 | 0.98 | 0.9636 | 0.9891 | positive | sensitivity | sign of full-sample measured effect on this corpus |
| mimic_discharge | ClinicalBERT | 2 | keyword | 50 | 500 | 0.1145 | 499 | 0.998 | 0.9888 | 0.9996 | positive | sensitivity | sign of full-sample measured effect on this corpus |
| mimic_discharge | ClinicalBERT | 2 | keyword | 75 | 500 | 0.1145 | 500 | 1.0 | 0.9924 | 1.0 | positive | sensitivity | sign of full-sample measured effect on this corpus |
| mimic_discharge | ClinicalBERT | 2 | nl | 10 | 500 | 0.0877 | 296 | 0.592 | 0.5484 | 0.6342 | positive | sensitivity | sign of full-sample measured effect on this corpus |
| mimic_discharge | ClinicalBERT | 2 | nl | 20 | 500 | 0.0877 | 405 | 0.81 | 0.7733 | 0.842 | positive | sensitivity | sign of full-sample measured effect on this corpus |
| mimic_discharge | ClinicalBERT | 2 | nl | 30 | 500 | 0.0877 | 435 | 0.87 | 0.8377 | 0.8967 | positive | sensitivity | sign of full-sample measured effect on this corpus |
| mimic_discharge | ClinicalBERT | 2 | nl | 50 | 500 | 0.0877 | 489 | 0.978 | 0.961 | 0.9877 | positive | sensitivity | sign of full-sample measured effect on this corpus |
| mimic_discharge | ClinicalBERT | 2 | nl | 75 | 500 | 0.0877 | 499 | 0.998 | 0.9888 | 0.9996 | positive | sensitivity | sign of full-sample measured effect on this corpus |
| mimic_discharge | E5-Mistral-7B | 2 | keyword | 10 | 500 | 0.1316 | 369 | 0.738 | 0.6977 | 0.7746 | positive | sensitivity | sign of full-sample measured effect on this corpus |
| mimic_discharge | E5-Mistral-7B | 2 | keyword | 20 | 500 | 0.1316 | 478 | 0.956 | 0.9343 | 0.9708 | positive | sensitivity | sign of full-sample measured effect on this corpus |
| mimic_discharge | E5-Mistral-7B | 2 | keyword | 30 | 500 | 0.1316 | 499 | 0.998 | 0.9888 | 0.9996 | positive | sensitivity | sign of full-sample measured effect on this corpus |
| mimic_discharge | E5-Mistral-7B | 2 | keyword | 50 | 500 | 0.1316 | 500 | 1.0 | 0.9924 | 1.0 | positive | sensitivity | sign of full-sample measured effect on this corpus |
| mimic_discharge | E5-Mistral-7B | 2 | keyword | 75 | 500 | 0.1316 | 500 | 1.0 | 0.9924 | 1.0 | positive | sensitivity | sign of full-sample measured effect on this corpus |
| mimic_discharge | E5-Mistral-7B | 2 | nl | 10 | 500 | 0.0332 | 355 | 0.71 | 0.6687 | 0.7481 | positive | sensitivity | sign of full-sample measured effect on this corpus |
| mimic_discharge | E5-Mistral-7B | 2 | nl | 20 | 500 | 0.0332 | 418 | 0.836 | 0.801 | 0.8659 | positive | sensitivity | sign of full-sample measured effect on this corpus |
| mimic_discharge | E5-Mistral-7B | 2 | nl | 30 | 500 | 0.0332 | 458 | 0.916 | 0.8884 | 0.9373 | positive | sensitivity | sign of full-sample measured effect on this corpus |
| mimic_discharge | E5-Mistral-7B | 2 | nl | 50 | 500 | 0.0332 | 478 | 0.956 | 0.9343 | 0.9708 | positive | sensitivity | sign of full-sample measured effect on this corpus |
| mimic_discharge | E5-Mistral-7B | 2 | nl | 75 | 500 | 0.0332 | 494 | 0.988 | 0.9741 | 0.9945 | positive | sensitivity | sign of full-sample measured effect on this corpus |
| mimic_discharge | E5-Mistral-7B-ablation | 2 | keyword | 10 | 500 | 0.1697 | 266 | 0.532 | 0.4882 | 0.5753 | positive | sensitivity | sign of full-sample measured effect on this corpus |
| mimic_discharge | E5-Mistral-7B-ablation | 2 | keyword | 20 | 500 | 0.1697 | 393 | 0.786 | 0.7479 | 0.8197 | positive | sensitivity | sign of full-sample measured effect on this corpus |
| mimic_discharge | E5-Mistral-7B-ablation | 2 | keyword | 30 | 500 | 0.1697 | 435 | 0.87 | 0.8377 | 0.8967 | positive | sensitivity | sign of full-sample measured effect on this corpus |
| mimic_discharge | E5-Mistral-7B-ablation | 2 | keyword | 50 | 500 | 0.1697 | 489 | 0.978 | 0.961 | 0.9877 | positive | sensitivity | sign of full-sample measured effect on this corpus |
| mimic_discharge | E5-Mistral-7B-ablation | 2 | keyword | 75 | 500 | 0.1697 | 500 | 1.0 | 0.9924 | 1.0 | positive | sensitivity | sign of full-sample measured effect on this corpus |
| mimic_discharge | E5-Mistral-7B-ablation | 2 | nl | 10 | 500 | 0.2741 | 366 | 0.732 | 0.6915 | 0.7689 | positive | sensitivity | sign of full-sample measured effect on this corpus |
| mimic_discharge | E5-Mistral-7B-ablation | 2 | nl | 20 | 500 | 0.2741 | 453 | 0.906 | 0.8772 | 0.9286 | positive | sensitivity | sign of full-sample measured effect on this corpus |
| mimic_discharge | E5-Mistral-7B-ablation | 2 | nl | 30 | 500 | 0.2741 | 480 | 0.96 | 0.939 | 0.974 | positive | sensitivity | sign of full-sample measured effect on this corpus |
| mimic_discharge | E5-Mistral-7B-ablation | 2 | nl | 50 | 500 | 0.2741 | 500 | 1.0 | 0.9924 | 1.0 | positive | sensitivity | sign of full-sample measured effect on this corpus |
| mimic_discharge | E5-Mistral-7B-ablation | 2 | nl | 75 | 500 | 0.2741 | 500 | 1.0 | 0.9924 | 1.0 | positive | sensitivity | sign of full-sample measured effect on this corpus |
| mimic_discharge | Phi-3-mini | 2 | keyword | 10 | 500 | 0.2034 | 340 | 0.68 | 0.6379 | 0.7194 | positive | sensitivity | sign of full-sample measured effect on this corpus |
| mimic_discharge | Phi-3-mini | 2 | keyword | 20 | 500 | 0.2034 | 464 | 0.928 | 0.9019 | 0.9475 | positive | sensitivity | sign of full-sample measured effect on this corpus |
| mimic_discharge | Phi-3-mini | 2 | keyword | 30 | 500 | 0.2034 | 479 | 0.958 | 0.9366 | 0.9724 | positive | sensitivity | sign of full-sample measured effect on this corpus |
| mimic_discharge | Phi-3-mini | 2 | keyword | 50 | 500 | 0.2034 | 500 | 1.0 | 0.9924 | 1.0 | positive | sensitivity | sign of full-sample measured effect on this corpus |
| mimic_discharge | Phi-3-mini | 2 | keyword | 75 | 500 | 0.2034 | 500 | 1.0 | 0.9924 | 1.0 | positive | sensitivity | sign of full-sample measured effect on this corpus |
| mimic_discharge | Phi-3-mini | 2 | nl | 10 | 500 | 0.1389 | 343 | 0.686 | 0.644 | 0.7251 | positive | sensitivity | sign of full-sample measured effect on this corpus |
| mimic_discharge | Phi-3-mini | 2 | nl | 20 | 500 | 0.1389 | 430 | 0.86 | 0.8268 | 0.8877 | positive | sensitivity | sign of full-sample measured effect on this corpus |
| mimic_discharge | Phi-3-mini | 2 | nl | 30 | 500 | 0.1389 | 450 | 0.9 | 0.8706 | 0.9233 | positive | sensitivity | sign of full-sample measured effect on this corpus |
| mimic_discharge | Phi-3-mini | 2 | nl | 50 | 500 | 0.1389 | 491 | 0.982 | 0.9661 | 0.9905 | positive | sensitivity | sign of full-sample measured effect on this corpus |
| mimic_discharge | Phi-3-mini | 2 | nl | 75 | 500 | 0.1389 | 500 | 1.0 | 0.9924 | 1.0 | positive | sensitivity | sign of full-sample measured effect on this corpus |
| mimic_discharge | BGE-base | 1 | keyword | 10 | 500 | -0.0303 | 136 | 0.272 | 0.2348 | 0.3126 | negative | specificity | sign of full-sample measured effect on this corpus |
| mimic_discharge | BGE-base | 1 | keyword | 20 | 500 | -0.0303 | 271 | 0.542 | 0.4982 | 0.5852 | negative | specificity | sign of full-sample measured effect on this corpus |
| mimic_discharge | BGE-base | 1 | keyword | 30 | 500 | -0.0303 | 330 | 0.66 | 0.6174 | 0.7002 | negative | specificity | sign of full-sample measured effect on this corpus |
| mimic_discharge | BGE-base | 1 | keyword | 50 | 500 | -0.0303 | 405 | 0.81 | 0.7733 | 0.842 | negative | specificity | sign of full-sample measured effect on this corpus |
| mimic_discharge | BGE-base | 1 | keyword | 75 | 500 | -0.0303 | 473 | 0.946 | 0.9226 | 0.9626 | negative | specificity | sign of full-sample measured effect on this corpus |
| mimic_discharge | BGE-base | 1 | nl | 10 | 500 | -0.0607 | 103 | 0.206 | 0.1729 | 0.2436 | negative | specificity | sign of full-sample measured effect on this corpus |
| mimic_discharge | BGE-base | 1 | nl | 20 | 500 | -0.0607 | 194 | 0.388 | 0.3463 | 0.4314 | negative | specificity | sign of full-sample measured effect on this corpus |
| mimic_discharge | BGE-base | 1 | nl | 30 | 500 | -0.0607 | 252 | 0.504 | 0.4603 | 0.5476 | negative | specificity | sign of full-sample measured effect on this corpus |
| mimic_discharge | BGE-base | 1 | nl | 50 | 500 | -0.0607 | 372 | 0.744 | 0.704 | 0.7803 | negative | specificity | sign of full-sample measured effect on this corpus |
| mimic_discharge | BGE-base | 1 | nl | 75 | 500 | -0.0607 | 489 | 0.978 | 0.961 | 0.9877 | negative | specificity | sign of full-sample measured effect on this corpus |
| mimic_discharge | BioLORD-2023 | 1 | keyword | 10 | 500 | -0.0568 | 315 | 0.63 | 0.5868 | 0.6712 | negative | specificity | sign of full-sample measured effect on this corpus |
| mimic_discharge | BioLORD-2023 | 1 | keyword | 20 | 500 | -0.0568 | 411 | 0.822 | 0.7861 | 0.853 | negative | specificity | sign of full-sample measured effect on this corpus |
| mimic_discharge | BioLORD-2023 | 1 | keyword | 30 | 500 | -0.0568 | 429 | 0.858 | 0.8247 | 0.8859 | negative | specificity | sign of full-sample measured effect on this corpus |
| mimic_discharge | BioLORD-2023 | 1 | keyword | 50 | 500 | -0.0568 | 479 | 0.958 | 0.9366 | 0.9724 | negative | specificity | sign of full-sample measured effect on this corpus |
| mimic_discharge | BioLORD-2023 | 1 | keyword | 75 | 500 | -0.0568 | 500 | 1.0 | 0.9924 | 1.0 | negative | specificity | sign of full-sample measured effect on this corpus |
| mimic_discharge | BioLORD-2023 | 1 | nl | 10 | 500 | -0.0871 | 239 | 0.478 | 0.4346 | 0.5218 | negative | specificity | sign of full-sample measured effect on this corpus |
| mimic_discharge | BioLORD-2023 | 1 | nl | 20 | 500 | -0.0871 | 349 | 0.698 | 0.6564 | 0.7366 | negative | specificity | sign of full-sample measured effect on this corpus |
| mimic_discharge | BioLORD-2023 | 1 | nl | 30 | 500 | -0.0871 | 390 | 0.78 | 0.7416 | 0.8141 | negative | specificity | sign of full-sample measured effect on this corpus |
| mimic_discharge | BioLORD-2023 | 1 | nl | 50 | 500 | -0.0871 | 455 | 0.91 | 0.8817 | 0.9321 | negative | specificity | sign of full-sample measured effect on this corpus |
| mimic_discharge | BioLORD-2023 | 1 | nl | 75 | 500 | -0.0871 | 498 | 0.996 | 0.9855 | 0.9989 | negative | specificity | sign of full-sample measured effect on this corpus |
| mimic_discharge | GTE-base | 1 | keyword | 10 | 500 | -0.0534 | 149 | 0.298 | 0.2596 | 0.3395 | negative | specificity | sign of full-sample measured effect on this corpus |
| mimic_discharge | GTE-base | 1 | keyword | 20 | 500 | -0.0534 | 338 | 0.676 | 0.6338 | 0.7155 | negative | specificity | sign of full-sample measured effect on this corpus |
| mimic_discharge | GTE-base | 1 | keyword | 30 | 500 | -0.0534 | 407 | 0.814 | 0.7775 | 0.8457 | negative | specificity | sign of full-sample measured effect on this corpus |
| mimic_discharge | GTE-base | 1 | keyword | 50 | 500 | -0.0534 | 461 | 0.922 | 0.8951 | 0.9424 | negative | specificity | sign of full-sample measured effect on this corpus |
| mimic_discharge | GTE-base | 1 | keyword | 75 | 500 | -0.0534 | 491 | 0.982 | 0.9661 | 0.9905 | negative | specificity | sign of full-sample measured effect on this corpus |
| mimic_discharge | GTE-base | 1 | nl | 10 | 500 | -0.0709 | 93 | 0.186 | 0.1543 | 0.2225 | negative | specificity | sign of full-sample measured effect on this corpus |
| mimic_discharge | GTE-base | 1 | nl | 20 | 500 | -0.0709 | 254 | 0.508 | 0.4643 | 0.5516 | negative | specificity | sign of full-sample measured effect on this corpus |
| mimic_discharge | GTE-base | 1 | nl | 30 | 500 | -0.0709 | 309 | 0.618 | 0.5747 | 0.6595 | negative | specificity | sign of full-sample measured effect on this corpus |
| mimic_discharge | GTE-base | 1 | nl | 50 | 500 | -0.0709 | 444 | 0.888 | 0.8573 | 0.9127 | negative | specificity | sign of full-sample measured effect on this corpus |
| mimic_discharge | GTE-base | 1 | nl | 75 | 500 | -0.0709 | 495 | 0.99 | 0.9768 | 0.9957 | negative | specificity | sign of full-sample measured effect on this corpus |
| mimic_discharge | MedCPT | 1 | keyword | 10 | 500 | -0.0565 | 118 | 0.236 | 0.2009 | 0.2751 | negative | specificity | sign of full-sample measured effect on this corpus |
| mimic_discharge | MedCPT | 1 | keyword | 20 | 500 | -0.0565 | 199 | 0.398 | 0.356 | 0.4415 | negative | specificity | sign of full-sample measured effect on this corpus |
| mimic_discharge | MedCPT | 1 | keyword | 30 | 500 | -0.0565 | 221 | 0.442 | 0.3991 | 0.4858 | negative | specificity | sign of full-sample measured effect on this corpus |
| mimic_discharge | MedCPT | 1 | keyword | 50 | 500 | -0.0565 | 302 | 0.604 | 0.5605 | 0.6459 | negative | specificity | sign of full-sample measured effect on this corpus |
| mimic_discharge | MedCPT | 1 | keyword | 75 | 500 | -0.0565 | 448 | 0.896 | 0.8662 | 0.9198 | negative | specificity | sign of full-sample measured effect on this corpus |
| mimic_discharge | MedCPT | 1 | nl | 10 | 500 | -0.0693 | 150 | 0.3 | 0.2615 | 0.3416 | negative | specificity | sign of full-sample measured effect on this corpus |
| mimic_discharge | MedCPT | 1 | nl | 20 | 500 | -0.0693 | 123 | 0.246 | 0.2103 | 0.2856 | negative | specificity | sign of full-sample measured effect on this corpus |
| mimic_discharge | MedCPT | 1 | nl | 30 | 500 | -0.0693 | 123 | 0.246 | 0.2103 | 0.2856 | negative | specificity | sign of full-sample measured effect on this corpus |
| mimic_discharge | MedCPT | 1 | nl | 50 | 500 | -0.0693 | 188 | 0.376 | 0.3346 | 0.4193 | negative | specificity | sign of full-sample measured effect on this corpus |
| mimic_discharge | MedCPT | 1 | nl | 75 | 500 | -0.0693 | 348 | 0.696 | 0.6543 | 0.7347 | negative | specificity | sign of full-sample measured effect on this corpus |
| mimic_discharge | Nomic-embed-text | 1 | keyword | 10 | 500 | -0.0854 | 115 | 0.23 | 0.1953 | 0.2689 | negative | specificity | sign of full-sample measured effect on this corpus |
| mimic_discharge | Nomic-embed-text | 1 | keyword | 20 | 500 | -0.0854 | 236 | 0.472 | 0.4286 | 0.5158 | negative | specificity | sign of full-sample measured effect on this corpus |
| mimic_discharge | Nomic-embed-text | 1 | keyword | 30 | 500 | -0.0854 | 296 | 0.592 | 0.5484 | 0.6342 | negative | specificity | sign of full-sample measured effect on this corpus |
| mimic_discharge | Nomic-embed-text | 1 | keyword | 50 | 500 | -0.0854 | 413 | 0.826 | 0.7903 | 0.8567 | negative | specificity | sign of full-sample measured effect on this corpus |
| mimic_discharge | Nomic-embed-text | 1 | keyword | 75 | 500 | -0.0854 | 498 | 0.996 | 0.9855 | 0.9989 | negative | specificity | sign of full-sample measured effect on this corpus |
| mimic_discharge | Nomic-embed-text | 1 | nl | 10 | 500 | -0.0819 | 130 | 0.26 | 0.2235 | 0.3002 | negative | specificity | sign of full-sample measured effect on this corpus |
| mimic_discharge | Nomic-embed-text | 1 | nl | 20 | 500 | -0.0819 | 231 | 0.462 | 0.4188 | 0.5058 | negative | specificity | sign of full-sample measured effect on this corpus |
| mimic_discharge | Nomic-embed-text | 1 | nl | 30 | 500 | -0.0819 | 282 | 0.564 | 0.5202 | 0.6068 | negative | specificity | sign of full-sample measured effect on this corpus |
| mimic_discharge | Nomic-embed-text | 1 | nl | 50 | 500 | -0.0819 | 449 | 0.898 | 0.8684 | 0.9216 | negative | specificity | sign of full-sample measured effect on this corpus |
| mimic_discharge | Nomic-embed-text | 1 | nl | 75 | 500 | -0.0819 | 500 | 1.0 | 0.9924 | 1.0 | negative | specificity | sign of full-sample measured effect on this corpus |
| mimic_discharge | Nomic-embed-text-nopfx | 1 | keyword | 10 | 500 | -0.0507 | 240 | 0.48 | 0.4365 | 0.5238 | negative | specificity | sign of full-sample measured effect on this corpus |
| mimic_discharge | Nomic-embed-text-nopfx | 1 | keyword | 20 | 500 | -0.0507 | 331 | 0.662 | 0.6194 | 0.7021 | negative | specificity | sign of full-sample measured effect on this corpus |
| mimic_discharge | Nomic-embed-text-nopfx | 1 | keyword | 30 | 500 | -0.0507 | 376 | 0.752 | 0.7123 | 0.7878 | negative | specificity | sign of full-sample measured effect on this corpus |
| mimic_discharge | Nomic-embed-text-nopfx | 1 | keyword | 50 | 500 | -0.0507 | 427 | 0.854 | 0.8204 | 0.8823 | negative | specificity | sign of full-sample measured effect on this corpus |
| mimic_discharge | Nomic-embed-text-nopfx | 1 | keyword | 75 | 500 | -0.0507 | 486 | 0.972 | 0.9536 | 0.9832 | negative | specificity | sign of full-sample measured effect on this corpus |
| mimic_discharge | Nomic-embed-text-nopfx | 1 | nl | 10 | 500 | -0.0845 | 125 | 0.25 | 0.214 | 0.2898 | negative | specificity | sign of full-sample measured effect on this corpus |
| mimic_discharge | Nomic-embed-text-nopfx | 1 | nl | 20 | 500 | -0.0845 | 245 | 0.49 | 0.4464 | 0.5337 | negative | specificity | sign of full-sample measured effect on this corpus |
| mimic_discharge | Nomic-embed-text-nopfx | 1 | nl | 30 | 500 | -0.0845 | 350 | 0.7 | 0.6584 | 0.7385 | negative | specificity | sign of full-sample measured effect on this corpus |
| mimic_discharge | Nomic-embed-text-nopfx | 1 | nl | 50 | 500 | -0.0845 | 453 | 0.906 | 0.8772 | 0.9286 | negative | specificity | sign of full-sample measured effect on this corpus |
| mimic_discharge | Nomic-embed-text-nopfx | 1 | nl | 75 | 500 | -0.0845 | 496 | 0.992 | 0.9796 | 0.9969 | negative | specificity | sign of full-sample measured effect on this corpus |
