# Supplementary material for: Deriving a Deployment Threshold for Benefit and Harm in Clinical AI: Multi-Institutional Decision-Analytic Modeling Study
Source: JMIR AI. 2026 Oct 23;5:e109863. doi: 10.2196/109863 (PMC13743961; doi:10.2196/109863)
Supplement: Supplementary file 3 [file ai_v5i1e109863_app3.docx]

**Query-generation robustness and the full generation protocol**

*Paired comparisons under the published and locally generated query sets on the derivation study’s own MTSamples sample, holding documents, configurations and all other settings constant, followed by the generation protocol verbatim.*

**Table S1. Paired comparison of query sets**

| **Query format** | **Configuration** | **Nominal tier** | **baseline_MRR10 gpt4o** | **baseline_MRR10 local** | **delta_MRR10 gpt4o** | **delta_MRR10 local** | **dMRR difference (local - published)** | **rank gpt4o** | **subgroup gpt4o** | **rank local** | **subgroup local** | **assignment agrees** |
| --- | --- | --- | --- | --- | --- | --- | --- | --- | --- | --- | --- | --- |
| keyword | BERT-base-uncased | 2 | 0.2381 | 0.222 | 0.1025 | 0.0459 | -0.0566 | 3 | benefited | 5 | benefited | yes |
| keyword | BGE-base | 1 | 0.9242 | 0.9227 | -0.0324 | -0.0293 | 0.003 | 10 | harmed | 10 | harmed | yes |
| keyword | BioBERT | 2 | 0.429 | 0.2918 | 0.0992 | 0.1271 | 0.0279 | 5 | benefited | 2 | benefited | yes |
| keyword | BioLORD-2023 | 1 | 0.8156 | 0.7846 | -0.0298 | -0.0193 | 0.0105 | 8 | harmed | 9 | harmed | yes |
| keyword | BioMistral-7B | 2 | 0.3433 | 0.3762 | 0.2403 | 0.1709 | -0.0694 | 1 | benefited | 1 | benefited | yes |
| keyword | ClinicalBERT | 2 | 0.4681 | 0.3981 | -0.0316 | -0.04 | -0.0084 | 9 | harmed | 11 | harmed | yes |
| keyword | E5-Mistral-7B | 2 | 0.111 | 0.1463 | 0.1173 | 0.1144 | -0.0029 | 2 | benefited | 4 | benefited | yes |
| keyword | E5-Mistral-7B-ablation | 2 | 0.7438 | 0.7194 | 0.0003 | -0.007 | -0.0073 | 6 | benefited | 6 | harmed | no |
| keyword | GTE-base | 1 | 0.9318 | 0.9298 | -0.0424 | -0.0123 | 0.0302 | 11 | harmed | 8 | harmed | yes |
| keyword | MedCPT | 1 | 0.8808 | 0.8624 | -0.0709 | -0.0966 | -0.0257 | 13 | harmed | 13 | harmed | yes |
| keyword | Nomic-embed-text | 1 | 0.9203 | 0.9319 | -0.0432 | -0.0618 | -0.0186 | 12 | harmed | 12 | harmed | yes |
| keyword | Nomic-embed-text-nopfx | 1 | 0.9061 | 0.9014 | -0.021 | -0.0091 | 0.0119 | 7 | harmed | 7 | harmed | yes |
| keyword | Phi-3-mini | 2 | 0.5478 | 0.4886 | 0.1015 | 0.1251 | 0.0236 | 4 | benefited | 3 | benefited | yes |
| nl | BERT-base-uncased | 2 | 0.2089 | 0.2138 | 0.1683 | 0.1372 | -0.0311 | 4 | benefited | 6 | benefited | yes |
| nl | BGE-base | 1 | 0.9292 | 0.935 | -0.0312 | -0.0131 | 0.0182 | 9 | harmed | 8 | harmed | yes |
| nl | BioBERT | 2 | 0.3439 | 0.3022 | 0.2786 | 0.2655 | -0.0131 | 1 | benefited | 1 | benefited | yes |
| nl | BioLORD-2023 | 1 | 0.8808 | 0.925 | -0.0418 | -0.0522 | -0.0104 | 12 | harmed | 11 | harmed | yes |
| nl | BioMistral-7B | 2 | 0.4763 | 0.4342 | 0.0774 | 0.1341 | 0.0567 | 7 | benefited | 7 | benefited | yes |
| nl | ClinicalBERT | 2 | 0.3286 | 0.2954 | 0.2175 | 0.163 | -0.0545 | 2 | benefited | 4 | benefited | yes |
| nl | E5-Mistral-7B | 2 | 0.0905 | 0.1005 | 0.1407 | 0.1458 | 0.0051 | 6 | benefited | 5 | benefited | yes |
| nl | E5-Mistral-7B-ablation | 2 | 0.5861 | 0.6263 | 0.2162 | 0.2046 | -0.0117 | 3 | benefited | 2 | benefited | yes |
| nl | GTE-base | 1 | 0.9395 | 0.9608 | -0.0394 | -0.0322 | 0.0072 | 11 | harmed | 9 | harmed | yes |
| nl | MedCPT | 1 | 0.8036 | 0.8653 | -0.0531 | -0.0905 | -0.0374 | 13 | harmed | 13 | harmed | yes |
| nl | Nomic-embed-text | 1 | 0.9367 | 0.9275 | -0.033 | -0.0667 | -0.0338 | 10 | harmed | 12 | harmed | yes |
| nl | Nomic-embed-text-nopfx | 1 | 0.9381 | 0.9417 | -0.0254 | -0.0365 | -0.0111 | 8 | harmed | 10 | harmed | yes |
| nl | Phi-3-mini | 2 | 0.5459 | 0.5035 | 0.1427 | 0.1947 | 0.052 | 5 | benefited | 3 | benefited | yes |

**Model and sampling parameters**

Both external corpora are PhysioNet credentialed-access resources under data use agreements that preclude transmission to a commercial API. The metadata-derived query protocol of the derivation study was therefore executed locally. This section records the protocol verbatim so that the substitution and its consequences are auditable.

| **setting** | **value** |
| --- | --- |
| generating model | qwen2.5:14b-instruct, served locally via Ollama |
| temperature | 0.0 for metadata extraction; 0.3 for query generation |
| random seed | none set; generation is not reproducible run to run |
| token budget | 300 extraction; 150 natural language; 60 keyword |
| retries | 3, with exponential backoff |
| extraction window | first 6,000 characters of the note |
| structured output | JSON mode enabled for the extraction stage |
| reasoning | suppressed (think=False) |

A larger local model, qwen3:30b, was tried first and rejected. It emits reasoning regardless of the suppression flag; the reasoning consumed the token budget before any answer, and every extracted field returned "Unknown". This is recorded because the failure is silent: the pipeline completes and writes output.

**Two-stage structure**

Stage 1 extracts structured metadata from the note text. Stage 2 receives only that metadata and never the document text, by design, to limit lexical leakage from document into query. Stage 1 runs on the full note for both document variants, so the query set is identical across variants and only the documents differ.

**Prompts, verbatim**

Stage 1, metadata extraction:

Extract metadata from this clinical note. Return ONLY a JSON object with exactly these keys: "specialty", "note_type", "primary_diagnosis", "secondary_diagnoses", "age", "sex". Use "Unknown" for any field not stated. "secondary_diagnoses" must be a list of strings. No prose, no code fences, JSON only. NOTE: [note text] JSON:

Stage 2a, natural-language query, rendered with example values:

Based ONLY on this metadata, write a natural language clinical question (1-2 sentences): Specialty: Emergency Medicine | Note type: ED Provider Note | Diagnosis: Hypoxia | Other: COPD | Patient: adult Female. Use ONLY the values given. Do not infer, add, or invent any detail not present above, including age. Query:

Stage 2b, keyword query, rendered with example values:

Based ONLY on this metadata, output 3-6 clinical search keywords (space-separated): Specialty: Emergency Medicine | Note type: ED Provider Note | Diagnosis: Hypoxia | Other: COPD | Patient: adult Female. Use ONLY the values given. Do not infer, add, or invent any detail not present above, including age. Keywords:

The two instruction lines and the metadata field line are reproduced from the derivation study's protocol. The sentence beginning "Use ONLY the values given" is the single added instruction and is discussed below.

**Deviations from the published protocol**

Four deviations were required. All are forced by the data governance constraint or by the substituted model's behaviour, and all are stated rather than absorbed.

• Model substitution, required by the data use agreements, and bounded by the paired comparison in Table S1.

• JSON mode on the extraction stage, without which prose leaks into the structured output and parsing fails.

• A no-invention instruction plus demographics normalisation. Without both, the substituted model supplied plausible patient ages absent from the metadata, so that "Female" became "a 45-year-old female", breaching the protocol's guarantee that queries derive only from extracted fields. A missing age is normalised to the protocol's own "adult" default rather than to a number, so no digit is available for the model to anchor on. With both measures, invented ages fell to 2 of 100 for ER-Reason and 1 of 100 for MIMIC-IV-Note.

• Output cleaning: truncation at the first non-ASCII character, implemented as re.search(r"[^\x00-\x7F]", s), because the substituted model occasionally breaks into another language mid-generation; removal of underscores; and dropping a trailing incomplete sentence rather than emitting a fragment. Cleaning is applied inline during generation and only the cleaned strings are written, so raw pre-truncation generations were not retained and the frequency and content of truncation events cannot be reconstructed.

**Output characteristics**

| **measure** | **ER-Reason** | **MIMIC-IV-Note** | **MTSamples** |
| --- | --- | --- | --- |
| JSON parsed | 100/100 | 100/100 | 100/100 |
| primary diagnosis "Unknown" | 0/100 | 0/100 | 4/100 |
| natural-language length, median | 270 characters | 281 characters | 173 characters |
| keyword count, median | 6 | 6 | 6 |
| invented ages | 2/100 | 1/100 | 0/100 |

**Grounding check**

For ER-Reason, extracted primary diagnoses were compared against the corpus registry diagnosis field, which the extraction stage never sees. Token overlap was 45 of 100. Disagreements were inspected and reflected presentation-versus-final-diagnosis differences and vocabulary differences, not fabrication. Two examples follow.

• Extracted "acute abdominal pain, concern for pyelonephritis or intra-abdominal process" against registry "complicated urinary tract infection".

• Extracted "dermatomal vesicular rash, suspected shingles" against registry "ophthalmic herpes zoster".

For MIMIC-IV-Note the corresponding field is the note's own description line, and overlap was 73 of 100. The check is reported, not enforced: a low overlap does not indicate extraction failure when the note records presentation and the registry records a coded final diagnosis.

**Scope of the substitution bound**

The paired comparison in Table S1 was run on MTSamples, a public corpus for which the derivation study's published queries exist. It bounds the substitution where both query sets can be compared. It cannot establish that the external result is unchanged by the substitution, because no published queries exist for the credentialed corpora, which is why the substitution was necessary.
