# Supplementary material for: Deriving a Deployment Threshold for Benefit and Harm in Clinical AI: Multi-Institutional Decision-Analytic Modeling Study
Source: JMIR AI. 2026 Oct 23;5:e109863. doi: 10.2196/109863 (PMC13743961; doi:10.2196/109863)
Supplement: Supplementary file 4 [file ai_v5i1e109863_app4.docx]

**Completed CHEERS 2022 checklist**

*Consolidated Health Economic Evaluation Reporting Standards 2022, all 28 items, with reported status and the manuscript location of each.*

Item wording follows Husereau D, Drummond M, Augustovski F, et al. Consolidated Health Economic Evaluation Reporting Standards 2022 (CHEERS 2022) statement. Value Health. 2022;25(1):3-9. Locations refer to the submitted manuscript section headings.

**Title**

| **No.** | **Item** | **Reported** | **Location** |
| --- | --- | --- | --- |
| 1 | Identify the study as an economic evaluation and specify the interventions being compared. | Partly | Title |

The title identifies the study as a decision-analytic modeling study and names the clinical AI deployment threshold as its subject, but it does not use the term "economic evaluation" or enumerate the three strategies. The strategies and economic-evaluation components are specified in the Abstract and Methods.

**Abstract**

| **No.** | **Item** | **Reported** | **Location** |
| --- | --- | --- | --- |
| 2 | Provide a structured summary that highlights context, key methods, results, and alternative analyses. | Yes | Abstract |

Structured under Background, Objective, Methods, Results, Conclusions. Alternative analyses reported: regularisation sweep, cross-validated effect specification, matched-length whole-note encoding, alternative query generator, and the code-search boundary replication.

**Introduction**

| **No.** | **Item** | **Reported** | **Location** |
| --- | --- | --- | --- |
| 3 | Give the context for the study, the study question, and its practical relevance for decision making in policy or practice. | Yes | Introduction |

**Methods**

| **No.** | **Item** | **Reported** | **Location** |
| --- | --- | --- | --- |
| 4 | Indicate whether a health economic analysis plan was developed and where available. | Yes, none prespecified | Discussion, Strengths and Limitations |
| 5 | Describe characteristics of the study population. | Partly | Methods, Deployment Scale |
| 6 | Describe relevant aspects of the system in which the decision needs to be made. | Yes | Methods, Deployment Scale |
| 7 | Describe the interventions or strategies being compared and why chosen. | Yes | Methods, Decision Problem; Instantiation |
| 8 | State the perspective(s) adopted and why chosen. | Yes | Methods, Decision Problem |
| 9 | State the time horizon and why appropriate. | Yes | Methods, Decision Problem |
| 10 | Report the discount rate(s) and reason chosen. | Yes | Methods, Decision Problem |
| 11 | Describe what outcomes were used as measures of benefit and harm. | Yes | Methods, The General Threshold; Instantiation |
| 12 | Describe how outcomes used to capture benefit and harm were measured. | Yes | Methods, Instantiation; Parameters and Reporting |
| 13 | Describe the population and methods used to measure and value outcomes. | Yes | Methods, Parameters and Reporting |
| 14 | Describe how costs were valued. | Yes | Methods, Parameters and Reporting |
| 15 | Report the dates of the estimated resource quantities and unit costs, plus the currency and year of conversion. | Yes | Methods, Parameters and Reporting |
| 16 | If modelling is used, describe in detail and why used. Report if the model is publicly available and where it can be accessed. | Yes | Methods, Decision Problem through Analysis; Data Availability |
| 17 | Describe any methods for analysing or statistically transforming data, extrapolation, and validation of any model used. | Yes | Methods, Analysis; Results |
| 18 | Describe any methods used for estimating how the results of the study vary for subgroups. | Yes | Methods, Subgroup Prevalence; Screening Thresholds |
| 19 | Describe how impacts are distributed across different individuals or adjustments made to reflect priority populations. | Not applicable | Discussion, Strengths and Limitations |
| 20 | Describe methods to characterise any sources of uncertainty in the analysis. | Yes | Methods, Analysis; Results |
| 21 | Describe any approaches to engage patients or service recipients, the general public, communities, or stakeholders in the design of the study. | Yes, none undertaken | Discussion, Strengths and Limitations |

Item 4. Reported as a negative in Strengths and Limitations: no health economic analysis plan was prespecified or registered. The model, its parameters, and the analysis code are archived and publicly available, which supports reproducibility but does not substitute for a prespecified plan.

Item 5. The unit of analysis is a deployed retrieval system rather than a patient cohort. Population characteristics are described at the level of the health system whose activity determines deployment scale, and at the level of the document corpora used to measure the technical effect. No patient-level characteristics are used in the decision model.

Item 19. Distributional effects across individuals were not modelled. The analysis concerns whether an intervention should be deployed across a system, not how its consequences distribute across patient groups. Stated as a limitation.

Item 21. Reported as a negative in Strengths and Limitations: no patient, public, or stakeholder engagement was undertaken. The study is a secondary analysis of published system descriptions and de-identified document corpora, with no primary data collection.

**Results**

| **No.** | **Item** | **Reported** | **Location** |
| --- | --- | --- | --- |
| 22 | Report all analytic inputs including uncertainty or distributional assumptions. | Yes | Methods, Parameters and Reporting; Multimedia Appendix 5 |
| 23 | Report the mean values for the main categories of costs and outcomes of interest and summarise them in the most appropriate overall measure. | Yes | Results, The Threshold; The Literature Cannot Establish |
| 24 | Describe how uncertainty about analytic judgments, inputs, or projections affect findings. Report the effect of choice of discount rate and time horizon, if applicable. | Yes | Results, Absolute Scale Cancels, but Relative Translation Can Change the Decision; Decision-Relevant Uncertainty Is Local and Asymmetric |
| 25 | Report on any difference patient, public, community, or stakeholder involvement made to the approach or findings of the study. | Not applicable | - |

Item 22. Every parameter is reported with its value, distribution, source, and whether it is empirical, scenario-based, or illustrative, in Multimedia Appendix 5. Three parameters entering the monetary multiplier have no published source and are declared illustrative.

Item 25. No engagement was undertaken, so no difference can be reported. See item 21.

**Discussion**

| **No.** | **Item** | **Reported** | **Location** |
| --- | --- | --- | --- |
| 26 | Report key findings, limitations, ethical or equity considerations not captured, and how these could affect patients, policy, or practice. | Yes | Discussion, Principal Findings; Strengths and Limitations; Conclusions |

**Other relevant information**

| **No.** | **Item** | **Reported** | **Location** |
| --- | --- | --- | --- |
| 27 | Describe how the study was funded and any role of the funder in the identification, design, conduct, and reporting of the analysis. | Yes | Funding |
| 28 | Report authors' conflicts of interest according to journal or International Committee of Medical Journal Editors requirements. | Yes | Conflicts of Interest |

**Summary**

| **Status** | **Items** | **Numbers** |
| --- | --- | --- |
| Reported | 24 | 2, 3, 4, 6, 7, 8, 9, 10, 11, 12, 13, 14, 15, 16, 17, 18, 20, 21, 22, 23, 24, 26, 27, 28 |
| Partly reported | 2 | 1, 5 |
| Not applicable | 2 | 19, 25 |

Items 4 and 21 are reported as negatives rather than left silent: no health economic analysis plan was prespecified, and no patient or stakeholder engagement was undertaken.
