# Supplementary material for: Deriving a Deployment Threshold for Benefit and Harm in Clinical AI: Multi-Institutional Decision-Analytic Modeling Study
Source: JMIR AI. 2026 Oct 23;5:e109863. doi: 10.2196/109863 (PMC13743961; doi:10.2196/109863)
Supplement: Supplementary file 5 [file ai_v5i1e109863_app5.docx]

**Completed CHEERS-AI checklist**

*Consolidated Health Economic Evaluation Reporting Standards for Interventions That Use Artificial Intelligence: the 28 CHEERS 2022 items with 8 AI-specific elaborations, plus the 10 AI extension items, with reported status and manuscript location of each.*

Item wording follows Elvidge J, Hawksworth C, Saygin Avsar T, et al. Consolidated Health Economic Evaluation Reporting Standards for Interventions That Use Artificial Intelligence (CHEERS-AI). Value Health. 2024;27(9):1196-1205. doi:10.1016/j.jval.2024.05.006

CHEERS-AI comprises the 28 CHEERS 2022 items, 8 of which carry AI-specific elaborations, plus 10 new AI-specific extension items (AI 1 to AI 10). Where an item carries an elaboration, the elaboration is assessed separately from the parent item. Locations refer to the submitted manuscript section headings.

**Title**

| **No.** | **Item** | **AI elaboration** | **Status** | **Location** |
| --- | --- | --- | --- | --- |
| 1 | Identify the study as an economic evaluation and specify the interventions being compared. | Indicate that the intervention involves an AI component that is under evaluation. | Partly | Title |

The title identifies clinical AI and the deployment threshold as the subject, and names the design as a decision-analytic modeling study, but it does not use the term "economic evaluation" or enumerate the three strategies. The strategies and economic-evaluation components are specified in the Abstract and Methods.

**Abstract**

| **No.** | **Item** | **AI elaboration** | **Status** | **Location** |
| --- | --- | --- | --- | --- |
| 2 | Provide a structured summary that highlights context, key methods, results, and alternative analysis. | Specify the purpose of the intervention with an AI component, and the AI technique used. | Yes | Abstract |

The abstract states the intervention purpose within the deployment decision problem and names the AI technique evaluated: zero-phase component analysis (ZCA) whitening, a retraining-free correction to clinical retrieval embeddings.

**Introduction**

| **No.** | **Item** | **Status** | **Location** |
| --- | --- | --- | --- |
| 3 | Give the context for the study, the study question, and its practical relevance for decision making in policy or practice. | Yes | Introduction |

**Methods**

| **No.** | **Item** | **AI elaboration** | **Status** | **Location** |
| --- | --- | --- | --- | --- |
| 4 | Indicate whether a health economic analysis plan was developed and where available. | - | Yes, none prespecified | Discussion, Strengths and Limitations |
| 5 | Describe characteristics of the study population. | - | Partly | Methods, Deployment Scale |
| 6 | Provide relevant contextual information that may influence findings. | - | Yes | Methods, Deployment Scale |
| 7 | Describe the interventions or strategies being compared and why chosen. | Describe key details of the AI component (a to h). | Partly | Methods, Decision Problem; Instantiation |
| AI 1 | User autonomy. Indicate whether the AI intervention is directive, or whether the user retains autonomy to make the care decision. | - | Yes | Methods, Instantiation |
| 8 | State the perspective(s) adopted and why chosen. | - | Yes | Methods, Decision Problem |
| 9 | State the time horizon and why appropriate. | - | Yes | Methods, Decision Problem |
| 10 | Report the discount rate(s) and reason chosen. | - | Yes | Methods, Decision Problem |
| 11 | Describe what outcomes were used as measures of benefit and harm. | Describe whether the measure relates to health, diagnostic, process, or other outcomes. | Yes | Methods, The General Threshold; Instantiation |
| 12 | Describe how outcomes used to capture benefit and harm were measured. | For model-based analysis, describe assumptions informing benefit and harm; describe plausibility, citing supportive evidence. | Yes | Methods, Instantiation; Parameters and Reporting |
| AI 2 | Measurement of AI effect. Describe the data sources for the AI intervention's impact on outcomes. | - | Yes | Methods, Instantiation; Results |
| AI 3 | Measurement of AI learning over time. If the AI intervention learns over time, explain how this affects performance and how it was measured. | - | Not applicable, stated | Methods, Instantiation |
| AI 4 | Development of AI component. Describe how the AI component was developed, including training data and how errors and biases were identified, or cite a source. | - | Partly | Methods, Subgroup Prevalence; Multimedia Appendix 1 |
| AI 5 | Validation of AI component. Describe how the AI component and its performance estimates were validated, or cite a source. | - | Yes | Methods, Corpora for the Transport Evaluation; Results |
| AI 6 | Health benefit. Describe how the AI intervention could directly or indirectly provide a health benefit. | - | Yes | Methods, The General Threshold; Instantiation |
| AI 7 | Population differences. Describe important differences between the data sources for the AI intervention's impact and the data set used to develop it. | - | Partly | Methods, Corpora; Discussion, Strengths and Limitations |
| 13 | Describe the population and methods used to measure and value outcomes. | - | Yes | Methods, Parameters and Reporting |
| 14 | Describe how costs were valued. | Describe the purchase cost and what it is composed of; describe additional implementation and maintenance costs. | Yes | Methods, Parameters and Reporting; Multimedia Appendix 5 |
| 15 | Report the dates of the estimated resource quantities and unit costs, plus currency and year of conversion. | - | Yes | Methods, Parameters and Reporting |
| 16 | If modeling is used, describe in detail and why used. Report if the model is publicly available and where. | Describe if the AI component has influenced the choice of health economic model and explain why. | Yes | Methods, Decision Problem; Data Availability |
| 17 | Describe any methods for analyzing or transforming data, extrapolation, and validation of any model used. | - | Yes | Methods, Analysis |
| AI 8 | Modeling of AI learning over time. If the AI intervention learns over time, describe assumptions used to model how learning affects performance. | - | Not applicable, stated | Methods, Instantiation |
| 18 | Describe any methods used for estimating how results vary for subgroups. | - | Yes | Methods, Subgroup Prevalence; Screening Thresholds |
| 19 | Describe how impacts are distributed across individuals or adjustments to reflect priority populations. | - | Not applicable | Discussion, Strengths and Limitations |
| 20 | Describe methods to characterize any sources of uncertainty in the analysis. | - | Yes | Methods, Analysis |
| 21 | Describe any approaches to engage patients, the public, communities, or stakeholders in the design of the study. | - | Yes, none undertaken | Discussion, Strengths and Limitations |

Item 7, AI elaboration (a to h). The AI technique, the locked non-adaptive nature of the correction, its purpose, mechanism, and intended user interaction are reported. The 13 evaluated configurations and the regularisation parameter are identified, but no separate version identifier exists for the intervention. No formal classification by intended purpose or risk tier under the IMDRF Software as a Medical Device or NICE evidence standards frameworks is assigned, and implementation-cost reporting only partly addresses the remaining cost-related detail. The item is therefore marked partly reported. The parent CHEERS 2022 item is fully reported; this partial rating concerns the AI elaboration only.

AI 1, user autonomy. The manuscript states that the intervention informs rather than directs clinical management: the retrieval layer surfaces record content for a clinician who retains the care decision. The adoption parameter represents the probability that retrieved output is acted upon in a decision.

AI 3 and AI 8, learning over time. Not applicable. The intervention is a retraining-free post hoc transformation with fixed parameters, applied to a static embedding space; it does not learn from data collected during use. The CHEERS-AI pilot exercise found no economic evaluation reporting these items, so stating non-applicability explicitly is worthwhile.

AI 4, development of the AI component. Partly, and the gap is the subject of the study rather than an omission in it. For the 13 configurations evaluated, checkpoints and their published sources are identified. For the 55 systems in the extraction frame, only one provided sufficient information for the relevant extraction representation to be inferred; none reported retrieval pooling as such, and training data and bias-identification details were generally unavailable.

AI 7, population differences. Partly. Differences between the corpora used to measure the intervention's effect are characterised in detail, including institution, documentation genre, de-identification regime, and document length. Differences between those corpora and the data used to train the underlying embedding models cannot be characterised, because training corpora are undisclosed for most of the configurations examined. Stated as a limitation.

**Results**

| **No.** | **Item** | **AI elaboration** | **Status** | **Location** |
| --- | --- | --- | --- | --- |
| 22 | Report all analytic inputs including uncertainty or distributional assumptions. | - | Yes | Methods, Parameters and Reporting; Multimedia Appendix 5 |
| 23 | Report the mean values for the main categories of costs and outcomes and summarize them in the most appropriate overall measure. | - | Yes | Results, The Threshold; The Literature Cannot Establish |
| 24 | Describe how uncertainty about analytic judgments, inputs, or projections affect findings. Report the effect of discount rate and time horizon, if applicable. | - | Yes | Results, Absolute Scale Cancels, but Relative Translation Can Change the Decision; Decision-Relevant Uncertainty Is Local and Asymmetric |
| AI 9 | Impact of AI uncertainty. Indicate the extent to which features of the AI intervention may contribute to increased uncertainty about its cost-effectiveness. | - | Yes, central | Results; Discussion, Principal Findings |
| 25 | Report on any difference patient, public, community, or stakeholder involvement made to the approach or findings. | - | Not applicable | - |

AI 9. AI-related uncertainty is the principal subject of the study rather than a reporting requirement discharged in passing. The analysis shows that the response-defining state is not reliably recoverable from published descriptions, that response prevalence remains unidentified without explicit classification assumptions, and that uncertainty in response state, screening performance, and relative benefit-harm translation can alter the deployment decision. No monetary value of perfect information is assigned, because the prevalence distribution such a calculation would require is not identified; decision relevance is reported structurally instead.

**Discussion**

| **No.** | **Item** | **AI elaboration** | **Status** | **Location** |
| --- | --- | --- | --- | --- |
| 26 | Report key findings, limitations, ethical or equity considerations not captured, and how these could affect patients, policy, or practice. | Comment on potential biases associated with the AI intervention, for example algorithmic bias, and implications for generalizability and interpretation. | Yes | Discussion, Principal Findings; Strengths and Limitations; Conclusions |
| AI 10 | Implementation of AI. Comment on any requirements needed to integrate the AI intervention into practice, and other implementation considerations, including implications for interpretation of cost-effectiveness results. | - | Yes | Results, Screening; Observed Screen Accuracy; Discussion |

Item 26, AI elaboration. Findings, limitations, and generalisability are reported at length. The manuscript explicitly distinguishes representational heterogeneity as a deployment risk from algorithmic bias as an equity concern, and states that differential performance or downstream effects across demographic or priority populations were not assessed; fairness and health-equity impacts are therefore not inferred.

AI 10. Implementation requirements are the operational output of the study: the screening design specifies what a site must do before deploying, and the resampling analysis specifies the minimum measurement intensity below which that screen is unreliable.

**Other relevant information**

| **No.** | **Item** | **Status** | **Location** |
| --- | --- | --- | --- |
| 27 | Describe how the study was funded and any role of the funder. | Yes | Funding |
| 28 | Report authors' conflicts of interest per journal or ICMJE requirements. | Yes | Conflicts of Interest |

**Summary**

| **Status** | **Count** | **Items** |
| --- | --- | --- |
| Reported | 29 | 2, 3, 4, 6, 8, 9, 10, 11, 12, 13, 14, 15, 16, 17, 18, 20, 21, 22, 23, 24, 26, 27, 28, AI 1, AI 2, AI 5, AI 6, AI 9, AI 10 |
| Partly reported | 5 | 1, 5, 7, AI 4, AI 7 |
| Not applicable, stated | 4 | 19, 25, AI 3, AI 8 |

Items 4 and 21 are reported as negatives: no health economic analysis plan was prespecified, and no patient or stakeholder engagement was undertaken. Both are stated in Strengths and Limitations rather than passed over.

**Two items that cannot be fully closed**

Item 5, study population. The unit of analysis is a deployed retrieval system rather than a patient cohort. Population characteristics are described at the level of the health system whose activity determines deployment scale, and at the level of the document corpora used to measure the technical effect. No patient-level characteristics enter the decision model. This is a genuine mismatch between the study design and what the item assumes, not an omission.

Item AI 7, population differences. Differences between the corpora used to measure the intervention's effect are characterised in detail. Differences between those corpora and the data used to train the underlying embedding models cannot be characterised, because training corpora are undisclosed for most configurations examined. This is the study's own finding rather than a reporting failure, and is stated as a limitation.
