# Supplementary material for: Deriving a Deployment Threshold for Benefit and Harm in Clinical AI: Multi-Institutional Decision-Analytic Modeling Study
Source: JMIR AI. 2026 Oct 23;5:e109863. doi: 10.2196/109863 (PMC13743961; doi:10.2196/109863)
Supplement: Supplementary file 6 [file ai_v5i1e109863_app6.docx]

**Decision-model parameters, regularisation sweep, and per-condition effects**

*Every parameter with its value, distribution or range, source, and whether it is empirical, scenario-based, or illustrative; the regularisation sweep with response-defined grouping at each value; and the per-condition effect estimates from which the group means are computed.*

**Table S1. Model parameters**

| **Symbol** | **Definition** | **Base value** | **Distribution or range** | **Units** | **Source** | **Status** | **Enters** |
| --- | --- | --- | --- | --- | --- | --- | --- |
| d_ben | Mean dMRR@10, response-defined benefited group (6 configurations) | +0.075745 | SE 0.0259 across configurations | unitless | Companion study, full-corpus protocol at eps=1e-5; grouped by measured sign, not nominal tier | empirical | technical effect |
| d_harm | Mean dMRR@10, response-defined harmed group (7 configurations) | -0.0766 | SE 0.0131 across configurations | unitless | Companion study, full-corpus protocol at eps=1e-5; grouped by measured sign, not nominal tier | empirical | technical effect |
| p* | Universal-deployment threshold | 0.50 | 0.32-0.70 across the regularisation sweep (structural, not a sampling interval) | proportion | Derived from d_ben, d_harm, K/M | derived | threshold |
| r | M_ben / M_harm, relative downstream translation | 1.0 | 0.25 to 4 in structural sensitivity | ratio | No published source; r=1 is an assumption | ILLUSTRATIVE | structural |
| p | Response-defined benefited-state prevalence | not estimated | 0-1 with no classification assumption | proportion | 55-system extraction frame; Appendix 1 | unidentified without classification assumptions | technical effect |
| se | Screen sensitivity | derived | by scored-condition breadth; separately by document-resampling design | proportion | Between-condition variability; resampling, Appendix 3 | empirical | technical effect |
| sp | Screen specificity | derived | by scored-condition breadth; separately by document-resampling design | proportion | Between-condition variability; resampling, Appendix 3 | empirical | technical effect |
| alpha | Technical metric to decision accuracy | 0.70 | TruncN(0.70, 0.15) | unitless | No published source | ILLUSTRATIVE | enters M |
| adoption | Retrieval output adopted into decision | 0.60 | Beta(9, 6) | proportion | No published source | ILLUSTRATIVE | enters M |
| p_event | Adverse event \| adopted erroneous retrieval | 0.12 | Beta(6, 44) | proportion | No published source | ILLUSTRATIVE | enters M |
| C_event | Treatment cost per adverse event | EUR 11,494 | Gamma(2.5, 4597) | EUR | SAMDATA 2024 bed-day NOK 26,153 x 5.11 excess days (Hoogervorst-Schilp 2015) = NOK 133,642, converted at FX = 11.6276 NOK/EUR, the Norges Bank annual average for 2024 (accessed September 19, 2026). Exceeds the European estimates of Durand 2024 (EUR 6,000-10,000 per hospitalisation) and Laroche 2025 (EUR 5,974 mean, EUR 618-27,380, 2023 tariffs); both are carried in the cost sensitivity analysis (Multimedia Appendix 9), across which the threshold is unchanged at 0.50 | empirical | enters M via V |
| Q_event | Utility loss per adverse event | 0.0015 QALY | Gamma(1.5, 0.001); 0.028 alternative | QALY | Disutility 0.09-0.145 applied over the 5.11-day excess stay; Kirwan 2023 alternative | scenario | enters M via V |
| lambda | Willingness to pay | NOK 275,000 | to NOK 825,000 severity-weighted | NOK/QALY | Meld. St. 34 (2015-2016). Applied at nominal policy value without indexation; the Norwegian Directorate for Medical Products reviewed the thresholds in 2026 and recommended no interim adjustment | empirical | enters M via V |
| N | Annual retrievals | 133,000 | fixed | count | approximately 1/20 of 2,656,857 Norwegian somatic specialist patients (2024, Statistics Norway) | empirical | enters M |
| D | Discount factor, 5 years at 4% | 4.4518 | fixed | unitless | Standard | empirical | enters M |
| K_impl | Implementation cost | EUR 800 | Gamma(2, 400) | EUR | Scenario | scenario | enters K |
| K_annual | Annual maintenance | EUR 150 | Gamma(1.5, 100) | EUR | Scenario | scenario | enters K |
| K_screen | Screening cost per condition | EUR 400 x n_cond | Gamma(2, 200 x n_cond) | EUR | Scenario | scenario | enters K |
| FX | Exchange rate | 11.6276 | fixed | NOK/EUR | Norges Bank annual average for 2024, the price year of the SAMDATA bed-day estimate; accessed September 19, 2026 | empirical | enters M via V |
| S | Fixed screening cost | EUR 400 x n_cond | = K_screen | EUR | Incurred whichever way the screen reads; EUR 1,600 at n_cond = 4 | scenario | enters the screening threshold as s = S/M |
| T | Deployment cost | EUR 1,468 | K_impl + K_annual x D | EUR | Incurred only on the systems the screen selects, at a rate that depends on the site prior probability of benefit. S and T are carried separately rather than combined into a single screen-and-treat cost, because combining them would require assuming a value for p | scenario | enters the screening threshold as t = T/M |
| epsilon | ZCA regularisation | 1e-5 | 1e-7 to 1e-2 swept | unitless | Companion study default | empirical | technical effect |

**Table S2. Regularisation sweep under response-defined grouping**

*Group membership is determined at each value by the sign of the measured mean effect, so group sizes change across the sweep. This is the structural sensitivity reported in place of a sampling interval on the threshold.*

| **epsilon** | **n_benefited** | **n_harmed** | **d_ben** | **d_harm** | **p_star** |
| --- | --- | --- | --- | --- | --- |
| 1e-07 | 4 | 9 | 0.04475 | -0.1041 | 0.6993 |
| 1e-06 | 6 | 7 | 0.0585 | -0.0831 | 0.587 |
| 1e-05 | 6 | 7 | 0.0757 | -0.0766 | 0.5028 |
| 1e-04 | 6 | 7 | 0.0990 | -0.0639 | 0.3925 |
| 1e-03 | 7 | 6 | 0.0735 | -0.0340 | 0.3163 |
| 1e-02 | 5 | 8 | 0.0188 | -0.0141 | 0.4288 |

**Table S3. Per-condition effect estimates**

| **model** | **corpus** | **query_format** | **epsilon** | **baseline_MRR@10** | **corpus_only_ZCA_MRR@10** | **dMRR@10** | **Recall@10** |
| --- | --- | --- | --- | --- | --- | --- | --- |
| BioBERT | MTSamples | keyword | 1e-07 | 0.4240 | 0.4978 | 0.0739 | 0.71 |
| BioBERT | MTSamples | keyword | 1e-06 | 0.4240 | 0.4980 | 0.0740 | 0.71 |
| BioBERT | MTSamples | keyword | 1e-05 | 0.4240 | 0.5279 | 0.1039 | 0.73 |
| BioBERT | MTSamples | keyword | 0.0001 | 0.4240 | 0.5855 | 0.1615 | 0.8 |
| BioBERT | MTSamples | keyword | 0.001 | 0.4240 | 0.5191 | 0.0952 | 0.78 |
| BioBERT | MTSamples | keyword | 0.01 | 0.4240 | 0.3529 | -0.0710 | 0.64 |
| BioBERT | MTSamples | natural_language | 1e-07 | 0.3439 | 0.5993 | 0.2554 | 0.77 |
| BioBERT | MTSamples | natural_language | 1e-06 | 0.3439 | 0.5981 | 0.2542 | 0.77 |
| BioBERT | MTSamples | natural_language | 1e-05 | 0.3439 | 0.6216 | 0.2777 | 0.78 |
| BioBERT | MTSamples | natural_language | 0.0001 | 0.3439 | 0.6022 | 0.2583 | 0.82 |
| BioBERT | MTSamples | natural_language | 0.001 | 0.3439 | 0.4600 | 0.1161 | 0.76 |
| BioBERT | MTSamples | natural_language | 0.01 | 0.3439 | 0.3080 | -0.0359 | 0.65 |
| BioBERT | PMC-Patients | keyword | 1e-07 | 0.4217 | 0.4386 | 0.0169 | 0.75 |
| BioBERT | PMC-Patients | keyword | 1e-06 | 0.4217 | 0.4411 | 0.0194 | 0.75 |
| BioBERT | PMC-Patients | keyword | 1e-05 | 0.4217 | 0.4673 | 0.0456 | 0.78 |
| BioBERT | PMC-Patients | keyword | 0.0001 | 0.4217 | 0.5724 | 0.1507 | 0.83 |
| BioBERT | PMC-Patients | keyword | 0.001 | 0.4217 | 0.5682 | 0.1465 | 0.8 |
| BioBERT | PMC-Patients | keyword | 0.01 | 0.4217 | 0.4293 | 0.0076 | 0.69 |
| BioBERT | PMC-Patients | natural_language | 1e-07 | 0.4452 | 0.5838 | 0.1386 | 0.81 |
| BioBERT | PMC-Patients | natural_language | 1e-06 | 0.4452 | 0.5841 | 0.1389 | 0.81 |
| BioBERT | PMC-Patients | natural_language | 1e-05 | 0.4452 | 0.6074 | 0.1622 | 0.84 |
| BioBERT | PMC-Patients | natural_language | 0.0001 | 0.4452 | 0.6245 | 0.1794 | 0.85 |
| BioBERT | PMC-Patients | natural_language | 0.001 | 0.4452 | 0.5401 | 0.0949 | 0.81 |
| BioBERT | PMC-Patients | natural_language | 0.01 | 0.4452 | 0.3928 | -0.0524 | 0.71 |
| BioBERT | Synthetic | keyword | 1e-07 | 0.4514 | 0.2416 | -0.2098 | 0.54 |
| BioBERT | Synthetic | keyword | 1e-06 | 0.4514 | 0.2429 | -0.2085 | 0.55 |
| BioBERT | Synthetic | keyword | 1e-05 | 0.4514 | 0.2712 | -0.1803 | 0.57 |
| BioBERT | Synthetic | keyword | 0.0001 | 0.4514 | 0.3804 | -0.0711 | 0.64 |
| BioBERT | Synthetic | keyword | 0.001 | 0.4514 | 0.4313 | -0.0201 | 0.71 |
| BioBERT | Synthetic | keyword | 0.01 | 0.4514 | 0.4449 | -0.0065 | 0.75 |
| BioBERT | Synthetic | natural_language | 1e-07 | 0.2904 | 0.2237 | -0.0667 | 0.4 |
| BioBERT | Synthetic | natural_language | 1e-06 | 0.2904 | 0.2271 | -0.0633 | 0.4 |
| BioBERT | Synthetic | natural_language | 1e-05 | 0.2904 | 0.2601 | -0.0303 | 0.48 |
| BioBERT | Synthetic | natural_language | 0.0001 | 0.2904 | 0.3451 | 0.0547 | 0.57 |
| BioBERT | Synthetic | natural_language | 0.001 | 0.2904 | 0.3259 | 0.0355 | 0.59 |
| BioBERT | Synthetic | natural_language | 0.01 | 0.2904 | 0.3000 | 0.0096 | 0.53 |
| ClinicalBERT | MTSamples | keyword | 1e-07 | 0.1842 | 0.2428 | 0.0586 | 0.44 |
| ClinicalBERT | MTSamples | keyword | 1e-06 | 0.1842 | 0.2438 | 0.0596 | 0.45 |
| ClinicalBERT | MTSamples | keyword | 1e-05 | 0.1842 | 0.2564 | 0.0721 | 0.47 |
| ClinicalBERT | MTSamples | keyword | 0.0001 | 0.1842 | 0.3020 | 0.1177 | 0.51 |
| ClinicalBERT | MTSamples | keyword | 0.001 | 0.1842 | 0.2942 | 0.1100 | 0.56 |
| ClinicalBERT | MTSamples | keyword | 0.01 | 0.1842 | 0.1959 | 0.0117 | 0.47 |
| ClinicalBERT | MTSamples | natural_language | 1e-07 | 0.2734 | 0.4882 | 0.2148 | 0.72 |
| ClinicalBERT | MTSamples | natural_language | 1e-06 | 0.2734 | 0.4868 | 0.2134 | 0.72 |
| ClinicalBERT | MTSamples | natural_language | 1e-05 | 0.2734 | 0.5124 | 0.2390 | 0.75 |
| ClinicalBERT | MTSamples | natural_language | 0.0001 | 0.2734 | 0.5498 | 0.2764 | 0.76 |
| ClinicalBERT | MTSamples | natural_language | 0.001 | 0.2734 | 0.4435 | 0.1701 | 0.8 |
| ClinicalBERT | MTSamples | natural_language | 0.01 | 0.2734 | 0.2588 | -0.0146 | 0.66 |
| ClinicalBERT | PMC-Patients | keyword | 1e-07 | 0.1354 | 0.1623 | 0.0268 | 0.35 |
| ClinicalBERT | PMC-Patients | keyword | 1e-06 | 0.1354 | 0.1631 | 0.0277 | 0.35 |
| ClinicalBERT | PMC-Patients | keyword | 1e-05 | 0.1354 | 0.1659 | 0.0305 | 0.37 |
| ClinicalBERT | PMC-Patients | keyword | 0.0001 | 0.1354 | 0.1908 | 0.0554 | 0.47 |
| ClinicalBERT | PMC-Patients | keyword | 0.001 | 0.1354 | 0.2333 | 0.0979 | 0.52 |
| ClinicalBERT | PMC-Patients | keyword | 0.01 | 0.1354 | 0.1585 | 0.0231 | 0.36 |
| ClinicalBERT | PMC-Patients | natural_language | 1e-07 | 0.2570 | 0.4085 | 0.1515 | 0.63 |
| ClinicalBERT | PMC-Patients | natural_language | 1e-06 | 0.2570 | 0.4104 | 0.1534 | 0.63 |
| ClinicalBERT | PMC-Patients | natural_language | 1e-05 | 0.2570 | 0.4228 | 0.1658 | 0.65 |
| ClinicalBERT | PMC-Patients | natural_language | 0.0001 | 0.2570 | 0.4698 | 0.2128 | 0.7 |
| ClinicalBERT | PMC-Patients | natural_language | 0.001 | 0.2570 | 0.4188 | 0.1618 | 0.69 |
| ClinicalBERT | PMC-Patients | natural_language | 0.01 | 0.2570 | 0.2784 | 0.0213 | 0.52 |
| ClinicalBERT | Synthetic | keyword | 1e-07 | 0.2107 | 0.1622 | -0.0485 | 0.37 |
| ClinicalBERT | Synthetic | keyword | 1e-06 | 0.2107 | 0.1623 | -0.0484 | 0.37 |
| ClinicalBERT | Synthetic | keyword | 1e-05 | 0.2107 | 0.1697 | -0.0411 | 0.37 |
| ClinicalBERT | Synthetic | keyword | 0.0001 | 0.2107 | 0.2218 | 0.0110 | 0.42 |
| ClinicalBERT | Synthetic | keyword | 0.001 | 0.2107 | 0.2236 | 0.0129 | 0.5 |
| ClinicalBERT | Synthetic | keyword | 0.01 | 0.2107 | 0.2049 | -0.0058 | 0.52 |
| ClinicalBERT | Synthetic | natural_language | 1e-07 | 0.3160 | 0.1719 | -0.1441 | 0.38 |
| ClinicalBERT | Synthetic | natural_language | 1e-06 | 0.3160 | 0.1714 | -0.1446 | 0.38 |
| ClinicalBERT | Synthetic | natural_language | 1e-05 | 0.3160 | 0.1673 | -0.1487 | 0.38 |
| ClinicalBERT | Synthetic | natural_language | 0.0001 | 0.3160 | 0.1927 | -0.1233 | 0.42 |
| ClinicalBERT | Synthetic | natural_language | 0.001 | 0.3160 | 0.2661 | -0.0500 | 0.57 |
| ClinicalBERT | Synthetic | natural_language | 0.01 | 0.3160 | 0.2916 | -0.0244 | 0.62 |
| BioLORD-2023 | MTSamples | keyword | 1e-07 | 0.8156 | 0.7789 | -0.0367 | 0.91 |
| BioLORD-2023 | MTSamples | keyword | 1e-06 | 0.8156 | 0.7791 | -0.0365 | 0.91 |
| BioLORD-2023 | MTSamples | keyword | 1e-05 | 0.8156 | 0.7858 | -0.0298 | 0.91 |
| BioLORD-2023 | MTSamples | keyword | 0.0001 | 0.8156 | 0.7780 | -0.0376 | 0.92 |
| BioLORD-2023 | MTSamples | keyword | 0.001 | 0.8156 | 0.8065 | -0.0091 | 0.92 |
| BioLORD-2023 | MTSamples | keyword | 0.01 | 0.8156 | 0.80875 | -0.0068 | 0.97 |
| BioLORD-2023 | MTSamples | natural_language | 1e-07 | 0.8808 | 0.8389 | -0.0418 | 0.9 |
| BioLORD-2023 | MTSamples | natural_language | 1e-06 | 0.8808 | 0.8391 | -0.0417 | 0.9 |
| BioLORD-2023 | MTSamples | natural_language | 1e-05 | 0.8808 | 0.8389 | -0.0418 | 0.9 |
| BioLORD-2023 | MTSamples | natural_language | 0.0001 | 0.8808 | 0.8411 | -0.0397 | 0.9 |
| BioLORD-2023 | MTSamples | natural_language | 0.001 | 0.8808 | 0.8581 | -0.0227 | 0.93 |
| BioLORD-2023 | MTSamples | natural_language | 0.01 | 0.8808 | 0.8761 | -0.0047 | 0.97 |
| BioLORD-2023 | PMC-Patients | keyword | 1e-07 | 0.8969 | 0.8598 | -0.0372 | 0.93 |
| BioLORD-2023 | PMC-Patients | keyword | 1e-06 | 0.8969 | 0.8598 | -0.0372 | 0.93 |
| BioLORD-2023 | PMC-Patients | keyword | 1e-05 | 0.8969 | 0.8603 | -0.0367 | 0.93 |
| BioLORD-2023 | PMC-Patients | keyword | 0.0001 | 0.8969 | 0.8631 | -0.0338 | 0.93 |
| BioLORD-2023 | PMC-Patients | keyword | 0.001 | 0.8969 | 0.8777 | -0.0192 | 0.96 |
| BioLORD-2023 | PMC-Patients | keyword | 0.01 | 0.8969 | 0.8903 | -0.0066 | 0.97 |
| BioLORD-2023 | PMC-Patients | natural_language | 1e-07 | 0.9817 | 0.9433 | -0.0383 | 0.97 |
| BioLORD-2023 | PMC-Patients | natural_language | 1e-06 | 0.9817 | 0.9433 | -0.0383 | 0.97 |
| BioLORD-2023 | PMC-Patients | natural_language | 1e-05 | 0.9817 | 0.9433 | -0.0383 | 0.97 |
| BioLORD-2023 | PMC-Patients | natural_language | 0.0001 | 0.9817 | 0.9506 | -0.0311 | 0.99 |
| BioLORD-2023 | PMC-Patients | natural_language | 0.001 | 0.9817 | 0.9531 | -0.0286 | 1.0 |
| BioLORD-2023 | PMC-Patients | natural_language | 0.01 | 0.9817 | 0.9479 | -0.0337 | 1.0 |
| BioLORD-2023 | Synthetic | keyword | 1e-07 | 0.5661 | 0.4470 | -0.1192 | 0.7 |
| BioLORD-2023 | Synthetic | keyword | 1e-06 | 0.5661 | 0.4421 | -0.1241 | 0.7 |
| BioLORD-2023 | Synthetic | keyword | 1e-05 | 0.5661 | 0.4532 | -0.1129 | 0.7 |
| BioLORD-2023 | Synthetic | keyword | 0.0001 | 0.5661 | 0.4582 | -0.1079 | 0.72 |
| BioLORD-2023 | Synthetic | keyword | 0.001 | 0.5661 | 0.5258 | -0.0403 | 0.77 |
| BioLORD-2023 | Synthetic | keyword | 0.01 | 0.5661 | 0.5790 | 0.0129 | 0.83 |
| BioLORD-2023 | Synthetic | natural_language | 1e-07 | 0.6482 | 0.4363 | -0.2119 | 0.72 |
| BioLORD-2023 | Synthetic | natural_language | 1e-06 | 0.6482 | 0.4358 | -0.2124 | 0.72 |
| BioLORD-2023 | Synthetic | natural_language | 1e-05 | 0.6482 | 0.4365 | -0.2117 | 0.72 |
| BioLORD-2023 | Synthetic | natural_language | 0.0001 | 0.6482 | 0.4513 | -0.1969 | 0.73 |
| BioLORD-2023 | Synthetic | natural_language | 0.001 | 0.6482 | 0.5474 | -0.1008 | 0.8 |
| BioLORD-2023 | Synthetic | natural_language | 0.01 | 0.6482 | 0.6195 | -0.0287 | 0.85 |
| MedCPT | MTSamples | keyword | 1e-07 | 0.8758 | 0.8088 | -0.0671 | 0.92 |
| MedCPT | MTSamples | keyword | 1e-06 | 0.8758 | 0.8088 | -0.0671 | 0.92 |
| MedCPT | MTSamples | keyword | 1e-05 | 0.8758 | 0.8096 | -0.0662 | 0.92 |
| MedCPT | MTSamples | keyword | 0.0001 | 0.8758 | 0.8171 | -0.0587 | 0.93 |
| MedCPT | MTSamples | keyword | 0.001 | 0.8758 | 0.8670 | -0.0088 | 0.95 |
| MedCPT | MTSamples | keyword | 0.01 | 0.8758 | 0.8739 | -0.0019 | 0.96 |
| MedCPT | MTSamples | natural_language | 1e-07 | 0.7986 | 0.7449 | -0.0538 | 0.89 |
| MedCPT | MTSamples | natural_language | 1e-06 | 0.7986 | 0.7450 | -0.0536 | 0.89 |
| MedCPT | MTSamples | natural_language | 1e-05 | 0.7986 | 0.7500 | -0.0486 | 0.89 |
| MedCPT | MTSamples | natural_language | 0.0001 | 0.7986 | 0.7721 | -0.0265 | 0.89 |
| MedCPT | MTSamples | natural_language | 0.001 | 0.7986 | 0.8119 | 0.0133 | 0.92 |
| MedCPT | MTSamples | natural_language | 0.01 | 0.7986 | 0.8375 | 0.0388 | 0.94 |
| MedCPT | PMC-Patients | keyword | 1e-07 | 0.9267 | 0.8154 | -0.1113 | 0.95 |
| MedCPT | PMC-Patients | keyword | 1e-06 | 0.9267 | 0.8154 | -0.1113 | 0.95 |
| MedCPT | PMC-Patients | keyword | 1e-05 | 0.9267 | 0.8154 | -0.1113 | 0.95 |
| MedCPT | PMC-Patients | keyword | 0.0001 | 0.9267 | 0.8255 | -0.1011 | 0.95 |
| MedCPT | PMC-Patients | keyword | 0.001 | 0.9267 | 0.8979 | -0.0288 | 0.99 |
| MedCPT | PMC-Patients | keyword | 0.01 | 0.9267 | 0.9231 | -0.0036 | 1.0 |
| MedCPT | PMC-Patients | natural_language | 1e-07 | 0.8839 | 0.8205 | -0.0634 | 0.93 |
| MedCPT | PMC-Patients | natural_language | 1e-06 | 0.8839 | 0.8205 | -0.0634 | 0.93 |
| MedCPT | PMC-Patients | natural_language | 1e-05 | 0.8839 | 0.8255 | -0.0584 | 0.93 |
| MedCPT | PMC-Patients | natural_language | 0.0001 | 0.8839 | 0.8301 | -0.0538 | 0.93 |
| MedCPT | PMC-Patients | natural_language | 0.001 | 0.8839 | 0.8734 | -0.0105 | 0.97 |
| MedCPT | PMC-Patients | natural_language | 0.01 | 0.8839 | 0.8927 | 0.0088 | 1.0 |
| MedCPT | Synthetic | keyword | 1e-07 | 0.7088 | 0.4532 | -0.2555 | 0.75 |
| MedCPT | Synthetic | keyword | 1e-06 | 0.7088 | 0.4557 | -0.2530 | 0.75 |
| MedCPT | Synthetic | keyword | 1e-05 | 0.7088 | 0.4536 | -0.2552 | 0.75 |
| MedCPT | Synthetic | keyword | 0.0001 | 0.7088 | 0.4922 | -0.2166 | 0.8 |
| MedCPT | Synthetic | keyword | 0.001 | 0.7088 | 0.6070 | -0.1018 | 0.92 |
| MedCPT | Synthetic | keyword | 0.01 | 0.7088 | 0.6674 | -0.0414 | 0.93 |
| MedCPT | Synthetic | natural_language | 1e-07 | 0.7004 | 0.4205 | -0.2799 | 0.7 |
| MedCPT | Synthetic | natural_language | 1e-06 | 0.7004 | 0.4205 | -0.2798 | 0.7 |
| MedCPT | Synthetic | natural_language | 1e-05 | 0.7004 | 0.4326 | -0.2678 | 0.7 |
| MedCPT | Synthetic | natural_language | 0.0001 | 0.7004 | 0.4473 | -0.2531 | 0.72 |
| MedCPT | Synthetic | natural_language | 0.001 | 0.7004 | 0.5422 | -0.1582 | 0.82 |
| MedCPT | Synthetic | natural_language | 0.01 | 0.7004 | 0.6005 | -0.0999 | 0.89 |
| BGE-base | MTSamples | keyword | 1e-07 | 0.9242 | 0.8918 | -0.0324 | 0.96 |
| BGE-base | MTSamples | keyword | 1e-06 | 0.9242 | 0.8918 | -0.0324 | 0.96 |
| BGE-base | MTSamples | keyword | 1e-05 | 0.9242 | 0.8918 | -0.0324 | 0.96 |
| BGE-base | MTSamples | keyword | 0.0001 | 0.9242 | 0.8924 | -0.0318 | 0.96 |
| BGE-base | MTSamples | keyword | 0.001 | 0.9242 | 0.9107 | -0.0135 | 0.97 |
| BGE-base | MTSamples | keyword | 0.01 | 0.9242 | 0.9158 | -0.0083 | 0.98 |
| BGE-base | MTSamples | natural_language | 1e-07 | 0.9192 | 0.8879 | -0.0312 | 0.94 |
| BGE-base | MTSamples | natural_language | 1e-06 | 0.9192 | 0.8924 | -0.0267 | 0.94 |
| BGE-base | MTSamples | natural_language | 1e-05 | 0.9192 | 0.8879 | -0.0312 | 0.94 |
| BGE-base | MTSamples | natural_language | 0.0001 | 0.9192 | 0.8943 | -0.0248 | 0.94 |
| BGE-base | MTSamples | natural_language | 0.001 | 0.9192 | 0.9066 | -0.0126 | 0.95 |
| BGE-base | MTSamples | natural_language | 0.01 | 0.9192 | 0.9360 | 0.0169 | 0.98 |
| BGE-base | PMC-Patients | keyword | 1e-07 | 0.95625 | 0.9228 | -0.0335 | 0.98 |
| BGE-base | PMC-Patients | keyword | 1e-06 | 0.95625 | 0.9228 | -0.0335 | 0.98 |
| BGE-base | PMC-Patients | keyword | 1e-05 | 0.95625 | 0.9228 | -0.0335 | 0.98 |
| BGE-base | PMC-Patients | keyword | 0.0001 | 0.95625 | 0.9244 | -0.0318 | 0.98 |
| BGE-base | PMC-Patients | keyword | 0.001 | 0.95625 | 0.9481 | -0.0082 | 0.98 |
| BGE-base | PMC-Patients | keyword | 0.01 | 0.95625 | 0.9558 | -0.0004 | 0.99 |
| BGE-base | PMC-Patients | natural_language | 1e-07 | 0.9683 | 0.9433 | -0.0250 | 0.98 |
| BGE-base | PMC-Patients | natural_language | 1e-06 | 0.9683 | 0.9433 | -0.0250 | 0.98 |
| BGE-base | PMC-Patients | natural_language | 1e-05 | 0.9683 | 0.9433 | -0.0250 | 0.98 |
| BGE-base | PMC-Patients | natural_language | 0.0001 | 0.9683 | 0.9437 | -0.0247 | 0.98 |
| BGE-base | PMC-Patients | natural_language | 0.001 | 0.9683 | 0.955 | -0.0133 | 0.99 |
| BGE-base | PMC-Patients | natural_language | 0.01 | 0.9683 | 0.9686 | 0.0003 | 1.0 |
| BGE-base | Synthetic | keyword | 1e-07 | 0.7283 | 0.5805 | -0.1479 | 0.77 |
| BGE-base | Synthetic | keyword | 1e-06 | 0.7283 | 0.5821 | -0.1462 | 0.77 |
| BGE-base | Synthetic | keyword | 1e-05 | 0.7283 | 0.5805 | -0.1479 | 0.77 |
| BGE-base | Synthetic | keyword | 0.0001 | 0.7283 | 0.6141 | -0.1142 | 0.82 |
| BGE-base | Synthetic | keyword | 0.001 | 0.7283 | 0.6810 | -0.0474 | 0.88 |
| BGE-base | Synthetic | keyword | 0.01 | 0.7283 | 0.7075 | -0.0208 | 0.92 |
| BGE-base | Synthetic | natural_language | 1e-07 | 0.7355 | 0.5593 | -0.1762 | 0.81 |
| BGE-base | Synthetic | natural_language | 1e-06 | 0.7355 | 0.5593 | -0.1762 | 0.81 |
| BGE-base | Synthetic | natural_language | 1e-05 | 0.7355 | 0.5668 | -0.1687 | 0.81 |
| BGE-base | Synthetic | natural_language | 0.0001 | 0.7355 | 0.6055 | -0.1299 | 0.84 |
| BGE-base | Synthetic | natural_language | 0.001 | 0.7355 | 0.6747 | -0.0608 | 0.86 |
| BGE-base | Synthetic | natural_language | 0.01 | 0.7355 | 0.7021 | -0.0334 | 0.89 |
| GTE-base | MTSamples | keyword | 1e-07 | 0.9268 | 0.6782 | -0.2486 | 0.93 |
| GTE-base | MTSamples | keyword | 1e-06 | 0.9268 | 0.8892 | -0.0376 | 0.94 |
| GTE-base | MTSamples | keyword | 1e-05 | 0.9268 | 0.8893 | -0.0374 | 0.94 |
| GTE-base | MTSamples | keyword | 0.0001 | 0.9268 | 0.8912 | -0.0356 | 0.94 |
| GTE-base | MTSamples | keyword | 0.001 | 0.9268 | 0.8979 | -0.0289 | 0.98 |
| GTE-base | MTSamples | keyword | 0.01 | 0.9268 | 0.9122 | -0.0146 | 1.0 |
| GTE-base | MTSamples | natural_language | 1e-07 | 0.9345 | 0.7410 | -0.1935 | 0.95 |
| GTE-base | MTSamples | natural_language | 1e-06 | 0.9345 | 0.8999 | -0.0346 | 0.96 |
| GTE-base | MTSamples | natural_language | 1e-05 | 0.9345 | 0.9001 | -0.0344 | 0.96 |
| GTE-base | MTSamples | natural_language | 0.0001 | 0.9345 | 0.9056 | -0.0289 | 0.96 |
| GTE-base | MTSamples | natural_language | 0.001 | 0.9345 | 0.9194 | -0.0151 | 0.97 |
| GTE-base | MTSamples | natural_language | 0.01 | 0.9345 | 0.922 | -0.0125 | 0.98 |
| GTE-base | PMC-Patients | keyword | 1e-07 | 0.9817 | 0.9433 | -0.0383 | 0.97 |
| GTE-base | PMC-Patients | keyword | 1e-06 | 0.9817 | 0.9433 | -0.0383 | 0.97 |
| GTE-base | PMC-Patients | keyword | 1e-05 | 0.9817 | 0.9433 | -0.0383 | 0.97 |
| GTE-base | PMC-Patients | keyword | 0.0001 | 0.9817 | 0.9506 | -0.0311 | 0.99 |
| GTE-base | PMC-Patients | keyword | 0.001 | 0.9817 | 0.9637 | -0.0180 | 0.99 |
| GTE-base | PMC-Patients | keyword | 0.01 | 0.9817 | 0.9745 | -0.0072 | 1.0 |
| GTE-base | PMC-Patients | natural_language | 1e-07 | 0.985 | 0.9758 | -0.0092 | 1.0 |
| GTE-base | PMC-Patients | natural_language | 1e-06 | 0.985 | 0.9758 | -0.0092 | 1.0 |
| GTE-base | PMC-Patients | natural_language | 1e-05 | 0.985 | 0.9758 | -0.0092 | 1.0 |
| GTE-base | PMC-Patients | natural_language | 0.0001 | 0.985 | 0.9775 | -0.0075 | 1.0 |
| GTE-base | PMC-Patients | natural_language | 0.001 | 0.985 | 0.9833 | -0.0017 | 1.0 |
| GTE-base | PMC-Patients | natural_language | 0.01 | 0.985 | 0.9833 | -0.0017 | 1.0 |
| GTE-base | Synthetic | keyword | 1e-07 | 0.7276 | 0.2048 | -0.5228 | 0.75 |
| GTE-base | Synthetic | keyword | 1e-06 | 0.7276 | 0.4919 | -0.2357 | 0.79 |
| GTE-base | Synthetic | keyword | 1e-05 | 0.7276 | 0.5869 | -0.1407 | 0.83 |
| GTE-base | Synthetic | keyword | 0.0001 | 0.7276 | 0.6034 | -0.1242 | 0.85 |
| GTE-base | Synthetic | keyword | 0.001 | 0.7276 | 0.6687 | -0.0589 | 0.88 |
| GTE-base | Synthetic | keyword | 0.01 | 0.7276 | 0.7106 | -0.0170 | 0.91 |
| GTE-base | Synthetic | natural_language | 1e-07 | 0.7198 | 0.2474 | -0.4724 | 0.77 |
| GTE-base | Synthetic | natural_language | 1e-06 | 0.7198 | 0.4477 | -0.2721 | 0.78 |
| GTE-base | Synthetic | natural_language | 1e-05 | 0.7198 | 0.5675 | -0.1523 | 0.84 |
| GTE-base | Synthetic | natural_language | 0.0001 | 0.7198 | 0.5966 | -0.1232 | 0.84 |
| GTE-base | Synthetic | natural_language | 0.001 | 0.7198 | 0.6526 | -0.0672 | 0.87 |
| GTE-base | Synthetic | natural_language | 0.01 | 0.7198 | 0.6985 | -0.0213 | 0.91 |
| Nomic-embed-text | MTSamples | keyword | 1e-07 | 0.9203 | 0.8774 | -0.0429 | 0.94 |
| Nomic-embed-text | MTSamples | keyword | 1e-06 | 0.9203 | 0.8774 | -0.0429 | 0.94 |
| Nomic-embed-text | MTSamples | keyword | 1e-05 | 0.9203 | 0.8774 | -0.0429 | 0.94 |
| Nomic-embed-text | MTSamples | keyword | 0.0001 | 0.9203 | 0.8802 | -0.0401 | 0.95 |
| Nomic-embed-text | MTSamples | keyword | 0.001 | 0.9203 | 0.8938 | -0.0266 | 0.96 |
| Nomic-embed-text | MTSamples | keyword | 0.01 | 0.9203 | 0.902 | -0.0183 | 0.96 |
| Nomic-embed-text | MTSamples | natural_language | 1e-07 | 0.9367 | 0.9027 | -0.0340 | 0.95 |
| Nomic-embed-text | MTSamples | natural_language | 1e-06 | 0.9367 | 0.9027 | -0.0340 | 0.95 |
| Nomic-embed-text | MTSamples | natural_language | 1e-05 | 0.9367 | 0.9037 | -0.0330 | 0.96 |
| Nomic-embed-text | MTSamples | natural_language | 0.0001 | 0.9367 | 0.9131 | -0.0236 | 0.95 |
| Nomic-embed-text | MTSamples | natural_language | 0.001 | 0.9367 | 0.9303 | -0.0063 | 0.97 |
| Nomic-embed-text | MTSamples | natural_language | 0.01 | 0.9367 | 0.92875 | -0.0079 | 0.97 |
| Nomic-embed-text | PMC-Patients | keyword | 1e-07 | 0.9478 | 0.9222 | -0.0256 | 0.99 |
| Nomic-embed-text | PMC-Patients | keyword | 1e-06 | 0.9478 | 0.9206 | -0.0272 | 0.99 |
| Nomic-embed-text | PMC-Patients | keyword | 1e-05 | 0.9478 | 0.9206 | -0.0272 | 0.99 |
| Nomic-embed-text | PMC-Patients | keyword | 0.0001 | 0.9478 | 0.9221 | -0.0257 | 0.99 |
| Nomic-embed-text | PMC-Patients | keyword | 0.001 | 0.9478 | 0.9406 | -0.0072 | 1.0 |
| Nomic-embed-text | PMC-Patients | keyword | 0.01 | 0.9478 | 0.9525 | 0.0047 | 1.0 |
| Nomic-embed-text | PMC-Patients | natural_language | 1e-07 | 0.9708 | 0.927 | -0.0438 | 0.97 |
| Nomic-embed-text | PMC-Patients | natural_language | 1e-06 | 0.9708 | 0.927 | -0.0438 | 0.97 |
| Nomic-embed-text | PMC-Patients | natural_language | 1e-05 | 0.9708 | 0.927 | -0.0438 | 0.97 |
| Nomic-embed-text | PMC-Patients | natural_language | 0.0001 | 0.9708 | 0.92875 | -0.0421 | 0.98 |
| Nomic-embed-text | PMC-Patients | natural_language | 0.001 | 0.9708 | 0.9383 | -0.0325 | 0.99 |
| Nomic-embed-text | PMC-Patients | natural_language | 0.01 | 0.9708 | 0.9583 | -0.0125 | 0.99 |
| Nomic-embed-text | Synthetic | keyword | 1e-07 | 0.6999 | 0.5560 | -0.1440 | 0.76 |
| Nomic-embed-text | Synthetic | keyword | 1e-06 | 0.6999 | 0.5560 | -0.1440 | 0.76 |
| Nomic-embed-text | Synthetic | keyword | 1e-05 | 0.6999 | 0.5572 | -0.1427 | 0.77 |
| Nomic-embed-text | Synthetic | keyword | 0.0001 | 0.6999 | 0.5954 | -0.1045 | 0.83 |
| Nomic-embed-text | Synthetic | keyword | 0.001 | 0.6999 | 0.6774 | -0.0226 | 0.86 |
| Nomic-embed-text | Synthetic | keyword | 0.01 | 0.6999 | 0.6826 | -0.0173 | 0.92 |
| Nomic-embed-text | Synthetic | natural_language | 1e-07 | 0.6949 | 0.4716 | -0.2233 | 0.73 |
| Nomic-embed-text | Synthetic | natural_language | 1e-06 | 0.6949 | 0.4716 | -0.2233 | 0.73 |
| Nomic-embed-text | Synthetic | natural_language | 1e-05 | 0.6949 | 0.4768 | -0.2180 | 0.73 |
| Nomic-embed-text | Synthetic | natural_language | 0.0001 | 0.6949 | 0.5099 | -0.1850 | 0.75 |
| Nomic-embed-text | Synthetic | natural_language | 0.001 | 0.6949 | 0.6182 | -0.0767 | 0.82 |
| Nomic-embed-text | Synthetic | natural_language | 0.01 | 0.6949 | 0.6845 | -0.0103 | 0.88 |
| E5-Mistral-7B | MTSamples | keyword | 1e-07 | 0.1659 | 0.3363 | 0.1704 | 0.79 |
| E5-Mistral-7B | MTSamples | keyword | 1e-06 | 0.1659 | 0.5812 | 0.4153 | 0.79 |
| E5-Mistral-7B | MTSamples | keyword | 1e-05 | 0.1659 | 0.6255 | 0.4596 | 0.81 |
| E5-Mistral-7B | MTSamples | keyword | 0.0001 | 0.1659 | 0.6150 | 0.4492 | 0.81 |
| E5-Mistral-7B | MTSamples | keyword | 0.001 | 0.1659 | 0.5195 | 0.3536 | 0.82 |
| E5-Mistral-7B | MTSamples | keyword | 0.01 | 0.1659 | 0.2501 | 0.0843 | 0.58 |
| E5-Mistral-7B | MTSamples | natural_language | 1e-07 | 0.4126 | 0.4825 | 0.0699 | 0.88 |
| E5-Mistral-7B | MTSamples | natural_language | 1e-06 | 0.4126 | 0.6745 | 0.2619 | 0.88 |
| E5-Mistral-7B | MTSamples | natural_language | 1e-05 | 0.4126 | 0.6886 | 0.2760 | 0.89 |
| E5-Mistral-7B | MTSamples | natural_language | 0.0001 | 0.4126 | 0.6898 | 0.2772 | 0.89 |
| E5-Mistral-7B | MTSamples | natural_language | 0.001 | 0.4126 | 0.6801 | 0.2675 | 0.88 |
| E5-Mistral-7B | MTSamples | natural_language | 0.01 | 0.4126 | 0.5362 | 0.1236 | 0.78 |
| E5-Mistral-7B | PMC-Patients | keyword | 1e-07 | 0.5103 | 0.6605 | 0.1503 | 0.87 |
| E5-Mistral-7B | PMC-Patients | keyword | 1e-06 | 0.5103 | 0.6605 | 0.1503 | 0.87 |
| E5-Mistral-7B | PMC-Patients | keyword | 1e-05 | 0.5103 | 0.6622 | 0.1519 | 0.87 |
| E5-Mistral-7B | PMC-Patients | keyword | 0.0001 | 0.5103 | 0.6524 | 0.1421 | 0.87 |
| E5-Mistral-7B | PMC-Patients | keyword | 0.001 | 0.5103 | 0.6066 | 0.0963 | 0.87 |
| E5-Mistral-7B | PMC-Patients | keyword | 0.01 | 0.5103 | 0.4777 | -0.0325 | 0.76 |
| E5-Mistral-7B | PMC-Patients | natural_language | 1e-07 | 0.6205 | 0.8031 | 0.1827 | 0.93 |
| E5-Mistral-7B | PMC-Patients | natural_language | 1e-06 | 0.6205 | 0.8031 | 0.1827 | 0.93 |
| E5-Mistral-7B | PMC-Patients | natural_language | 1e-05 | 0.6205 | 0.8031 | 0.1827 | 0.93 |
| E5-Mistral-7B | PMC-Patients | natural_language | 0.0001 | 0.6205 | 0.7989 | 0.1785 | 0.93 |
| E5-Mistral-7B | PMC-Patients | natural_language | 0.001 | 0.6205 | 0.7730 | 0.1525 | 0.95 |
| E5-Mistral-7B | PMC-Patients | natural_language | 0.01 | 0.6205 | 0.6260 | 0.0055 | 0.89 |
| E5-Mistral-7B | Synthetic | keyword | 1e-07 | 0.1003 | 0.0561 | -0.0442 | 0.11 |
| E5-Mistral-7B | Synthetic | keyword | 1e-06 | 0.1003 | 0.0765 | -0.0238 | 0.24 |
| E5-Mistral-7B | Synthetic | keyword | 1e-05 | 0.1003 | 0.1645 | 0.0642 | 0.4 |
| E5-Mistral-7B | Synthetic | keyword | 0.0001 | 0.1003 | 0.1618 | 0.0615 | 0.39 |
| E5-Mistral-7B | Synthetic | keyword | 0.001 | 0.1003 | 0.1670 | 0.0667 | 0.43 |
| E5-Mistral-7B | Synthetic | keyword | 0.01 | 0.1003 | 0.1597 | 0.0594 | 0.43 |
| E5-Mistral-7B | Synthetic | natural_language | 1e-07 | 0.0977 | 0.0343 | -0.0634 | 0.17 |
| E5-Mistral-7B | Synthetic | natural_language | 1e-06 | 0.0977 | 0.0959 | -0.0018 | 0.36 |
| E5-Mistral-7B | Synthetic | natural_language | 1e-05 | 0.0977 | 0.1839 | 0.0862 | 0.43 |
| E5-Mistral-7B | Synthetic | natural_language | 0.0001 | 0.0977 | 0.1807 | 0.0830 | 0.45 |
| E5-Mistral-7B | Synthetic | natural_language | 0.001 | 0.0977 | 0.1744 | 0.0768 | 0.43 |
| E5-Mistral-7B | Synthetic | natural_language | 0.01 | 0.0977 | 0.1572 | 0.0595 | 0.36 |
| Phi-3-mini | MTSamples | keyword | 1e-07 | 0.5478 | 0.6437 | 0.0960 | 0.84 |
| Phi-3-mini | MTSamples | keyword | 1e-06 | 0.5478 | 0.6480 | 0.1002 | 0.85 |
| Phi-3-mini | MTSamples | keyword | 1e-05 | 0.5478 | 0.6476 | 0.0999 | 0.87 |
| Phi-3-mini | MTSamples | keyword | 0.0001 | 0.5478 | 0.6346 | 0.0869 | 0.92 |
| Phi-3-mini | MTSamples | keyword | 0.001 | 0.5478 | 0.6288 | 0.0811 | 0.92 |
| Phi-3-mini | MTSamples | keyword | 0.01 | 0.5478 | 0.5788 | 0.0310 | 0.89 |
| Phi-3-mini | MTSamples | natural_language | 1e-07 | 0.5409 | 0.6722 | 0.1313 | 0.82 |
| Phi-3-mini | MTSamples | natural_language | 1e-06 | 0.5409 | 0.6655 | 0.1246 | 0.82 |
| Phi-3-mini | MTSamples | natural_language | 1e-05 | 0.5409 | 0.6836 | 0.1427 | 0.84 |
| Phi-3-mini | MTSamples | natural_language | 0.0001 | 0.5409 | 0.6779 | 0.1369 | 0.86 |
| Phi-3-mini | MTSamples | natural_language | 0.001 | 0.5409 | 0.5942 | 0.0533 | 0.86 |
| Phi-3-mini | MTSamples | natural_language | 0.01 | 0.5409 | 0.5189 | -0.0220 | 0.8 |
| Phi-3-mini | PMC-Patients | keyword | 1e-07 | 0.5735 | 0.6973 | 0.1238 | 0.84 |
| Phi-3-mini | PMC-Patients | keyword | 1e-06 | 0.5735 | 0.7023 | 0.1288 | 0.84 |
| Phi-3-mini | PMC-Patients | keyword | 1e-05 | 0.5735 | 0.7008 | 0.1273 | 0.85 |
| Phi-3-mini | PMC-Patients | keyword | 0.0001 | 0.5735 | 0.6773 | 0.1038 | 0.88 |
| Phi-3-mini | PMC-Patients | keyword | 0.001 | 0.5735 | 0.6301 | 0.0566 | 0.9 |
| Phi-3-mini | PMC-Patients | keyword | 0.01 | 0.5735 | 0.5939 | 0.0204 | 0.87 |
| Phi-3-mini | PMC-Patients | natural_language | 1e-07 | 0.6572 | 0.6918 | 0.0346 | 0.95 |
| Phi-3-mini | PMC-Patients | natural_language | 1e-06 | 0.6572 | 0.6893 | 0.0321 | 0.95 |
| Phi-3-mini | PMC-Patients | natural_language | 1e-05 | 0.6572 | 0.6994 | 0.0423 | 0.94 |
| Phi-3-mini | PMC-Patients | natural_language | 0.0001 | 0.6572 | 0.6792 | 0.0220 | 0.94 |
| Phi-3-mini | PMC-Patients | natural_language | 0.001 | 0.6572 | 0.6701 | 0.0129 | 0.92 |
| Phi-3-mini | PMC-Patients | natural_language | 0.01 | 0.6572 | 0.6664 | 0.0092 | 0.92 |
| Phi-3-mini | Synthetic | keyword | 1e-07 | 0.4235 | 0.1069 | -0.3165 | 0.44 |
| Phi-3-mini | Synthetic | keyword | 1e-06 | 0.4235 | 0.2777 | -0.1458 | 0.59 |
| Phi-3-mini | Synthetic | keyword | 1e-05 | 0.4235 | 0.3066 | -0.1169 | 0.65 |
| Phi-3-mini | Synthetic | keyword | 0.0001 | 0.4235 | 0.3813 | -0.0422 | 0.7 |
| Phi-3-mini | Synthetic | keyword | 0.001 | 0.4235 | 0.4171 | -0.0064 | 0.8 |
| Phi-3-mini | Synthetic | keyword | 0.01 | 0.4235 | 0.4489 | 0.0254 | 0.79 |
| Phi-3-mini | Synthetic | natural_language | 1e-07 | 0.3969 | 0.1522 | -0.2448 | 0.44 |
| Phi-3-mini | Synthetic | natural_language | 1e-06 | 0.3969 | 0.3243 | -0.0727 | 0.6 |
| Phi-3-mini | Synthetic | natural_language | 1e-05 | 0.3969 | 0.3366 | -0.0604 | 0.63 |
| Phi-3-mini | Synthetic | natural_language | 0.0001 | 0.3969 | 0.4070 | 0.0101 | 0.73 |
| Phi-3-mini | Synthetic | natural_language | 0.001 | 0.3969 | 0.4707 | 0.0738 | 0.78 |
| Phi-3-mini | Synthetic | natural_language | 0.01 | 0.3969 | 0.4237 | 0.0267 | 0.69 |
| E5-Mistral-7B-ablation | MTSamples | keyword | 1e-07 | 0.7467 | 0.4584 | -0.2882 | 0.89 |
| E5-Mistral-7B-ablation | MTSamples | keyword | 1e-06 | 0.7467 | 0.7245 | -0.0222 | 0.91 |
| E5-Mistral-7B-ablation | MTSamples | keyword | 1e-05 | 0.7467 | 0.7220 | -0.0247 | 0.91 |
| E5-Mistral-7B-ablation | MTSamples | keyword | 0.0001 | 0.7467 | 0.7396 | -0.0071 | 0.91 |
| E5-Mistral-7B-ablation | MTSamples | keyword | 0.001 | 0.7467 | 0.7694 | 0.0227 | 0.94 |
| E5-Mistral-7B-ablation | MTSamples | keyword | 0.01 | 0.7467 | 0.7472 | 0.0005 | 0.94 |
| E5-Mistral-7B-ablation | MTSamples | natural_language | 1e-07 | 0.5511 | 0.6162 | 0.0651 | 0.86 |
| E5-Mistral-7B-ablation | MTSamples | natural_language | 1e-06 | 0.5511 | 0.7875 | 0.2364 | 0.87 |
| E5-Mistral-7B-ablation | MTSamples | natural_language | 1e-05 | 0.5511 | 0.7892 | 0.2381 | 0.87 |
| E5-Mistral-7B-ablation | MTSamples | natural_language | 0.0001 | 0.5511 | 0.7893 | 0.2382 | 0.88 |
| E5-Mistral-7B-ablation | MTSamples | natural_language | 0.001 | 0.5511 | 0.75275 | 0.2016 | 0.89 |
| E5-Mistral-7B-ablation | MTSamples | natural_language | 0.01 | 0.5511 | 0.5818 | 0.0307 | 0.92 |
| E5-Mistral-7B-ablation | PMC-Patients | keyword | 1e-07 | 0.7424 | 0.7687 | 0.0263 | 0.92 |
| E5-Mistral-7B-ablation | PMC-Patients | keyword | 1e-06 | 0.7424 | 0.7702 | 0.0278 | 0.92 |
| E5-Mistral-7B-ablation | PMC-Patients | keyword | 1e-05 | 0.7424 | 0.7681 | 0.0256 | 0.92 |
| E5-Mistral-7B-ablation | PMC-Patients | keyword | 0.0001 | 0.7424 | 0.7799 | 0.0375 | 0.92 |
| E5-Mistral-7B-ablation | PMC-Patients | keyword | 0.001 | 0.7424 | 0.7848 | 0.0423 | 0.92 |
| E5-Mistral-7B-ablation | PMC-Patients | keyword | 0.01 | 0.7424 | 0.74775 | 0.0053 | 0.93 |
| E5-Mistral-7B-ablation | PMC-Patients | natural_language | 1e-07 | 0.7586 | 0.90875 | 0.1501 | 0.96 |
| E5-Mistral-7B-ablation | PMC-Patients | natural_language | 1e-06 | 0.7586 | 0.90875 | 0.1501 | 0.96 |
| E5-Mistral-7B-ablation | PMC-Patients | natural_language | 1e-05 | 0.7586 | 0.90875 | 0.1501 | 0.96 |
| E5-Mistral-7B-ablation | PMC-Patients | natural_language | 0.0001 | 0.7586 | 0.9049 | 0.1463 | 0.97 |
| E5-Mistral-7B-ablation | PMC-Patients | natural_language | 0.001 | 0.7586 | 0.8849 | 0.1262 | 0.98 |
| E5-Mistral-7B-ablation | PMC-Patients | natural_language | 0.01 | 0.7586 | 0.8014 | 0.0428 | 0.97 |
| E5-Mistral-7B-ablation | Synthetic | keyword | 1e-07 | 0.6577 | 0.1473 | -0.5104 | 0.47 |
| E5-Mistral-7B-ablation | Synthetic | keyword | 1e-06 | 0.6577 | 0.3723 | -0.2854 | 0.68 |
| E5-Mistral-7B-ablation | Synthetic | keyword | 1e-05 | 0.6577 | 0.3740 | -0.2837 | 0.69 |
| E5-Mistral-7B-ablation | Synthetic | keyword | 0.0001 | 0.6577 | 0.4099 | -0.2478 | 0.74 |
| E5-Mistral-7B-ablation | Synthetic | keyword | 0.001 | 0.6577 | 0.5341 | -0.1236 | 0.86 |
| E5-Mistral-7B-ablation | Synthetic | keyword | 0.01 | 0.6577 | 0.6568 | -0.0008 | 0.94 |
| E5-Mistral-7B-ablation | Synthetic | natural_language | 1e-07 | 0.5231 | 0.1298 | -0.3934 | 0.44 |
| E5-Mistral-7B-ablation | Synthetic | natural_language | 1e-06 | 0.5231 | 0.3223 | -0.2008 | 0.56 |
| E5-Mistral-7B-ablation | Synthetic | natural_language | 1e-05 | 0.5231 | 0.3236 | -0.1995 | 0.58 |
| E5-Mistral-7B-ablation | Synthetic | natural_language | 0.0001 | 0.5231 | 0.3476 | -0.1755 | 0.67 |
| E5-Mistral-7B-ablation | Synthetic | natural_language | 0.001 | 0.5231 | 0.4636 | -0.0596 | 0.79 |
| E5-Mistral-7B-ablation | Synthetic | natural_language | 0.01 | 0.5231 | 0.5121 | -0.0110 | 0.82 |
| Nomic-embed-text-nopfx | MTSamples | keyword | 1e-07 | 0.9061 | 0.8801 | -0.0260 | 0.95 |
| Nomic-embed-text-nopfx | MTSamples | keyword | 1e-06 | 0.9061 | 0.8851 | -0.0210 | 0.95 |
| Nomic-embed-text-nopfx | MTSamples | keyword | 1e-05 | 0.9061 | 0.8851 | -0.0210 | 0.95 |
| Nomic-embed-text-nopfx | MTSamples | keyword | 0.0001 | 0.9061 | 0.8878 | -0.0183 | 0.95 |
| Nomic-embed-text-nopfx | MTSamples | keyword | 0.001 | 0.9061 | 0.8928 | -0.0133 | 0.95 |
| Nomic-embed-text-nopfx | MTSamples | keyword | 0.01 | 0.9061 | 0.9014 | -0.0047 | 0.97 |
| Nomic-embed-text-nopfx | MTSamples | natural_language | 1e-07 | 0.9331 | 0.9127 | -0.0204 | 0.96 |
| Nomic-embed-text-nopfx | MTSamples | natural_language | 1e-06 | 0.9331 | 0.9127 | -0.0204 | 0.96 |
| Nomic-embed-text-nopfx | MTSamples | natural_language | 1e-05 | 0.9331 | 0.9127 | -0.0204 | 0.96 |
| Nomic-embed-text-nopfx | MTSamples | natural_language | 0.0001 | 0.9331 | 0.915 | -0.0181 | 0.97 |
| Nomic-embed-text-nopfx | MTSamples | natural_language | 0.001 | 0.9331 | 0.9273 | -0.0058 | 0.98 |
| Nomic-embed-text-nopfx | MTSamples | natural_language | 0.01 | 0.9331 | 0.9316 | -0.0015 | 0.99 |
| Nomic-embed-text-nopfx | PMC-Patients | keyword | 1e-07 | 0.9587 | 0.907 | -0.0517 | 0.97 |
| Nomic-embed-text-nopfx | PMC-Patients | keyword | 1e-06 | 0.9587 | 0.907 | -0.0517 | 0.97 |
| Nomic-embed-text-nopfx | PMC-Patients | keyword | 1e-05 | 0.9587 | 0.907 | -0.0517 | 0.97 |
| Nomic-embed-text-nopfx | PMC-Patients | keyword | 0.0001 | 0.9587 | 0.9123 | -0.0463 | 0.97 |
| Nomic-embed-text-nopfx | PMC-Patients | keyword | 0.001 | 0.9587 | 0.9184 | -0.0402 | 0.98 |
| Nomic-embed-text-nopfx | PMC-Patients | keyword | 0.01 | 0.9587 | 0.9378 | -0.0209 | 1.0 |
| Nomic-embed-text-nopfx | PMC-Patients | natural_language | 1e-07 | 0.985 | 0.9625 | -0.0225 | 0.99 |
| Nomic-embed-text-nopfx | PMC-Patients | natural_language | 1e-06 | 0.985 | 0.9625 | -0.0225 | 0.99 |
| Nomic-embed-text-nopfx | PMC-Patients | natural_language | 1e-05 | 0.985 | 0.9635 | -0.0215 | 1.0 |
| Nomic-embed-text-nopfx | PMC-Patients | natural_language | 0.0001 | 0.985 | 0.96875 | -0.0162 | 1.0 |
| Nomic-embed-text-nopfx | PMC-Patients | natural_language | 0.001 | 0.985 | 0.9775 | -0.0075 | 1.0 |
| Nomic-embed-text-nopfx | PMC-Patients | natural_language | 0.01 | 0.985 | 0.9783 | -0.0067 | 1.0 |
| Nomic-embed-text-nopfx | Synthetic | keyword | 1e-07 | 0.7105 | 0.5309 | -0.1797 | 0.76 |
| Nomic-embed-text-nopfx | Synthetic | keyword | 1e-06 | 0.7105 | 0.5309 | -0.1797 | 0.76 |
| Nomic-embed-text-nopfx | Synthetic | keyword | 1e-05 | 0.7105 | 0.5352 | -0.1753 | 0.76 |
| Nomic-embed-text-nopfx | Synthetic | keyword | 0.0001 | 0.7105 | 0.5656 | -0.1449 | 0.8 |
| Nomic-embed-text-nopfx | Synthetic | keyword | 0.001 | 0.7105 | 0.6588 | -0.0517 | 0.88 |
| Nomic-embed-text-nopfx | Synthetic | keyword | 0.01 | 0.7105 | 0.7013 | -0.0092 | 0.92 |
| Nomic-embed-text-nopfx | Synthetic | natural_language | 1e-07 | 0.7079 | 0.5074 | -0.2005 | 0.72 |
| Nomic-embed-text-nopfx | Synthetic | natural_language | 1e-06 | 0.7079 | 0.5089 | -0.1991 | 0.72 |
| Nomic-embed-text-nopfx | Synthetic | natural_language | 1e-05 | 0.7079 | 0.5126 | -0.1954 | 0.74 |
| Nomic-embed-text-nopfx | Synthetic | natural_language | 0.0001 | 0.7079 | 0.5606 | -0.1474 | 0.78 |
| Nomic-embed-text-nopfx | Synthetic | natural_language | 0.001 | 0.7079 | 0.6348 | -0.0732 | 0.85 |
| Nomic-embed-text-nopfx | Synthetic | natural_language | 0.01 | 0.7079 | 0.6914 | -0.0166 | 0.92 |
| BERT-base-uncased | MTSamples | keyword | 1e-07 | 0.2381 | 0.3243 | 0.0862 | 0.56 |
| BERT-base-uncased | MTSamples | keyword | 1e-06 | 0.2381 | 0.3238 | 0.0857 | 0.56 |
| BERT-base-uncased | MTSamples | keyword | 1e-05 | 0.2381 | 0.3411 | 0.1030 | 0.57 |
| BERT-base-uncased | MTSamples | keyword | 0.0001 | 0.2381 | 0.3546 | 0.1166 | 0.56 |
| BERT-base-uncased | MTSamples | keyword | 0.001 | 0.2381 | 0.3141 | 0.0761 | 0.59 |
| BERT-base-uncased | MTSamples | keyword | 0.01 | 0.2381 | 0.1932 | -0.0448 | 0.47 |
| BERT-base-uncased | MTSamples | natural_language | 1e-07 | 0.2072 | 0.3687 | 0.1615 | 0.7 |
| BERT-base-uncased | MTSamples | natural_language | 1e-06 | 0.2072 | 0.3704 | 0.1631 | 0.71 |
| BERT-base-uncased | MTSamples | natural_language | 1e-05 | 0.2072 | 0.3782 | 0.1709 | 0.71 |
| BERT-base-uncased | MTSamples | natural_language | 0.0001 | 0.2072 | 0.3755 | 0.1683 | 0.7 |
| BERT-base-uncased | MTSamples | natural_language | 0.001 | 0.2072 | 0.2817 | 0.0744 | 0.69 |
| BERT-base-uncased | MTSamples | natural_language | 0.01 | 0.2072 | 0.1814 | -0.0258 | 0.58 |
| BERT-base-uncased | PMC-Patients | keyword | 1e-07 | 0.2657 | 0.2440 | -0.0217 | 0.52 |
| BERT-base-uncased | PMC-Patients | keyword | 1e-06 | 0.2657 | 0.2446 | -0.0211 | 0.52 |
| BERT-base-uncased | PMC-Patients | keyword | 1e-05 | 0.2657 | 0.2488 | -0.0169 | 0.5 |
| BERT-base-uncased | PMC-Patients | keyword | 0.0001 | 0.2657 | 0.2840 | 0.0183 | 0.54 |
| BERT-base-uncased | PMC-Patients | keyword | 0.001 | 0.2657 | 0.2811 | 0.0154 | 0.5 |
| BERT-base-uncased | PMC-Patients | keyword | 0.01 | 0.2657 | 0.2505 | -0.0152 | 0.49 |
| BERT-base-uncased | PMC-Patients | natural_language | 1e-07 | 0.2635 | 0.4006 | 0.1372 | 0.63 |
| BERT-base-uncased | PMC-Patients | natural_language | 1e-06 | 0.2635 | 0.4015 | 0.138 | 0.63 |
| BERT-base-uncased | PMC-Patients | natural_language | 1e-05 | 0.2635 | 0.4215 | 0.1580 | 0.64 |
| BERT-base-uncased | PMC-Patients | natural_language | 0.0001 | 0.2635 | 0.3998 | 0.1364 | 0.64 |
| BERT-base-uncased | PMC-Patients | natural_language | 0.001 | 0.2635 | 0.3342 | 0.0708 | 0.63 |
| BERT-base-uncased | PMC-Patients | natural_language | 0.01 | 0.2635 | 0.2429 | -0.0206 | 0.58 |
| BERT-base-uncased | Synthetic | keyword | 1e-07 | 0.2991 | 0.1549 | -0.1441 | 0.35 |
| BERT-base-uncased | Synthetic | keyword | 1e-06 | 0.2991 | 0.1564 | -0.1427 | 0.36 |
| BERT-base-uncased | Synthetic | keyword | 1e-05 | 0.2991 | 0.1730 | -0.1261 | 0.36 |
| BERT-base-uncased | Synthetic | keyword | 0.0001 | 0.2991 | 0.2339 | -0.0651 | 0.4 |
| BERT-base-uncased | Synthetic | keyword | 0.001 | 0.2991 | 0.2679 | -0.0312 | 0.44 |
| BERT-base-uncased | Synthetic | keyword | 0.01 | 0.2991 | 0.2855 | -0.0136 | 0.47 |
| BERT-base-uncased | Synthetic | natural_language | 1e-07 | 0.2242 | 0.1461 | -0.0780 | 0.35 |
| BERT-base-uncased | Synthetic | natural_language | 1e-06 | 0.2242 | 0.1464 | -0.0778 | 0.35 |
| BERT-base-uncased | Synthetic | natural_language | 1e-05 | 0.2242 | 0.1563 | -0.0678 | 0.36 |
| BERT-base-uncased | Synthetic | natural_language | 0.0001 | 0.2242 | 0.2063 | -0.0179 | 0.4 |
| BERT-base-uncased | Synthetic | natural_language | 0.001 | 0.2242 | 0.2535 | 0.0293 | 0.44 |
| BERT-base-uncased | Synthetic | natural_language | 0.01 | 0.2242 | 0.2240 | -0.0002 | 0.46 |
| BioMistral-7B | MTSamples | keyword | 1e-07 | 0.3433 | 0.4043 | 0.0609 | 0.77 |
| BioMistral-7B | MTSamples | keyword | 1e-06 | 0.3433 | 0.5743 | 0.2310 | 0.82 |
| BioMistral-7B | MTSamples | keyword | 1e-05 | 0.3433 | 0.5778 | 0.2345 | 0.82 |
| BioMistral-7B | MTSamples | keyword | 0.0001 | 0.3433 | 0.5720 | 0.2287 | 0.84 |
| BioMistral-7B | MTSamples | keyword | 0.001 | 0.3433 | 0.5191 | 0.1758 | 0.8 |
| BioMistral-7B | MTSamples | keyword | 0.01 | 0.3433 | 0.4072 | 0.0639 | 0.76 |
| BioMistral-7B | MTSamples | natural_language | 1e-07 | 0.4813 | 0.4535 | -0.0277 | 0.8 |
| BioMistral-7B | MTSamples | natural_language | 1e-06 | 0.4813 | 0.5635 | 0.0822 | 0.8 |
| BioMistral-7B | MTSamples | natural_language | 1e-05 | 0.4813 | 0.5636 | 0.0824 | 0.8 |
| BioMistral-7B | MTSamples | natural_language | 0.0001 | 0.4813 | 0.5813 | 0.1000 | 0.8 |
| BioMistral-7B | MTSamples | natural_language | 0.001 | 0.4813 | 0.6036 | 0.1224 | 0.83 |
| BioMistral-7B | MTSamples | natural_language | 0.01 | 0.4813 | 0.4891 | 0.0079 | 0.82 |
| BioMistral-7B | PMC-Patients | keyword | 1e-07 | 0.4191 | 0.5829 | 0.1638 | 0.78 |
| BioMistral-7B | PMC-Patients | keyword | 1e-06 | 0.4191 | 0.5825 | 0.1634 | 0.78 |
| BioMistral-7B | PMC-Patients | keyword | 1e-05 | 0.4191 | 0.5799 | 0.1608 | 0.78 |
| BioMistral-7B | PMC-Patients | keyword | 0.0001 | 0.4191 | 0.5841 | 0.1650 | 0.78 |
| BioMistral-7B | PMC-Patients | keyword | 0.001 | 0.4191 | 0.5385 | 0.1193 | 0.82 |
| BioMistral-7B | PMC-Patients | keyword | 0.01 | 0.4191 | 0.4258 | 0.0066 | 0.78 |
| BioMistral-7B | PMC-Patients | natural_language | 1e-07 | 0.5499 | 0.6962 | 0.1462 | 0.88 |
| BioMistral-7B | PMC-Patients | natural_language | 1e-06 | 0.5499 | 0.6962 | 0.1462 | 0.88 |
| BioMistral-7B | PMC-Patients | natural_language | 1e-05 | 0.5499 | 0.6972 | 0.1472 | 0.89 |
| BioMistral-7B | PMC-Patients | natural_language | 0.0001 | 0.5499 | 0.6967 | 0.1468 | 0.91 |
| BioMistral-7B | PMC-Patients | natural_language | 0.001 | 0.5499 | 0.6450 | 0.0950 | 0.89 |
| BioMistral-7B | PMC-Patients | natural_language | 0.01 | 0.5499 | 0.5648 | 0.0149 | 0.88 |
| BioMistral-7B | Synthetic | keyword | 1e-07 | 0.3694 | 0.0944 | -0.2750 | 0.35 |
| BioMistral-7B | Synthetic | keyword | 1e-06 | 0.3694 | 0.1950 | -0.1744 | 0.44 |
| BioMistral-7B | Synthetic | keyword | 1e-05 | 0.3694 | 0.2077 | -0.1618 | 0.46 |
| BioMistral-7B | Synthetic | keyword | 0.0001 | 0.3694 | 0.2154 | -0.1540 | 0.47 |
| BioMistral-7B | Synthetic | keyword | 0.001 | 0.3694 | 0.2624 | -0.1071 | 0.56 |
| BioMistral-7B | Synthetic | keyword | 0.01 | 0.3694 | 0.3826 | 0.0132 | 0.66 |
| BioMistral-7B | Synthetic | natural_language | 1e-07 | 0.3784 | 0.0780 | -0.3004 | 0.33 |
| BioMistral-7B | Synthetic | natural_language | 1e-06 | 0.3784 | 0.2616 | -0.1168 | 0.41 |
| BioMistral-7B | Synthetic | natural_language | 1e-05 | 0.3784 | 0.2689 | -0.1095 | 0.43 |
| BioMistral-7B | Synthetic | natural_language | 0.0001 | 0.3784 | 0.3053 | -0.0731 | 0.49 |
| BioMistral-7B | Synthetic | natural_language | 0.001 | 0.3784 | 0.3613 | -0.0171 | 0.55 |
| BioMistral-7B | Synthetic | natural_language | 0.01 | 0.3784 | 0.3661 | -0.0123 | 0.6 |
