# Supplementary material for: Deriving a Deployment Threshold for Benefit and Harm in Clinical AI: Multi-Institutional Decision-Analytic Modeling Study
Source: JMIR AI. 2026 Oct 23;5:e109863. doi: 10.2196/109863 (PMC13743961; doi:10.2196/109863)
Supplement: Supplementary file 7 [file ai_v5i1e109863_app7.docx]

**Economic and classification sensitivity analyses**

*One-way and probabilistic sensitivity on the parameters that set the absolute monetary scale; the time-horizon, discount-rate and exchange-rate analyses; the published European adverse-event cost scenarios; and the classification-boundary sweep. Every table is reproduced by paper9_sensitivity.py and paper9_boundary_sweep.py from the archived code.*

**Table S1. One-way sensitivity on the illustrative parameters**

*Each parameter varied across the 2.5th to 97.5th percentile of its stated distribution, others held at base case.*

| **parameter** | **2.5th percentile** | **base value** | **97.5th percentile** | **M at low (EUR)** | **M at high (EUR)** | **p* at low** | **p* at high** |
| --- | --- | --- | --- | --- | --- | --- | --- |
| alpha | 0.4060 | 0.7000 | 0.9940 | 199,548,576 | 488,536,246 | 0.502830 | 0.502801 |
| adoption | 0.3514 | 0.6000 | 0.8234 | 201,482,202 | 472,133,185 | 0.502830 | 0.502802 |
| p_event | 0.0463 | 0.1200 | 0.2223 | 132,711,210 | 637,269,967 | 0.502854 | 0.502797 |

**Table S2. Probabilistic sensitivity analysis, 50,000 draws**

*Illustrative parameters, adverse-event cost, utility loss and both strategy-cost terms drawn jointly. The adverse-event cost is Gamma(2.5, C_event/2.5), the scale derived from the declared mean.*

| **quantity** | **mean** | **2.5th percentile** | **median** | **97.5th percentile** |
| --- | --- | --- | --- | --- |
| M (EUR) | 344,301,016 | 38,267,786 | 261,867,680 | 1,131,790,498 |
| K (EUR) | 1,469 | 356 | 1,331 | 3,359 |
| K/M | 9.13e-06 | 7.16e-07 | 5.03e-06 | 4.29e-05 |
| p* | 0.502842 | 0.502786 | 0.502815 | 0.503063 |

**Table S3. Time horizon and discount rate**

*The discount factor enters the value exposed and the deployment cost together and largely cancels.*

| **discount rate** | **horizon (years)** | **discount factor** | **M (EUR)** | **T (EUR)** | **p*** |
| --- | --- | --- | --- | --- | --- |
| 0% | 1 | 1.0000 | 77,281,037 | 950 | 0.502862 |
| 0% | 2 | 2.0000 | 154,562,074 | 1,100 | 0.502828 |
| 0% | 3 | 3.0000 | 231,843,111 | 1,250 | 0.502817 |
| 0% | 5 | 5.0000 | 386,405,185 | 1,550 | 0.502808 |
| 0% | 10 | 10.0000 | 772,810,369 | 2,300 | 0.502801 |
| 3% | 1 | 0.9709 | 75,030,133 | 946 | 0.502865 |
| 3% | 2 | 1.9135 | 147,874,922 | 1,087 | 0.502830 |
| 3% | 3 | 2.8286 | 218,598,019 | 1,224 | 0.502819 |
| 3% | 5 | 4.5797 | 353,924,520 | 1,487 | 0.502809 |
| 3% | 10 | 8.5302 | 659,222,920 | 2,080 | 0.502802 |
| 4% | 1 | 0.9615 | 74,308,689 | 944 | 0.502865 |
| 4% | 2 | 1.8861 | 145,759,352 | 1,083 | 0.502831 |
| 4% | 3 | 2.7751 | 214,461,913 | 1,216 | 0.502819 |
| 4% | 5 | 4.4518 | 344,041,446 | 1,468 | 0.502810 |
| 4% | 10 | 8.1109 | 626,818,436 | 2,017 | 0.502803 |

**Table S4. Exchange rate**

*Completed Norges Bank annual averages. An incomplete year has no annual average and is not reported as one.*

| **basis** | **rate (NOK/EUR)** | **C_event (EUR)** | **lambda (EUR/QALY)** | **p*** |
| --- | --- | --- | --- | --- |
| NB annual average 2024 | 11.6276 | 11,493.5 | 23,650.6 | 0.502810 |
| NB annual average 2025 | 11.7188 | 11,404.1 | 23,466.6 | 0.502810 |

**Table S5. Adverse-event treatment cost against the published European estimates**

*Base variant shown; alternative utility loss and severity-weighted willingness to pay are in the archived output.*

| **cost source** | **bound** | **C_event (EUR)** | **M (EUR)** | **p*** |
| --- | --- | --- | --- | --- |
| Norwegian (SAMDATA 2024, NB 2024 rate) | central | 11,494 | 344,041,446 | 0.502810 |
| Durand 2024 (review, per hospitalisation) | low | 6,000 | 180,107,386 | 0.502835 |
| Durand 2024 (review, per hospitalisation) | central | 8,000 | 239,790,297 | 0.502822 |
| Durand 2024 (review, per hospitalisation) | high | 10,000 | 299,473,208 | 0.502814 |
| Laroche 2025 (IATROSTAT-ECO, 2023 tariffs) | low | 618 | 19,500,673 | 0.503276 |
| Laroche 2025 (IATROSTAT-ECO, 2023 tariffs) | central | 5,974 | 179,331,508 | 0.502835 |
| Laroche 2025 (IATROSTAT-ECO, 2023 tariffs) | high | 27,380 | 818,117,704 | 0.502794 |
| Laroche 2025 (IATROSTAT-ECO, 2018 tariffs) | low | 514 | 16,397,162 | 0.503369 |
| Laroche 2025 (IATROSTAT-ECO, 2018 tariffs) | central | 5,208 | 156,472,954 | 0.502843 |
| Laroche 2025 (IATROSTAT-ECO, 2018 tariffs) | high | 23,355 | 698,005,846 | 0.502796 |

**Table S6. Classification-boundary sweep**

*A configuration is assigned to the harmed group where its mean effect is below tau; tau = 0 is the rule used. Rows at 0.01 intervals, with the intermediate state at tau = +0.0375 so all four thresholds reported in Results appear.*

| **boundary tau** | **benefited** | **harmed** | **d_ben** | **d_harm** | **sp at 6 conditions** | **requirement at 6** | **p*** |
| --- | --- | --- | --- | --- | --- | --- | --- |
| -0.0500 | 7 | 6 | +0.0627 | -0.0867 | 0.9962 | 0.0066 | 0.5805 |
| -0.0400 | 7 | 6 | +0.0627 | -0.0867 | 0.9962 | 0.0066 | 0.5805 |
| -0.0300 | 7 | 6 | +0.0627 | -0.0867 | 0.9962 | 0.0066 | 0.5805 |
| -0.0200 | 7 | 6 | +0.0627 | -0.0867 | 0.9962 | 0.0066 | 0.5805 |
| -0.0100 | 6 | 7 | +0.0757 | -0.0766 | 0.9362 | 0.0712 | 0.5028 |
| +0.0000 | 6 | 7 | +0.0757 | -0.0766 | 0.9362 | 0.0712 | 0.5028 |
| +0.0100 | 6 | 7 | +0.0757 | -0.0766 | 0.9362 | 0.0712 | 0.5028 |
| +0.0200 | 6 | 7 | +0.0757 | -0.0766 | 0.9362 | 0.0712 | 0.5028 |
| +0.0300 | 6 | 7 | +0.0757 | -0.0766 | 0.9362 | 0.0712 | 0.5028 |
| +0.0375 | 5 | 8 | +0.0835 | -0.0624 | 0.9149 | 0.0690 | 0.4277 |
| +0.0400 | 4 | 9 | +0.0946 | -0.0511 | 0.9040 | 0.0564 | 0.3508 |
| +0.0500 | 4 | 9 | +0.0946 | -0.0511 | 0.9040 | 0.0564 | 0.3508 |
