# Supplementary material for: Deriving a Deployment Threshold for Benefit and Harm in Clinical AI: Multi-Institutional Decision-Analytic Modeling Study
Source: JMIR AI. 2026 Oct 23;5:e109863. doi: 10.2196/109863 (PMC13743961; doi:10.2196/109863)
Supplement: Supplementary file 8 [file ai_v5i1e109863_app8.docx]

**System extraction frame and coding definitions**

*All 55 clinical retrieval-augmented generation systems, deduplicated from two systematic reviews. Tables S1 to S4 present the same 55 systems and summary counts from four complementary angles: what each reports, what could be inferred and on what evidence, how each is classified under the four prespecified classification assumptions, and the distribution of interface classes across the frame.*

**Scope of these codings**

Every coding in this frame is MECHANISTIC: it records an implementation characteristic expected to correspond to the response-defined subgroup of the decision model, not a measured response to the intervention. No system in the frame was evaluated under the intervention. The counts therefore support proxy-based partial identification under stated classification assumptions; they do not bound response-defined prevalence, which is unidentified without such an assumption.

**Mechanistic affected criterion**

A system is coded as meeting the hypothesised affected mechanism only where both of the following hold.

• The representation actually used for retrieval can be established from the primary article.

• That representation was not shown to have been calibrated by the model's training objective.

Systems whose extraction step cannot be established from the primary article are coded UNKNOWN, not unaffected. One of 55 systems meets this mechanistic criterion. Because no system in the frame was evaluated under the intervention, that count does not bound response-defined prevalence; it enters the analysis only under explicit classification assumptions.

**Reported pooling**

None of the 55 systems reports a retrieval pooling strategy as such. One system (A50) reports enough implementation detail elsewhere in its methods to infer the extraction step; the inference, its basis, and the primary-source location are recorded in their own columns.

**Coding stability**

A50 is Tier 2 in the core, expanded and adversarial frames. The source workbook additionally flagged it ambiguous under the original rationale, namely that no similarity-tuned checkpoint was identified. The manuscript establishes that this rationale is invalid, because citing a base-model paper does not establish the absence of a similarity-tuned checkpoint. Under the extracted-representation basis the coding is not ambiguous. Both the superseded rationale and the current basis are recorded so that the change is auditable.

**Interface class**

| **interface class** | **meaning** |
| --- | --- |
| commercial interface | pooling is vendor-fixed and the geometry is undisclosed |
| self-hosted checkpoint | the checkpoint is identified and inspectable |
| checkpoint unspecified | no identifiable checkpoint is named |
| not a pooled dense retriever | corpus-only whitening is undefined for the system |

**Table S1. Reported implementation characteristics**

| **Canonical ID** | **System** | **Source frame** | **Reported operational encoder/retriever** | **Interface class** | **Reported pooling** |
| --- | --- | --- | --- | --- | --- |
| A01 | Abdullahi / CliniqIR | Amugongo | ClinicalBERT, PubMedBERT, SciBERT, SapBERT, CODER, MedCPT; selected MedCPT + BM25 | self-hosted checkpoint | not reported as such |
| A04 | Aratesh | Amugongo | OpenAI text-embedding-ada-002 | commercial interface | not reported as such |
| A05 | Azimi / RAP | Amugongo | Amazon Titan Text Embeddings V2 | commercial interface | not reported as such |
| A06 | Benfenati | Amugongo | GTE | self-hosted checkpoint | not reported as such |
| A07 | Bora & Cuayáhuitl | Amugongo | gtr-t5-large | self-hosted checkpoint | not reported as such |
| A09 | Chen [22] | Amugongo | all-MiniLM-L6-v2 | self-hosted checkpoint | not reported as such |
| A12 | Fukushima / JGCLLM | Amugongo | GLuCoSE-base-ja | self-hosted checkpoint | not reported as such |
| A14 | Garcia | Amugongo | BGE | self-hosted checkpoint | not reported as such |
| A15 | Ge | Amugongo | OpenAI text-embedding-ada-002 | commercial interface | not reported as such |
| A17 | Guo / RALL | Amugongo | Dense Passage Retriever with fine-tuned query encoder | self-hosted checkpoint | not reported as such |
| A18 | Jeong / Self-BioRAG | Amugongo | MedCPT | self-hosted checkpoint | not reported as such |
| A19 | Jia | Amugongo | BGE | self-hosted checkpoint | not reported as such |
| A20 | Jiang | Amugongo | BGE | self-hosted checkpoint | not reported as such |
| A21 | Jin [69] | Amugongo | OpenAI text-embedding-ada-002 | commercial interface | not reported as such |
| A23 | Hou | Amugongo | OpenAI text-embedding-3-small | commercial interface | not reported as such |
| A25 | Ke [73] | Amugongo | OpenAI text-embedding-ada-002 | commercial interface | not reported as such |
| A27 | Klang | Amugongo | GIST-large-embedding-v0 | self-hosted checkpoint | not reported as such |
| A29 | Lee | Amugongo | OpenAI embedding models | commercial interface | not reported as such |
| A31 | Li [80] | Amugongo | OpenAI text-embedding-3-small | commercial interface | not reported as such |
| A32 | Long / ChatENT | Amugongo | OpenAI text-embedding-ada-002 | commercial interface | not reported as such |
| A35 | Luo | Amugongo | SBERT | self-hosted checkpoint | not reported as such |
| A39 | Murugan | Amugongo | OpenAI text-embedding-ada-002 | commercial interface | not reported as such |
| A40 | Neupane | Amugongo | OpenAI text-embedding-ada-002 | commercial interface | not reported as such |
| A41 | Ong [88] | Amugongo | OpenAI text-embedding-ada-002 + bge-small-en-v1.5 | commercial interface | not reported as such |
| A42 | Painter | Amugongo | OpenAI text-embedding-ada-002 | commercial interface | not reported as such |
| A43 | Pang | Amugongo | Fine-tuned embedding model | checkpoint unspecified | not reported as such |
| A44 | Parmanto | Amugongo | BGE | self-hosted checkpoint | not reported as such |
| A45 | Quidwai | Amugongo | bge-small-en-v1.5 | self-hosted checkpoint | not reported as such |
| A46 | Ranjit | Amugongo | ALBEF | not a pooled dense retriever | not reported as such |
| A47 | Rau | Amugongo | OpenAI text-embedding-ada-002 | commercial interface | not reported as such |
| A48 | Russe | Amugongo | OpenAI text-embedding-ada-002 | commercial interface | not reported as such |
| A50 | Shi / Chat-Orthopedist | Amugongo | MPNet; primary article cites the original MPNet base-model paper | self-hosted checkpoint | not reported as such |
| A51 | Soman / KG-RAG | Amugongo | PubMedBERT selected for context embeddings | self-hosted checkpoint | not reported as such |
| A52 | So | Amugongo | OpenAI text-embedding-ada-002 | commercial interface | not reported as such |
| A53 | Soong | Amugongo | OpenAI text-embedding-ada-002 | commercial interface | not reported as such |
| A54 | Steybe / GuideGPT | Amugongo | OpenAI text-embedding-ada-002 | commercial interface | not reported as such |
| A56 | Unlu / RECTIFIER | Amugongo | OpenAI text-embedding-ada-002 | commercial interface | not reported as such |
| A59 | Wang [106] | Amugongo | ColBERT | not a pooled dense retriever | not reported as such |
| A60 | Wang [107] / RISE | Amugongo | OpenAI text-embedding-ada-002 | commercial interface | not reported as such |
| A61 | Wornow | Amugongo | MiniLM + BGE | self-hosted checkpoint | not reported as such |
| A63 | Wu | Amugongo | OpenAI text-embedding-3-large | commercial interface | not reported as such |
| A64 | Yu, Guo & Sano | Amugongo | OpenAI text-embedding-ada-002 | commercial interface | not reported as such |
| A65 | Yu | Amugongo | MiniLM sentence embedding | self-hosted checkpoint | not reported as such |
| A66 | Zakka | Amugongo | OpenAI text-embedding-ada-002 | commercial interface | not reported as such |
| A67 | Ziletti | Amugongo | bge-small-en-v1.5 | self-hosted checkpoint | not reported as such |
| A69 | Zheng / RAGPR | Amugongo | FastText + SBERT + OpenAI text-embedding-3-large | commercial interface | not reported as such |
| A70 | Zhuo | Amugongo | gte-base-zh with embedding fine-tuning | self-hosted checkpoint | not reported as such |
| H01 | AskFDALabel | He | Sentence Transformer | checkpoint unspecified | not reported as such |
| H02 | CaLM | He | BGE embedding | self-hosted checkpoint | not reported as such |
| H06 | RAG-GPT | He | BGE embedding | self-hosted checkpoint | not reported as such |
| H11 | DRAGON-AI | He | OpenAI text embedding | commercial interface | not reported as such |
| H15 | Endo-chat | He | OpenAI text embedding | commercial interface | not reported as such |
| H20 | InfectA-Chat | He | OpenAI text embedding | commercial interface | not reported as such |
| H27 | BiomedRAG | He | Tailored learned chunk scorer supervised by LLM | not a pooled dense retriever | not reported as such |
| H33 | RAMIE | He | MedCPT + Contriever + BMRetriever | self-hosted checkpoint | not reported as such |

**Table S2. Inferred extraction and the evidence for it**

| **Canonical ID** | **Inferred extraction/representation** | **Basis for inference** | **Primary-source evidence** | **Extraction step established** | **Coding stability** | **Superseded coding rationale** |
| --- | --- | --- | --- | --- | --- | --- |
| A01 |  |  |  |  | stable across frames |  |
| A04 |  |  |  |  | stable across frames |  |
| A05 |  |  |  |  | stable across frames |  |
| A06 |  |  |  |  | stable across frames |  |
| A07 |  |  |  |  | stable across frames |  |
| A09 |  |  |  |  | stable across frames |  |
| A12 |  |  |  |  | stable across frames |  |
| A14 |  |  |  |  | stable across frames |  |
| A15 |  |  |  |  | stable across frames |  |
| A17 |  |  |  |  | stable across frames |  |
| A18 |  |  |  |  | stable across frames |  |
| A19 |  |  |  |  | stable across frames |  |
| A20 |  |  |  |  | stable across frames |  |
| A21 |  |  |  |  | stable across frames |  |
| A23 |  |  |  |  | stable across frames |  |
| A25 |  |  |  |  | stable across frames |  |
| A27 |  |  |  |  | stable across frames |  |
| A29 |  |  |  |  | stable across frames |  |
| A31 |  |  |  |  | stable across frames |  |
| A32 |  |  |  |  | stable across frames |  |
| A35 |  |  |  |  | stable across frames |  |
| A39 |  |  |  |  | stable across frames |  |
| A40 |  |  |  |  | stable across frames |  |
| A41 |  |  |  |  | stable across frames |  |
| A42 |  |  |  |  | stable across frames |  |
| A43 |  |  |  |  | stable across frames |  |
| A44 |  |  |  |  | stable across frames |  |
| A45 |  |  |  |  | stable across frames |  |
| A46 |  |  |  |  | stable across frames |  |
| A47 |  |  |  |  | stable across frames |  |
| A48 |  |  |  |  | stable across frames |  |
| A50 | classification-token ([CLS]) representation | The primary article reports extraction of the [CLS] representation and identifies MPNet by reference to the original MPNet model (Song et al.) rather than a retrieval-trained sentence-embedding checkpoint. On the reported implementation the retrieval representation was therefore not shown to have been calibrated by a retrieval-specific objective. Membership follows from the extracted representation, not from the training label. | Section 3.2, doi:10.1145/3584371.3612956 | yes - see Basis for inference | Tier 2 in core, expanded and adversarial frames; flagged ambiguous in the workbook under the superseded rationale, stable under the extracted-representation basis | workbook audit note records the original rationale (no similarity-tuned checkpoint identified), superseded; see Basis for inference |
| A51 |  |  |  | no - model family named, extraction step not reported | stable across frames |  |
| A52 |  |  |  |  | stable across frames |  |
| A53 |  |  |  |  | stable across frames |  |
| A54 |  |  |  |  | stable across frames |  |
| A56 |  |  |  |  | stable across frames |  |
| A59 |  |  |  |  | stable across frames |  |
| A60 |  |  |  |  | stable across frames |  |
| A61 |  |  |  |  | stable across frames |  |
| A63 |  |  |  |  | stable across frames |  |
| A64 |  |  |  |  | stable across frames |  |
| A65 |  |  |  |  | stable across frames |  |
| A66 |  |  |  |  | stable across frames |  |
| A67 |  |  |  |  | stable across frames |  |
| A69 |  |  |  |  | stable across frames |  |
| A70 |  |  |  |  | stable across frames |  |
| H01 |  |  |  |  | stable across frames |  |
| H02 |  |  |  |  | stable across frames |  |
| H06 |  |  |  |  | stable across frames |  |
| H11 |  |  |  |  | stable across frames |  |
| H15 |  |  |  |  | stable across frames |  |
| H20 |  |  |  |  | stable across frames |  |
| H27 |  |  |  |  | stable across frames |  |
| H33 |  |  |  |  | stable across frames |  |

**Table S3. Classification under each prespecified assumption**

| **Canonical ID** | **Confirmed affected** | **Confidence** | **Rung 1: no assumptions** | **Rung 2: contrastive training implies non-membership** | **Rung 3: vendor claims accepted** | **Rung 4: non-dense excluded** |
| --- | --- | --- | --- | --- | --- | --- |
| A01 | no | High | unknown | coded unaffected | coded unaffected | coded unaffected |
| A04 | no | Moderate | unknown | unknown | coded unaffected | coded unaffected |
| A05 | no | Moderate | unknown | unknown | coded unaffected | coded unaffected |
| A06 | no | Moderate | unknown | coded unaffected | coded unaffected | coded unaffected |
| A07 | no | Moderate | unknown | coded unaffected | coded unaffected | coded unaffected |
| A09 | no | Moderate | unknown | coded unaffected | coded unaffected | coded unaffected |
| A12 | no | Moderate | unknown | coded unaffected | coded unaffected | coded unaffected |
| A14 | no | Moderate | unknown | coded unaffected | coded unaffected | coded unaffected |
| A15 | no | Moderate | unknown | unknown | coded unaffected | coded unaffected |
| A17 | no | High | unknown | coded unaffected | coded unaffected | coded unaffected |
| A18 | no | High | unknown | coded unaffected | coded unaffected | coded unaffected |
| A19 | no | Moderate | unknown | coded unaffected | coded unaffected | coded unaffected |
| A20 | no | Moderate | unknown | coded unaffected | coded unaffected | coded unaffected |
| A21 | no | Moderate | unknown | unknown | coded unaffected | coded unaffected |
| A23 | no | Moderate | unknown | unknown | coded unaffected | coded unaffected |
| A25 | no | Moderate | unknown | unknown | coded unaffected | coded unaffected |
| A27 | no | Moderate | unknown | coded unaffected | coded unaffected | coded unaffected |
| A29 | no | Moderate | unknown | unknown | coded unaffected | coded unaffected |
| A31 | no | Moderate | unknown | unknown | coded unaffected | coded unaffected |
| A32 | no | Moderate | unknown | unknown | coded unaffected | coded unaffected |
| A35 | no | Moderate | unknown | coded unaffected | coded unaffected | coded unaffected |
| A39 | no | Moderate | unknown | unknown | coded unaffected | coded unaffected |
| A40 | no | Moderate | unknown | unknown | coded unaffected | coded unaffected |
| A41 | no | Moderate | unknown | unknown | coded unaffected | coded unaffected |
| A42 | no | Moderate | unknown | unknown | coded unaffected | coded unaffected |
| A43 | no | Moderate | unknown | unknown | unknown | unknown |
| A44 | no | Moderate | unknown | coded unaffected | coded unaffected | coded unaffected |
| A45 | no | Moderate | unknown | coded unaffected | coded unaffected | coded unaffected |
| A46 | no | Moderate | unknown | coded unaffected | coded unaffected | excluded |
| A47 | no | Moderate | unknown | unknown | coded unaffected | coded unaffected |
| A48 | no | Moderate | unknown | unknown | coded unaffected | coded unaffected |
| A50 | yes | High | affected | affected | affected | affected |
| A51 | no | High | unknown | unknown | unknown | unknown |
| A52 | no | Moderate | unknown | unknown | coded unaffected | coded unaffected |
| A53 | no | Moderate | unknown | unknown | coded unaffected | coded unaffected |
| A54 | no | Moderate | unknown | unknown | coded unaffected | coded unaffected |
| A56 | no | Moderate | unknown | unknown | coded unaffected | coded unaffected |
| A59 | no | Moderate | unknown | coded unaffected | coded unaffected | excluded |
| A60 | no | Moderate | unknown | unknown | coded unaffected | coded unaffected |
| A61 | no | Moderate | unknown | coded unaffected | coded unaffected | coded unaffected |
| A63 | no | Moderate | unknown | unknown | coded unaffected | coded unaffected |
| A64 | no | Moderate | unknown | unknown | coded unaffected | coded unaffected |
| A65 | no | Moderate | unknown | coded unaffected | coded unaffected | coded unaffected |
| A66 | no | Moderate | unknown | unknown | coded unaffected | coded unaffected |
| A67 | no | Moderate | unknown | coded unaffected | coded unaffected | coded unaffected |
| A69 | no | Moderate | unknown | unknown | coded unaffected | coded unaffected |
| A70 | no | Moderate | unknown | coded unaffected | coded unaffected | coded unaffected |
| H01 | no | Moderate | unknown | unknown | unknown | unknown |
| H02 | no | High | unknown | coded unaffected | coded unaffected | coded unaffected |
| H06 | no | High | unknown | coded unaffected | coded unaffected | coded unaffected |
| H11 | no | High | unknown | unknown | coded unaffected | coded unaffected |
| H15 | no | High | unknown | unknown | coded unaffected | coded unaffected |
| H20 | no | High | unknown | unknown | coded unaffected | coded unaffected |
| H27 | no | High | unknown | coded unaffected | coded unaffected | excluded |
| H33 | no | High | unknown | coded unaffected | coded unaffected | coded unaffected |

**Table S4. Interface class counts**

| **Interface class** | **n** |
| --- | --- |
| commercial interface | 27 |
| self-hosted checkpoint | 23 |
| not a pooled dense retriever | 3 |
| checkpoint unspecified | 2 |
