# Supplementary material for: Deriving a Deployment Threshold for Benefit and Harm in Clinical AI: Multi-Institutional Decision-Analytic Modeling Study
Source: JMIR AI. 2026 Oct 23;5:e109863. doi: 10.2196/109863 (PMC13743961; doi:10.2196/109863)
Supplement: Supplementary file 9 [file ai_v5i1e109863_app9.docx]

**Code-search boundary replication**

*Replication of the published semantic code-search experiment from the authors’ released code and data, undertaken to test whether the sign-changing antecedent holds in a domain sharing no models, corpora or evaluation conventions with clinical retrieval. Baseline retrieval reproduced the published values to within 0.001 across the 18 released-checkpoint cells. Response-defined groups are present at low regularisation and absent above it, and the published mechanistic grouping does not reproduce; the generality claim is withdrawn rather than supported.*

*Configuration keys: codebert = CodeBERT; codebert_ft = fine-tuned CodeBERT, contrastively trained; codet5p = CodeT5+; codellama = Code Llama. Languages are the six CodeSearchNet languages of the published primary grid, plus R, an extension outside that grid.*

**Table S1. Baseline replication against published values**

| **Configuration** | **Language** | **Baseline MRR** | **Published baseline** | **Absolute difference** | **Analysis** |
| --- | --- | --- | --- | --- | --- |
| codebert | ruby | 0.0068 | 0.006 | 0.0008 | primary baseline replication (released checkpoints, 6 CodeSearchNet languages) |
| codebert | javascript | 0.0026 | 0.002 | 0.0006 | primary baseline replication (released checkpoints, 6 CodeSearchNet languages) |
| codebert | go | 0.0024 | 0.002 | 0.0004 | primary baseline replication (released checkpoints, 6 CodeSearchNet languages) |
| codebert | java | 0.0009 | 0.0 | 0.0009 | primary baseline replication (released checkpoints, 6 CodeSearchNet languages) |
| codebert | python | 0.001 | 0.001 | 0.0 | primary baseline replication (released checkpoints, 6 CodeSearchNet languages) |
| codebert | php | 0.0006 | 0.0 | 0.0006 | primary baseline replication (released checkpoints, 6 CodeSearchNet languages) |
| codebert | r | 0.0172 | 0.011 | 0.0062 | extension language, outside the published primary grid |
| codebert_ft | ruby | 0.5996 | 0.547 | 0.0526 | independent reimplementation (published checkpoint not released) |
| codebert_ft | javascript | 0.5188 | 0.427 | 0.0918 | independent reimplementation (published checkpoint not released) |
| codebert_ft | go | 0.6315 | 0.619 | 0.0125 | independent reimplementation (published checkpoint not released) |
| codebert_ft | java | 0.4643 | 0.395 | 0.0693 | independent reimplementation (published checkpoint not released) |
| codebert_ft | python | 0.5975 | 0.5 | 0.0975 | independent reimplementation (published checkpoint not released) |
| codebert_ft | php | 0.4254 | 0.248 | 0.1774 | independent reimplementation (published checkpoint not released) |
| codet5p | ruby | 0.706 | 0.705 | 0.001 | primary baseline replication (released checkpoints, 6 CodeSearchNet languages) |
| codet5p | javascript | 0.6384 | 0.638 | 0.0004 | primary baseline replication (released checkpoints, 6 CodeSearchNet languages) |
| codet5p | go | 0.7579 | 0.757 | 0.0009 | primary baseline replication (released checkpoints, 6 CodeSearchNet languages) |
| codet5p | java | 0.5951 | 0.595 | 0.0001 | primary baseline replication (released checkpoints, 6 CodeSearchNet languages) |
| codet5p | python | 0.7219 | 0.721 | 0.0009 | primary baseline replication (released checkpoints, 6 CodeSearchNet languages) |
| codet5p | php | 0.5371 | 0.537 | 0.0001 | primary baseline replication (released checkpoints, 6 CodeSearchNet languages) |
| codet5p | r | 0.405 | 0.045 | 0.36 | extension language, outside the published primary grid |
| codellama | ruby | 0.0479 | 0.047 | 0.0009 | primary baseline replication (released checkpoints, 6 CodeSearchNet languages) |
| codellama | javascript | 0.0262 | 0.026 | 0.0002 | primary baseline replication (released checkpoints, 6 CodeSearchNet languages) |
| codellama | go | 0.0314 | 0.031 | 0.0004 | primary baseline replication (released checkpoints, 6 CodeSearchNet languages) |
| codellama | java | 0.0159 | 0.015 | 0.0009 | primary baseline replication (released checkpoints, 6 CodeSearchNet languages) |
| codellama | python | 0.0172 | 0.017 | 0.0002 | primary baseline replication (released checkpoints, 6 CodeSearchNet languages) |
| codellama | php | 0.0097 | 0.009 | 0.0007 | primary baseline replication (released checkpoints, 6 CodeSearchNet languages) |
| codellama | r | 0.016 | 0.024 | 0.008 | extension language, outside the published primary grid |

*Note. The published-baseline criterion applies to released checkpoints. The published fine-tuned CodeBERT checkpoint was not released, so that configuration was fine-tuned here with the authors’ released script at their published settings (InfoNCE, learning rate 5e-5, batch size 32, five epochs); the resulting model exceeds the published baseline in all six languages, by 0.013 to 0.177 MRR, and baseline agreement is therefore not an applicable criterion for it. Across the 18 released-checkpoint cells the largest deviation is 0.0010. Of the three extension-language cells, CodeBERT and Code Llama on R reproduce within 0.01 through the same pipeline and corpus, while CodeT5+ on R gives 0.405 against a published 0.045; that cell is reported and carries no claim, and excluding it does not change the regularisation value at which CodeT5+ changes sign.*

**Table S2. Whitening effects by configuration and language**

| **Configuration** | **Language** | **Epsilon** | **Contrastively trained** | **Baseline MRR** | **Whitened MRR** | **dMRR** | **Analysis** |
| --- | --- | --- | --- | --- | --- | --- | --- |
| codebert | ruby | 0.0 | False | 0.0068 | 0.1269 | 0.1201 | primary replication grid |
| codebert | ruby | 0.0001 | False | 0.0068 | 0.1954 | 0.1886 | primary replication grid |
| codebert | ruby | 0.001 | False | 0.0068 | 0.2295 | 0.2227 | primary replication grid |
| codebert | ruby | 0.01 | False | 0.0068 | 0.2369 | 0.2301 | primary replication grid |
| codebert | ruby | 0.1 | False | 0.0068 | 0.1894 | 0.1827 | primary replication grid |
| codebert | ruby | 1.0 | False | 0.0068 | 0.0955 | 0.0887 | primary replication grid |
| codebert | javascript | 0.0 | False | 0.0026 | 0.0996 | 0.0971 | primary replication grid |
| codebert | javascript | 0.0001 | False | 0.0026 | 0.123 | 0.1205 | primary replication grid |
| codebert | javascript | 0.001 | False | 0.0026 | 0.141 | 0.1384 | primary replication grid |
| codebert | javascript | 0.01 | False | 0.0026 | 0.1447 | 0.1422 | primary replication grid |
| codebert | javascript | 0.1 | False | 0.0026 | 0.1044 | 0.1018 | primary replication grid |
| codebert | javascript | 1.0 | False | 0.0026 | 0.0476 | 0.045 | primary replication grid |
| codebert | go | 0.0 | False | 0.0024 | 0.0021 | -0.0003 | primary replication grid |
| codebert | go | 0.0001 | False | 0.0024 | 0.2351 | 0.2327 | primary replication grid |
| codebert | go | 0.001 | False | 0.0024 | 0.2533 | 0.2509 | primary replication grid |
| codebert | go | 0.01 | False | 0.0024 | 0.2473 | 0.2449 | primary replication grid |
| codebert | go | 0.1 | False | 0.0024 | 0.1747 | 0.1722 | primary replication grid |
| codebert | go | 1.0 | False | 0.0024 | 0.078 | 0.0756 | primary replication grid |
| codebert | java | 0.0 | False | 0.0009 | 0.0007 | -0.0002 | primary replication grid |
| codebert | java | 0.0001 | False | 0.0009 | 0.1367 | 0.1359 | primary replication grid |
| codebert | java | 0.001 | False | 0.0009 | 0.1495 | 0.1486 | primary replication grid |
| codebert | java | 0.01 | False | 0.0009 | 0.1434 | 0.1425 | primary replication grid |
| codebert | java | 0.1 | False | 0.0009 | 0.1028 | 0.1019 | primary replication grid |
| codebert | java | 1.0 | False | 0.0009 | 0.0478 | 0.0469 | primary replication grid |
| codebert | python | 0.0 | False | 0.001 | 0.0295 | 0.0285 | primary replication grid |
| codebert | python | 0.0001 | False | 0.001 | 0.1401 | 0.1391 | primary replication grid |
| codebert | python | 0.001 | False | 0.001 | 0.1571 | 0.156 | primary replication grid |
| codebert | python | 0.01 | False | 0.001 | 0.1578 | 0.1567 | primary replication grid |
| codebert | python | 0.1 | False | 0.001 | 0.1101 | 0.1091 | primary replication grid |
| codebert | python | 1.0 | False | 0.001 | 0.0504 | 0.0494 | primary replication grid |
| codebert | php | 0.0 | False | 0.0006 | 0.0005 | -0.0001 | primary replication grid |
| codebert | php | 0.0001 | False | 0.0006 | 0.0936 | 0.093 | primary replication grid |
| codebert | php | 0.001 | False | 0.0006 | 0.1028 | 0.1022 | primary replication grid |
| codebert | php | 0.01 | False | 0.0006 | 0.0997 | 0.099 | primary replication grid |
| codebert | php | 0.1 | False | 0.0006 | 0.0684 | 0.0678 | primary replication grid |
| codebert | php | 1.0 | False | 0.0006 | 0.0287 | 0.0281 | primary replication grid |
| codebert | r | 0.0 | False | 0.0172 | 0.0927 | 0.0754 | extended regularisation analysis |
| codebert | r | 0.0001 | False | 0.0172 | 0.1167 | 0.0994 | extended regularisation analysis |
| codebert | r | 0.001 | False | 0.0172 | 0.139 | 0.1217 | extended regularisation analysis |
| codebert | r | 0.01 | False | 0.0172 | 0.1394 | 0.1222 | extended regularisation analysis |
| codebert | r | 0.1 | False | 0.0172 | 0.1112 | 0.094 | extended regularisation analysis |
| codebert | r | 1.0 | False | 0.0172 | 0.0652 | 0.048 | extended regularisation analysis |
| codebert_ft | ruby | 0.0 | True | 0.5996 | 0.6529 | 0.0532 | extended regularisation analysis |
| codebert_ft | ruby | 0.0001 | True | 0.5996 | 0.653 | 0.0533 | extended regularisation analysis |
| codebert_ft | ruby | 0.001 | True | 0.5996 | 0.6545 | 0.0549 | extended regularisation analysis |
| codebert_ft | ruby | 0.01 | True | 0.5996 | 0.6528 | 0.0532 | extended regularisation analysis |
| codebert_ft | ruby | 0.1 | True | 0.5996 | 0.6414 | 0.0417 | extended regularisation analysis |
| codebert_ft | ruby | 1.0 | True | 0.5996 | 0.6259 | 0.0263 | extended regularisation analysis |
| codebert_ft | javascript | 0.0 | True | 0.5188 | 0.5644 | 0.0456 | extended regularisation analysis |
| codebert_ft | javascript | 0.0001 | True | 0.5188 | 0.5649 | 0.0461 | extended regularisation analysis |
| codebert_ft | javascript | 0.001 | True | 0.5188 | 0.565 | 0.0463 | extended regularisation analysis |
| codebert_ft | javascript | 0.01 | True | 0.5188 | 0.5619 | 0.0431 | extended regularisation analysis |
| codebert_ft | javascript | 0.1 | True | 0.5188 | 0.5514 | 0.0326 | extended regularisation analysis |
| codebert_ft | javascript | 1.0 | True | 0.5188 | 0.5379 | 0.0192 | extended regularisation analysis |
| codebert_ft | go | 0.0 | True | 0.6315 | 0.5761 | -0.0554 | extended regularisation analysis |
| codebert_ft | go | 0.0001 | True | 0.6315 | 0.6823 | 0.0508 | extended regularisation analysis |
| codebert_ft | go | 0.001 | True | 0.6315 | 0.6819 | 0.0504 | extended regularisation analysis |
| codebert_ft | go | 0.01 | True | 0.6315 | 0.679 | 0.0475 | extended regularisation analysis |
| codebert_ft | go | 0.1 | True | 0.6315 | 0.6698 | 0.0383 | extended regularisation analysis |
| codebert_ft | go | 1.0 | True | 0.6315 | 0.658 | 0.0265 | extended regularisation analysis |
| codebert_ft | java | 0.0 | True | 0.4643 | 0.5109 | 0.0466 | extended regularisation analysis |
| codebert_ft | java | 0.0001 | True | 0.4643 | 0.5109 | 0.0466 | extended regularisation analysis |
| codebert_ft | java | 0.001 | True | 0.4643 | 0.5104 | 0.046 | extended regularisation analysis |
| codebert_ft | java | 0.01 | True | 0.4643 | 0.5068 | 0.0425 | extended regularisation analysis |
| codebert_ft | java | 0.1 | True | 0.4643 | 0.4958 | 0.0315 | extended regularisation analysis |
| codebert_ft | java | 1.0 | True | 0.4643 | 0.4824 | 0.0181 | extended regularisation analysis |
| codebert_ft | python | 0.0 | True | 0.5975 | 0.6379 | 0.0404 | extended regularisation analysis |
| codebert_ft | python | 0.0001 | True | 0.5975 | 0.6382 | 0.0407 | extended regularisation analysis |
| codebert_ft | python | 0.001 | True | 0.5975 | 0.6371 | 0.0396 | extended regularisation analysis |
| codebert_ft | python | 0.01 | True | 0.5975 | 0.6315 | 0.0341 | extended regularisation analysis |
| codebert_ft | python | 0.1 | True | 0.5975 | 0.6193 | 0.0219 | extended regularisation analysis |
| codebert_ft | python | 1.0 | True | 0.5975 | 0.6075 | 0.01 | extended regularisation analysis |
| codebert_ft | php | 0.0 | True | 0.4254 | 0.4661 | 0.0406 | extended regularisation analysis |
| codebert_ft | php | 0.0001 | True | 0.4254 | 0.4661 | 0.0407 | extended regularisation analysis |
| codebert_ft | php | 0.001 | True | 0.4254 | 0.4656 | 0.0401 | extended regularisation analysis |
| codebert_ft | php | 0.01 | True | 0.4254 | 0.4604 | 0.0349 | extended regularisation analysis |
| codebert_ft | php | 0.1 | True | 0.4254 | 0.4479 | 0.0224 | extended regularisation analysis |
| codebert_ft | php | 1.0 | True | 0.4254 | 0.4377 | 0.0123 | extended regularisation analysis |
| codet5p | ruby | 0.0 | True | 0.706 | 0.6727 | -0.0332 | primary replication grid |
| codet5p | ruby | 0.0001 | True | 0.706 | 0.6924 | -0.0136 | primary replication grid |
| codet5p | ruby | 0.001 | True | 0.706 | 0.7014 | -0.0046 | primary replication grid |
| codet5p | ruby | 0.01 | True | 0.706 | 0.7127 | 0.0067 | primary replication grid |
| codet5p | ruby | 0.1 | True | 0.706 | 0.7129 | 0.0069 | primary replication grid |
| codet5p | ruby | 1.0 | True | 0.706 | 0.7112 | 0.0052 | primary replication grid |
| codet5p | javascript | 0.0 | True | 0.6384 | 0.5943 | -0.044 | primary replication grid |
| codet5p | javascript | 0.0001 | True | 0.6384 | 0.616 | -0.0224 | primary replication grid |
| codet5p | javascript | 0.001 | True | 0.6384 | 0.6282 | -0.0101 | primary replication grid |
| codet5p | javascript | 0.01 | True | 0.6384 | 0.6395 | 0.0011 | primary replication grid |
| codet5p | javascript | 0.1 | True | 0.6384 | 0.6418 | 0.0034 | primary replication grid |
| codet5p | javascript | 1.0 | True | 0.6384 | 0.6411 | 0.0028 | primary replication grid |
| codet5p | go | 0.0 | True | 0.7579 | 0.7377 | -0.0201 | primary replication grid |
| codet5p | go | 0.0001 | True | 0.7579 | 0.747 | -0.0108 | primary replication grid |
| codet5p | go | 0.001 | True | 0.7579 | 0.7513 | -0.0065 | primary replication grid |
| codet5p | go | 0.01 | True | 0.7579 | 0.7562 | -0.0017 | primary replication grid |
| codet5p | go | 0.1 | True | 0.7579 | 0.7577 | -0.0002 | primary replication grid |
| codet5p | go | 1.0 | True | 0.7579 | 0.7573 | -0.0005 | primary replication grid |
| codet5p | java | 0.0 | True | 0.5951 | 0.5583 | -0.0367 | primary replication grid |
| codet5p | java | 0.0001 | True | 0.5951 | 0.578 | -0.0171 | primary replication grid |
| codet5p | java | 0.001 | True | 0.5951 | 0.5871 | -0.008 | primary replication grid |
| codet5p | java | 0.01 | True | 0.5951 | 0.5977 | 0.0026 | primary replication grid |
| codet5p | java | 0.1 | True | 0.5951 | 0.5975 | 0.0024 | primary replication grid |
| codet5p | java | 1.0 | True | 0.5951 | 0.5964 | 0.0013 | primary replication grid |
| codet5p | python | 0.0 | True | 0.7219 | 0.6772 | -0.0446 | primary replication grid |
| codet5p | python | 0.0001 | True | 0.7219 | 0.6968 | -0.0251 | primary replication grid |
| codet5p | python | 0.001 | True | 0.7219 | 0.7083 | -0.0136 | primary replication grid |
| codet5p | python | 0.01 | True | 0.7219 | 0.7207 | -0.0011 | primary replication grid |
| codet5p | python | 0.1 | True | 0.7219 | 0.7224 | 0.0006 | primary replication grid |
| codet5p | python | 1.0 | True | 0.7219 | 0.7213 | -0.0006 | primary replication grid |
| codet5p | php | 0.0 | True | 0.5371 | 0.4845 | -0.0526 | primary replication grid |
| codet5p | php | 0.0001 | True | 0.5371 | 0.5082 | -0.029 | primary replication grid |
| codet5p | php | 0.001 | True | 0.5371 | 0.5217 | -0.0154 | primary replication grid |
| codet5p | php | 0.01 | True | 0.5371 | 0.5372 | 0.0001 | primary replication grid |
| codet5p | php | 0.1 | True | 0.5371 | 0.5394 | 0.0023 | primary replication grid |
| codet5p | php | 1.0 | True | 0.5371 | 0.5386 | 0.0014 | primary replication grid |
| codet5p | r | 0.0 | True | 0.405 | 0.4164 | 0.0114 | extended regularisation analysis |
| codet5p | r | 0.0001 | True | 0.405 | 0.4443 | 0.0393 | extended regularisation analysis |
| codet5p | r | 0.001 | True | 0.405 | 0.4554 | 0.0504 | extended regularisation analysis |
| codet5p | r | 0.01 | True | 0.405 | 0.4523 | 0.0472 | extended regularisation analysis |
| codet5p | r | 0.1 | True | 0.405 | 0.4466 | 0.0416 | extended regularisation analysis |
| codet5p | r | 1.0 | True | 0.405 | 0.4416 | 0.0366 | extended regularisation analysis |
| codellama | ruby | 0.0 | False | 0.0479 | 0.3363 | 0.2884 | primary replication grid |
| codellama | ruby | 0.0001 | False | 0.0479 | 0.4871 | 0.4392 | primary replication grid |
| codellama | ruby | 0.001 | False | 0.0479 | 0.4988 | 0.4509 | primary replication grid |
| codellama | ruby | 0.01 | False | 0.0479 | 0.5169 | 0.4691 | primary replication grid |
| codellama | ruby | 0.1 | False | 0.0479 | 0.5245 | 0.4766 | primary replication grid |
| codellama | ruby | 1.0 | False | 0.0479 | 0.4923 | 0.4444 | primary replication grid |
| codellama | javascript | 0.0 | False | 0.0262 | 0.3532 | 0.327 | primary replication grid |
| codellama | javascript | 0.0001 | False | 0.0262 | 0.3574 | 0.3312 | primary replication grid |
| codellama | javascript | 0.001 | False | 0.0262 | 0.3726 | 0.3464 | primary replication grid |
| codellama | javascript | 0.01 | False | 0.0262 | 0.3914 | 0.3652 | primary replication grid |
| codellama | javascript | 0.1 | False | 0.0262 | 0.3959 | 0.3697 | primary replication grid |
| codellama | javascript | 1.0 | False | 0.0262 | 0.3618 | 0.3356 | primary replication grid |
| codellama | go | 0.0 | False | 0.0314 | 0.4729 | 0.4415 | primary replication grid |
| codellama | go | 0.0001 | False | 0.0314 | 0.4745 | 0.4431 | primary replication grid |
| codellama | go | 0.001 | False | 0.0314 | 0.4831 | 0.4516 | primary replication grid |
| codellama | go | 0.01 | False | 0.0314 | 0.4972 | 0.4658 | primary replication grid |
| codellama | go | 0.1 | False | 0.0314 | 0.4969 | 0.4655 | primary replication grid |
| codellama | go | 1.0 | False | 0.0314 | 0.4648 | 0.4334 | primary replication grid |
| codellama | java | 0.0 | False | 0.0159 | 0.3305 | 0.3146 | primary replication grid |
| codellama | java | 0.0001 | False | 0.0159 | 0.3313 | 0.3154 | primary replication grid |
| codellama | java | 0.001 | False | 0.0159 | 0.3362 | 0.3203 | primary replication grid |
| codellama | java | 0.01 | False | 0.0159 | 0.345 | 0.3291 | primary replication grid |
| codellama | java | 0.1 | False | 0.0159 | 0.3431 | 0.3272 | primary replication grid |
| codellama | java | 1.0 | False | 0.0159 | 0.3013 | 0.2855 | primary replication grid |
| codellama | python | 0.0 | False | 0.0172 | 0.3943 | 0.3771 | primary replication grid |
| codellama | python | 0.0001 | False | 0.0172 | 0.3951 | 0.3779 | primary replication grid |
| codellama | python | 0.001 | False | 0.0172 | 0.4008 | 0.3836 | primary replication grid |
| codellama | python | 0.01 | False | 0.0172 | 0.4125 | 0.3953 | primary replication grid |
| codellama | python | 0.1 | False | 0.0172 | 0.4163 | 0.3992 | primary replication grid |
| codellama | python | 1.0 | False | 0.0172 | 0.3834 | 0.3662 | primary replication grid |
| codellama | php | 0.0 | False | 0.0097 | 0.2177 | 0.208 | primary replication grid |
| codellama | php | 0.0001 | False | 0.0097 | 0.2186 | 0.2088 | primary replication grid |
| codellama | php | 0.001 | False | 0.0097 | 0.2241 | 0.2144 | primary replication grid |
| codellama | php | 0.01 | False | 0.0097 | 0.2347 | 0.225 | primary replication grid |
| codellama | php | 0.1 | False | 0.0097 | 0.2371 | 0.2274 | primary replication grid |
| codellama | php | 1.0 | False | 0.0097 | 0.2079 | 0.1982 | primary replication grid |
| codellama | r | 0.0 | False | 0.016 | 0.1054 | 0.0894 | extended regularisation analysis |
| codellama | r | 0.0001 | False | 0.016 | 0.512 | 0.496 | extended regularisation analysis |
| codellama | r | 0.001 | False | 0.016 | 0.5155 | 0.4995 | extended regularisation analysis |
| codellama | r | 0.01 | False | 0.016 | 0.5228 | 0.5068 | extended regularisation analysis |
| codellama | r | 0.1 | False | 0.016 | 0.5066 | 0.4906 | extended regularisation analysis |
| codellama | r | 1.0 | False | 0.016 | 0.4209 | 0.4049 | extended regularisation analysis |

**Table S3. Regularisation grid**

| **Configuration** | **0.0** | **0.0001** | **0.001** | **0.01** | **0.1** | **1.0** |
| --- | --- | --- | --- | --- | --- | --- |
| codebert | 0.045785714285714284 | 0.14417142857142856 | 0.16292857142857145 | 0.1625142857142857 | 0.11850000000000001 | 0.054528571428571426 |
| codebert_ft | 0.028499999999999998 | 0.04636666666666667 | 0.04621666666666666 | 0.04255 | 0.031400000000000004 | 0.018733333333333334 |
| codellama | 0.29228571428571426 | 0.3730857142857143 | 0.3809571428571429 | 0.39375714285714286 | 0.3937428571428571 | 0.35259999999999997 |
| codet5p | -0.0314 | -0.011242857142857143 | -0.0011142857142857146 | 0.007842857142857143 | 0.008142857142857143 | 0.0066 |

*Note. At zero regularisation three configuration means are positive and CodeT5+ is negative, so response-defined groups are both defined. Fine-tuned CodeBERT remains positive at every tested regularisation value, whereas CodeT5+ crosses from negative to positive between 0.001 and 0.01; above that the harmed group is empty and no threshold is defined.*
